# Supplementary material for: Healthcare utilisation in patients with long-term conditions during the COVID-19 pandemic: a population-based observational study of all patients across Greater Manchester, UK
Source: BMJ Open. 2023 Jul 7;13(7):e066873. doi: 10.1136/bmjopen-2022-066873 (PMC10335594; doi:10.1136/bmjopen-2022-066873)
Supplement: Supplementary data [file bmjopen-2022-066873supp001.pdf]

SUPPLEMENTARY MATERIALS

COVID-19 Government data extraction

The Government data were extracted on 08-09-2021 from https://api.coronavirus.data.gov.uk/ . For the new cases, we extracted data for Manchester only. The API for Manchester cases by specimen date that was used is:

https://api.coronavirus.data.gov.uk/v2/data?areaType=utla&areaCode=E08000003&metric=newCasesBySpecimenDate&format=csv

For the secondary admissions, we exported the data for the trusts which were the main providers in the Manchester CCG subpopulation. The hospital groupings were not consistent across data sources; Table S2 provides the mapping between the Government listed NHS trusts and the secondary providers covering nearly all admissions experienced by Manchester CCG population in the GMCR.

Supplementary Table S1: Mapping between NHS acute providers according to data source

GMCR acute provider	Government listed NHS Trust	Government data API
University Hospital of South Manchester	Manchester University NHS Foundation Trust	https://api.coronavirus.data.gov.uk/v2/data?areaType=nhsTrust&areaCode=R0A&metric=newAdmissions&format=csv
Central Manchester University Hospitals		
Pennine Acute Hospitals	Pennine Acute Hospitals NHS Trust	https://api.coronavirus.data.gov.uk/v2/data?areaType=nhsTrust&areaCode=RW6&metric=newAdmissions&format=csv
Pennine Acute Hospitals	Pennine Care NHS Foundation Trust	https://api.coronavirus.data.gov.uk/v2/data?areaType=nhsTrust&areaCode=RT2&metric=newAdmissions&format=csv

Supplementary Table S2: Long-term medical conditions and their groupings

Grouping	Long-term medical condition
**Cardiovascular**	Hypertension<br>Atrial fibrillation<br>Heart failure<br>Peripheral vascular disease<br>Stroke & transient ischaemic attack<br>Coronary heart disease
**Respiratory**	Bronchiectasis<br>Asthma<br>Chronic obstructive pulmonary disease<br>Chronic sinusitis
**Gastrointestinal**	Viral Hepatitis<br>Chronic liver disease<br>Inflammatory bowel disease<br>Diverticular disease of intestine<br>Treated constipation<br>Irritable bowel syndrome<br>Treated dyspepsia
**Neurological**	Multiple sclerosis<br>Parkinson's disease<br>Migraine<br>Epilepsy
**Endocrine**	Thyroid disorders<br>Diabetes
**Psychiatric**	Anorexia or bulimia<br>Schizophrenia (and related non-organic psychosis) or bipolar disorder<br>Dementia<br>Anxiety & other neurotic, stress related & somatoform disorders<br>Depression
**Substance Abuse**	Alcohol problems<br>Other psychoactive substance misuse
**Musculoskeletal/Skin**	Psoriasis or eczema<br>Rheumatoid arthritis, other inflammatory polyarthropathies & systematic connective tissue disorders<br>Painful condition
**Sensory impairment or learning disability**	Learning disability<br>Blindness & low vision<br>Glaucoma<br>Hearing loss
**Renal/Urological**	Chronic kidney disease<br>Prostate disorders

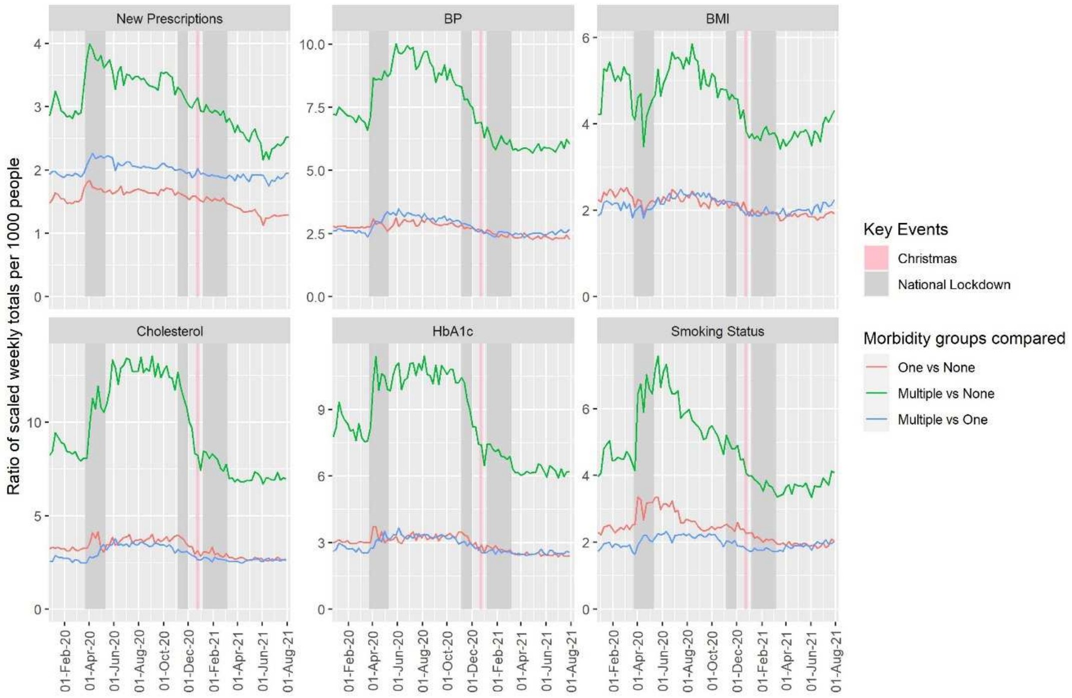

Supplementary Figure S1: Ratio of weekly primary care HCU measures per 1000 people between morbidity groups, between January 2020 and August 2021.

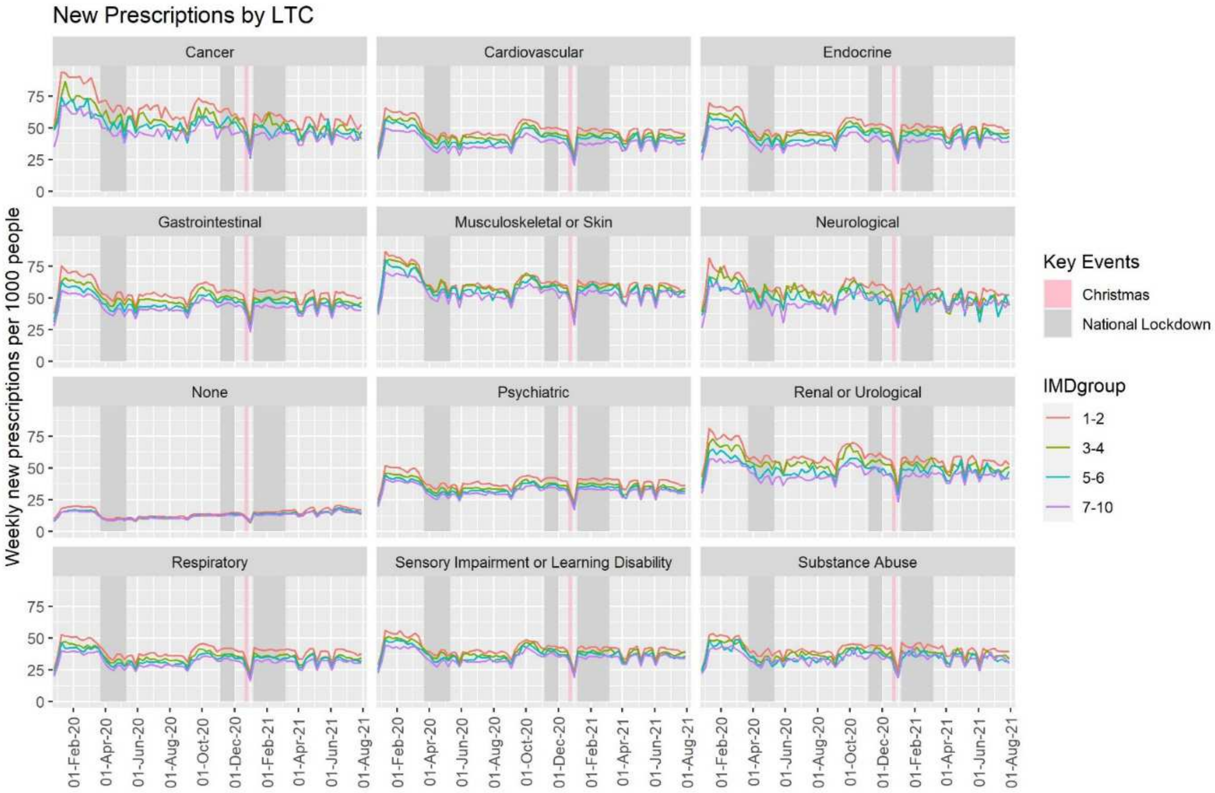

Supplementary Figure S2: Weekly new primary care prescriptions per 1000 people within each long-term condition group and deprivation group, between January 2020 and August 2021.

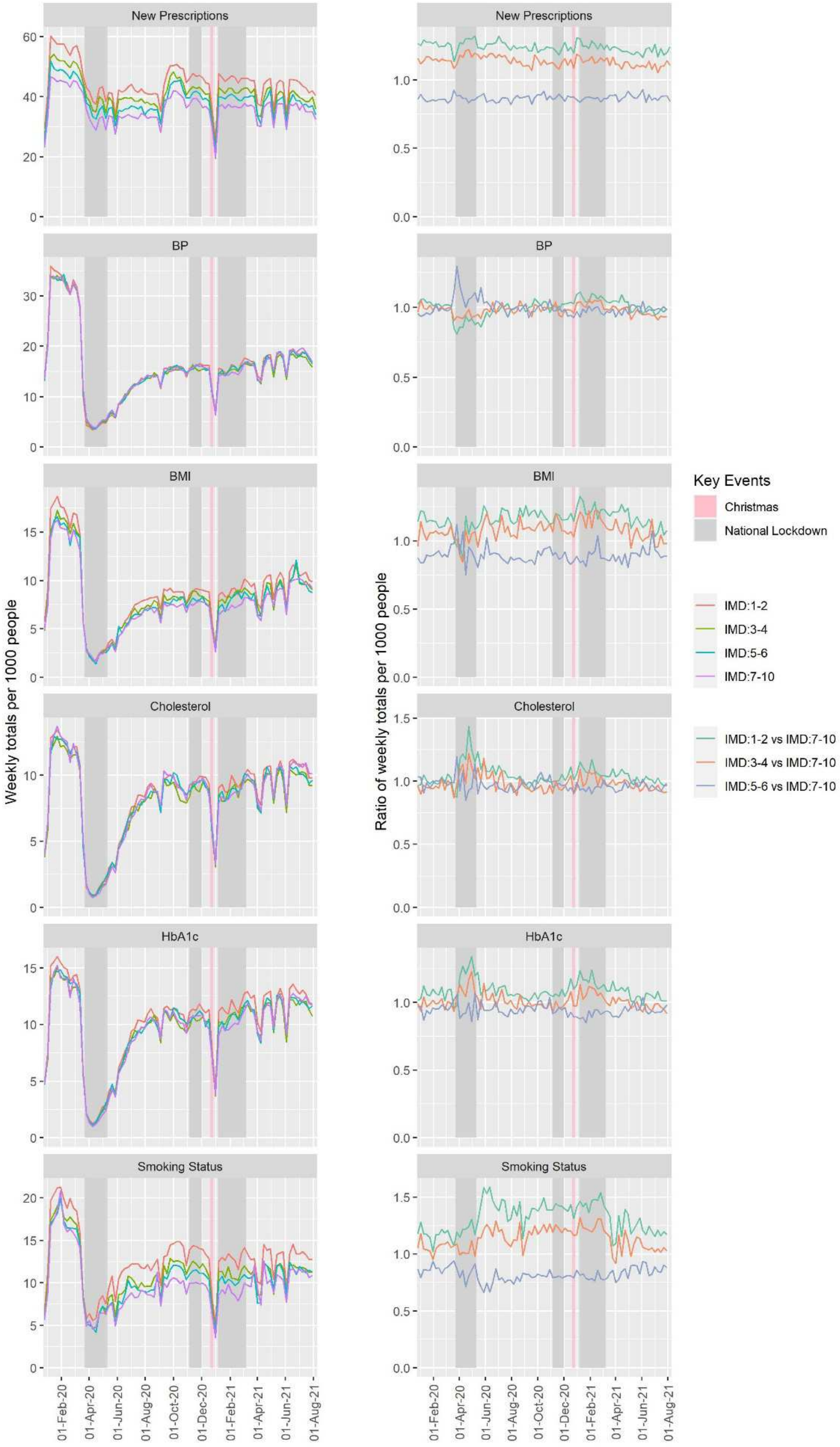

Supplementary Figure S3: Weekly new primary care HCU measures per 1000 people within multi-morbid patients by deprivation group and the ratio of these compared to the least-deprived group (IMD: 7-10), between January 2020 and August 2021.

Supplementary Table S3: Associated effects of the national lockdowns on primary HCU compare to pre-pandemic HCU. NL1 = First national lockdown, NL2 = Second national lockdown, NL3 = Third national lockdown.

	Rate Ratio	95% CI	p-value
	**New Prescriptions**		
NL1 vs pre-pandemic	0.681	0.679 – 0.684	**<0.001**
NL2 vs pre-pandemic	0.835	0.832 – 0.838	**<0.001**
NL3 vs pre-pandemic	0.843	0.841 – 0.846	**<0.001**
	**BP**		
NL1 vs pre-pandemic	0.143	0.142 – 0.144	**<0.001**
NL2 vs pre-pandemic	0.494	0.491 – 0.497	**<0.001**
NL3 vs pre-pandemic	0.510	0.508 – 0.513	**<0.001**
	**BMI**		
NL1 vs pre-pandemic	0.168	0.167 – 0.170	**<0.001**
NL2 vs pre-pandemic	0.559	0.555 – 0.564	**<0.001**
NL3 vs pre-pandemic	0.592	0.588 – 0.596	**<0.001**
	**Cholesterol**		
NL1 vs pre-pandemic	0.128	0.126 – 0.130	**<0.001**
NL2 vs pre-pandemic	0.769	0.762 – 0.776	**<0.001**
NL3 vs pre-pandemic	0.788	0.782 – 0.793	**<0.001**
	**HbA1c**		
NL1 vs pre-pandemic	0.148	0.146 – 0.150	**<0.001**
NL2 vs pre-pandemic	0.785	0.778 – 0.791	**<0.001**
NL3 vs pre-pandemic	0.837	0.832 – 0.842	**<0.001**
	**Smoking Status**		
NL1 vs pre-pandemic	0.346	0.344 – 0.349	**<0.001**
NL2 vs pre-pandemic	0.692	0.687 – 0.696	**<0.001**
NL3 vs pre-pandemic	0.689	0.685 – 0.692	**<0.001**
	**Planned Admissions**		
NL1 vs pre-pandemic	0.544	0.530 – 0.559	**<0.001**
NL2 vs pre-pandemic	0.921	0.897 – 0.946	**<0.001**
NL3 vs pre-pandemic	0.902	0.883 – 0.922	**<0.001**
	**Unplanned Admissions**		
NL1 vs pre-pandemic	0.659	0.638 – 0.681	**<0.001**
NL2 vs pre-pandemic	0.953	0.922 – 0.986	**0.005**
NL3 vs pre-pandemic	0.984	0.958 – 1.010	0.227

Supplementary Table S4: Percentage decrease (95%CI) in HCU in 2021 compared with the same calendar week in 2020.

Calendar Week	2	3	4	5	6	7	8	9	10	11	Average	p-value
**Primary HCU**												
New prescriptions	20.0 (19.3 – 20.8)	21.2 (20.5 – 21.9)	18.4 (17.7 – 19.2)	18.7 (18.0 – 19.5)	19.6 (18.9 – 20.3)	18.7 (17.9 – 19.5)	15.5 (14.8 – 16.3)	15.5 (14.8 – 16.3)	17.2 (16.5 – 18.0)	12.8 (12.0 – 13.6)	17.8 (17.6 – 18.1)	<0.001
Blood Pressure	55.2 (54.5 – 55.9)	55.8 (55.1 – 56.5)	54.4 (53.7 – 55.1)	52.0 (51.3 – 52.7)	52.4 (51.6 – 53.1)	52.1 (51.4 – 52.9)	45.9 (45.0 – 46.7)	46.4 (45.7 – 47.2)	44.3 (43.4 – 45.1)	38.7 (37.8 – 39.7)	50.0 (49.8 – 50.2)	<0.001
BMI	44.4 (43.3 – 45.5)	49.7 (48.7 – 50.8)	49.7 (48.7 – 50.8)	45.3 (44.3 – 46.5)	45.7 (44.6 – 46.8)	43.2 (42.0 – 44.3)	37.8 (36.6 – 39.1)	36.7 (35.6 – 38.0)	36.3 (35.1 – 37.6)	32.6 (31.3 – 34.0)	42.5 (42.1 – 42.8)	<0.001
Cholesterol	27.5 (25.8 – 29.1)	34.0 (32.4 – 35.4)	34.2 (32.7 – 35.6)	26.9 (25.3 – 28.5)	26.2 (24.5 – 27.7)	27.4 (25.8 – 29.1)	17.1 (15.3 – 19.0)	18.0 (16.2 – 19.8)	11.9 (10.0 – 13.8)	6.3 (4.2 – 8.4)	18.4 (17.8 – 18.9)	<0.001
HbA1c	22.0 (20.4 – 23.6)	28.6 (27.2 – 30.1)	29.4 (27.9 – 30.8)	23.0 (21.5 – 24.6)	21.7 (20.1 – 23.2)	24.1 (22.6 – 25.7)	12.4 (10.6 – 14.2)	10.7 (9.0 – 12.5)	7.7 (5.9 – 9.5)	2.7 (0.8 – 4.7)	18.6 (18.1 – 19.1)	<0.001
Smoking Status	33.3 (32.1 – 34.5)	37.8 (36.7 – 38.9)	34.7 (33.6 – 35.9)	43.4 (42.6 – 44.5)	38.9 (37.8 – 40.0)	38.4 (37.3 – 39.6)	32.9 (31.8 – 34.2)	24.7 (23.5 – 26.1)	25.1 (23.9 – 26.6)	18.8 (17.4 – 20.4)	33.3 (33.0 – 33.7)	<0.001
**Secondary HCU**												
Planned admissions	11.6 (5.3 – 17.5)	16.8 (10.9 – 22.3)	12.5 (6.3 – 18.2)	-0.4 (-7.6 – 6.3)	27.1 (21.6 – 32.3)	12.5 (6.2 – 18.4)	1.7 (-5.2 – 8.2)	12.5 (6.3 – 18.3)	14.8 (8.9 – 20.4)	2.6 (-4.1 – 8.9)	11.3 (9.4 – 13.2)	<0.001
Unplanned admissions	8.4 (0.2 – 15.9)	3.3 (-5.2 – 11.2)	11.2 (-7.4 – 9.1)	-14.2 (-24.9 – -4.4)	26.9 (19.4 – 33.8)	2.0 (-6.8 – 10.1)	-7.7 (-17.2 – 1.1)	-5.7 (-15.1 – 2.9)	-3.1 (-12.2 – 5.2)	-25.7 (-37.0 – -15.5)	-1.2 (-4.0 – 1.5)	0.376

Supplementary Table S5: Associated effects of morbidity on the rates of weekly totals of primary and secondary HCU (per 1000 people), throughout the study period. LTC = Long term condition.

	Rate Ratio	95% CI	p-value
	**New Prescriptions**		
Multiple LTCs vs No LTCs	3.040	2.881 – 3.208	<0.001
One LTC vs No LTCs	1.534	1.454 – 1.619	<0.001
	**BP**		
Multiple LTCs vs No LTCs	7.347	6.197 – 8.711	<0.001
One LTC vs No LTCs	2.659	2.243 – 3.153	<0.001
	**BMI**		
Multiple LTCs vs No LTCs	4.429	3.801 – 5.162	<0.001
One LTC vs No LTCs	2.106	1.807 – 2.454	<0.001
	**Cholesterol**		
Multiple LTCs vs No LTCs	9.360	7.654 – 11.446	<0.001
One LTC vs No LTCs	3.226	2.638 – 3.945	<0.001
	**HbA1c**		
Multiple LTCs vs No LTCs	8.291	6.847 – 10.038	<0.001
One LTC vs No LTCs	2.918	2.410 – 3.533	<0.001
	**Smoking Status**		
Multiple LTCs vs No LTCs	4.674	4.192 – 5.211	<0.001
One LTC vs No LTCs	2.372	2.127 – 2.645	<0.001
	**Planned Admissions**		
Multiple LTCs vs No LTCs	9.584	8.644 – 10.627	<0.001
One LTC vs No LTCs	1.904	1.717 – 2.111	<0.001
	**Unplanned Admissions**		
Multiple LTCs vs No LTCs	3.636	3.401 – 3.887	<0.001
One LTC vs No LTCs	1.188	1.112 – 1.270	<0.001

Supplementary Table S6: Estimated rate ratios (RRs) from log-linear regression models for each HCU measure observed pre-pandemic and in the national lockdowns, adjusted for the number of long term conditions (LTCs). NL1 = First national lockdown, NL2 = Second national lockdown, NL3 = Third national lockdown.

	**Primary Care**											
	**New Prescriptions**			**BP**			**BMI**			**Cholesterol**		
	RR	95% CI	p-value	RR	95% CI	p-value	RR	95% CI	p-value	RR	95% CI	p-value
NL1 vs pre-pandemic	0.580	0.543 - 0.619	**<0.001**	0.126	0.112 - 0.141	**<0.001**	0.190	0.165 - 0.218	**<0.001**	0.103	0.084 - 0.127	**<0.001**
NL2 vs pre-pandemic	0.800	0.742 - 0.862	**<0.001**	0.469	0.409 - 0.537	**<0.001**	0.615	0.523 - 0.722	**<0.001**	0.624	0.490 - 0.796	**<0.001**
NL3 vs pre-pandemic	0.855	0.804 - 0.909	**<0.001**	0.581	0.520 - 0.648	**<0.001**	0.752	0.660 - 0.856	**<0.001**	0.859	0.705 - 1.046	**0.128**
Multiple LTCs vs No LTCs	2.950	2.779 - 3.131	**<0.001**	7.155	6.429 - 7.963	**<0.001**	5.091	4.483 - 5.781	**<0.001**	8.576	7.077 - 10.392	**<0.001**
One LTC vs No LTCs	1.531	1.442 - 1.625	**<0.001**	2.753	2.474 - 3.064	**<0.001**	2.400	2.114 - 2.726	**<0.001**	3.222	2.659 - 3.904	**<0.001**
NL1 vs pre-pandemic : Multiple LTCs vs No LTCs	1.281	1.169 - 1.404	**<0.001**	1.187	1.007 - 1.400	**0.042**	0.847	0.696 - 1.030	0.095	1.220	0.907 - 1.640	0.186
NL2 vs pre-pandemic : Multiple LTCs vs No LTCs	1.073	0.964 - 1.193	0.194	1.091	0.901 - 1.321	0.369	0.911	0.726 - 1.144	0.418	1.318	0.934 - 1.858	0.114
NL3 vs pre-pandemic : Multiple LTCs vs No LTCs	0.984	0.903 - 1.073	0.713	0.859	0.736 - 1.004	0.056	0.736	0.613 - 0.885	**0.001**	0.920	0.697 - 1.216	0.555
NL1 vs pre-pandemic : One LTC vs No LTCs	1.126	1.027 - 1.234	**0.012**	1.025	0.870 - 1.209	0.763	0.889	0.731 - 1.081	0.236	1.102	0.820 - 1.482	0.515
NL2 vs pre-pandemic : One LTC vs No LTCs	1.043	0.937 - 1.160	0.439	0.996	0.823 - 1.206	0.968	0.888	0.708 - 1.115	0.304	1.139	0.808 - 1.606	0.454
NL3 vs pre-pandemic : One LTC vs No LTCs	0.994	0.912 - 1.084	0.895	0.896	0.767 - 1.046	0.162	0.811	0.674 - 0.975	**0.026**	0.921	0.697 - 1.216	0.557
	**Primary Care (ctd)**						**Secondary Care**					
	**HbA1c**			**Smoking Status**			**Planned Admissions**			**Unplanned Admissions**		
	RR	95% CI	p-value	RR	95% CI	p-value	RR	95% CI	p-value	RR	95% CI	p-value

NL1 vs pre-pandemic	0.120	0.096 - 0.148	**<0.001**	0.272	0.238 - 0.310	**<0.001**	0.272	0.243 - 0.305	**<0.001**	0.582	0.511 - 0.663	**<0.001**
NL2 vs pre-pandemic	0.716	0.557 - 0.920	**0.010**	0.667	0.573 - 0.777	**<0.001**	0.820	0.719 - 0.935	**0.003**	0.803	0.690 - 0.934	**0.005**
NL3 vs pre-pandemic	0.967	0.789 - 1.185	0.745	0.816	0.721 - 0.923	**<0.001**	0.935	0.841 - 1.040	0.214	0.933	0.826 - 1.055	0.267
Multiple LTCs vs No LTCs	8.223	6.744 - 10.026	**<0.001**	4.595	4.074 - 5.183	**<0.001**	8.298	7.481 - 9.205	**<0.001**	3.359	2.981 - 3.786	**<0.001**
One LTC vs No LTCs	3.022	2.478 - 3.684	**<0.001**	2.404	2.131 - 2.712	**<0.001**	1.834	1.653 - 2.034	**<0.001**	1.111	0.986 - 1.252	0.083
NL1 vs pre-pandemic : Multiple LTCs vs No LTCs	1.221	0.900 - 1.658	0.197	1.356	1.126 - 1.632	**0.002**	2.402	2.047 - 2.818	**<0.001**	1.253	1.042 - 1.506	**0.017**
NL2 vs pre-pandemic : Multiple LTCs vs No LTCs	1.145	0.803 - 1.633	0.449	1.074	0.866 - 1.332	0.511	1.174	0.975 - 1.413	0.090	1.283	1.036 - 1.588	**0.023**
NL3 vs pre-pandemic : Multiple LTCs vs No LTCs	0.852	0.639 - 1.136	0.271	0.803	0.675 - 0.956	**0.014**	0.957	0.824 - 1.112	0.563	1.073	0.902 - 1.276	0.421
NL1 vs pre-pandemic : One LTC vs No LTCs	1.082	0.797 - 1.468	0.610	1.279	1.062 - 1.540	**0.010**	1.413	1.205 - 1.658	**<0.001**	0.987	0.821 - 1.186	0.885
NL2 vs pre-pandemic : One LTC vs No LTCs	1.058	0.742 - 1.508	0.754	1.028	0.829 - 1.275	0.799	1.063	0.883 - 1.280	0.513	1.295	1.046 - 1.604	**0.018**
NL3 vs pre-pandemic : One LTC vs No LTCs	0.894	0.671 - 1.192	0.442	0.87	0.730 - 1.036	0.116	0.971	0.835 - 1.128	0.695	1.075	0.904 - 1.279	0.407

Supplementary Table S7: Associated effects of deprivation groups on primary and secondary HCU, throughout the study.

	**Primary Care**											
Deprivation group compared to group 1-2	**New Prescriptions**			**BP**			**BMI**			**Cholesterol**		
	RR	95% CI	p-value	RR	95% CI	p-value	RR	95% CI	p-value	RR	95% CI	p-value
3-4	0.915	0.874 – 0.959	**<0.001**	1.018	0.869 – 1.193	0.821	1.007	0.865 – 1.172	0.928	0.964	0.797 – 1.166	0.702
5-6	0.920	0.878 – 0.964	**<0.001**	1.117	0.954 – 1.309	0.169	1.025	0.881 – 1.192	0.751	1.076	0.889 – 1.302	0.450
7-10	0.875	0.835 – 0.917	**<0.001**	1.134	0.968 – 1.328	0.120	0.988	0.849 – 1.150	0.879	1.091	0.902 – 1.320	0.369
	**Primary Care (ctd)**						**Secondary Care**					
	**HbA1c**			**Smoking Status**			**Planned Admissions**			**Unplanned Admissions**		
	RR	95% CI	p-value	RR	95% CI	p-value	RR	95% CI	p-value	RR	95% CI	p-value
3-4	0.950	0.794 – 1.137	0.574	0.947	0.858 – 1.047	0.287	0.753	0.694 – 0.818	<0.001	0.686	0.642 – 0.732	<0.001
5-6	1.038	0.868 – 1.242	0.682	0.945	0.856 – 1.045	0.269	0.787	0.724 – 0.854	<0.001	0.67	0.628 – 0.715	<0.001
7-10	1.031	0.862 – 1.234	0.737	0.885	0.801 – 0.977	0.016	0.812	0.748 – 0.882	<0.001	0.683	0.640 – 0.729	<0.001

Supplementary Table S8: Estimated rate ratios (RRs) from log-linear regression models for each HCU observed pre-pandemic and in the national lockdowns, adjusted for the deprivation group. NL1 = First national lockdown, NL2 = Second national lockdown, NL3 = Third national lockdown, IMD = index of multiple deprivation.

	**Primary Care**											
	**New Prescriptions**			**BP**			**BMI**			**Cholesterol**		
	RR	95% CI	p-value	RR	95% CI	p-value	RR	95% CI	p-value	RR	95% CI	p-value
NL1 vs pre-pandemic	0.668	0.629 – 0.711	**<0.001**	0.133	0.118 – 0.149	**<0.001**	0.161	0.139 – 0.186	**<0.001**	0.125	0.102 – 0.154	**<0.001**
NL2 vs pre-pandemic	0.819	0.763 – 0.879	**<0.001**	0.491	0.430 – 0.560	**<0.001**	0.555	0.469 – 0.656	**<0.001**	0.782	0.615 – 0.994	**0.045**
NL3 vs pre-pandemic	0.837	0.790 – 0.886	**<0.001**	0.520	0.467 – 0.579	**<0.001**	0.599	0.523 – 0.686	**<0.001**	0.824	0.678 – 1.001	0.051
IMD: 3-4 vs IMD: 1-2	0.895	0.846 – 0.947	**<0.001**	1.011	0.910 – 1.123	0.837	0.994	0.871 – 1.135	0.933	0.977	0.808 – 1.181	0.810
IMD: 5-6 vs IMD: 1-2	0.891	0.843 – 0.943	**<0.001**	1.086	0.978 – 1.206	0.122	0.998	0.874 – 1.139	0.971	1.087	0.899 – 1.314	0.389
IMD: 7-10 vs IMD: 1-2	0.846	0.800 – 0.895	**<0.001**	1.099	0.989 – 1.220	0.079	0.977	0.856 – 1.116	0.732	1.128	0.933 – 1.364	0.213
NL1 vs pre-pandemic : IMD: 3-4 vs IMD: 1-2	1.043	0.956 – 1.137	0.342	1.076	0.916 – 1.265	0.372	1.056	0.861 – 1.296	0.600	0.976	0.729 – 1.308	0.871
NL2 vs pre-pandemic : IMD: 3-4 vs IMD: 1-2	1.033	0.935 – 1.143	0.522	1.006	0.834 – 1.214	0.947	1.070	0.845 – 1.357	0.574	0.984	0.701 – 1.382	0.926
NL3 vs pre-pandemic : IMD: 3-4 vs IMD: 1-2	1.021	0.941 – 1.108	0.617	0.981	0.843 – 1.142	0.806	1.021	0.843 – 1.237	0.831	0.978	0.743 – 1.287	0.872
NL1 vs pre-pandemic : IMD: 5-6 vs IMD: 1-2	1.043	0.956 – 1.137	0.342	1.124	0.956 – 1.320	0.158	1.064	0.867 – 1.305	0.553	1.037	0.774 – 1.389	0.810
NL2 vs pre-pandemic : IMD: 5-6 vs IMD: 1-2	1.048	0.948 – 1.159	0.360	1.029	0.853 – 1.241	0.765	1.022	0.806 – 1.295	0.857	1.000	0.712 – 1.404	0.999
NL3 vs pre-pandemic : IMD: 5-6 vs IMD: 1-2	1.025	0.944 – 1.112	0.559	1.002	0.861 – 1.166	0.980	1.025	0.846 – 1.242	0.803	0.960	0.729 – 1.264	0.773
NL1 vs pre-pandemic : IMD: 7-10 vs IMD: 1-2	1.034	0.949 – 1.128	0.444	1.159	0.986 – 1.363	0.073	1.088	0.887 – 1.334	0.419	0.889	0.663 – 1.191	0.430
NL2 vs pre-pandemic : IMD: 7-10 vs IMD: 1-2	1.048	0.948 – 1.159	0.362	1.037	0.860 – 1.251	0.701	1.018	0.803 – 1.290	0.885	1.006	0.716 – 1.413	0.973
NL3 vs pre-pandemic : IMD: 7-10 vs IMD: 1-2	1.019	0.939 – 1.106	0.648	0.969	0.833 – 1.128	0.686	0.971	0.801 – 1.177	0.764	0.923	0.701 – 1.216	0.570

	**Primary Care (ctd)**						**Secondary Care**					
	**HbA1c**			**Smoking Status**			**Planned Admissions**			**Unplanned Admissions**		
	RR	95% CI	p-value	RR	95% CI	p-value	RR	95% CI	p-value	RR	95% CI	p-value
NL1 vs pre-pandemic	0.142	0.115 – 0.175	**<0.001**	0.352	0.309 – 0.401	**<0.001**	**0.566**	**0.516 - 0.621**	**<0.001**	**0.642**	**0.558 - 0.737**	**<0.001**
NL2 vs pre-pandemic	0.794	0.622 – 1.013	0.064	0.734	0.632 – 0.853	**<0.001**	0.916	0.823 - 1.020	0.110	0.944	0.803 - 1.109	0.479
NL3 vs pre-pandemic	0.871	0.714 – 1.061	0.171	0.735	0.651 – 0.831	**<0.001**	**0.894**	**0.820 - 0.976**	**0.012**	0.945	0.829 - 1.077	0.393
IMD: 3-4 vs IMD: 1-2	0.947	0.781 – 1.149	0.582	0.977	0.868 – 1.101	0.707	**0.761**	**0.699 - 0.828**	**<0.001**	0.654	0.576 - 0.743	**<0.001**
IMD: 5-6 vs IMD: 1-2	1.038	0.856 – 1.259	0.705	0.978	0.868 – 1.101	0.713	**0.799**	**0.734 - 0.870**	**<0.001**	**0.598**	**0.527 - 0.680**	**<0.001**
IMD: 7-10 vs IMD: 1-2	1.041	0.858 – 1.263	0.681	0.972	0.863 – 1.094	0.636	**0.848**	**0.779 - 0.923**	**<0.001**	**0.643**	**0.566 - 0.731**	**<0.001**
NL1 vs pre-pandemic : IMD: 3-4 vs IMD: 1-2	1.000	0.743 – 1.347	0.999	0.958	0.798 – 1.150	0.646	0.988	0.867 - 1.126	0.858	1.097	0.902 - 1.335	0.351
NL2 vs pre-pandemic : IMD: 3-4 vs IMD: 1-2	0.991	0.702 – 1.401	0.961	0.997	0.806 – 1.233	0.977	1.001	0.860 - 1.165	0.990	1.017	0.810 - 1.277	0.883
NL3 vs pre-pandemic : IMD: 3-4 vs IMD: 1-2	0.991	0.749 – 1.311	0.949	0.97	0.816 – 1.152	0.725	0.997	0.881 - 1.127	0.959	1.061	0.882 - 1.276	0.525
NL1 vs pre-pandemic : IMD: 5-6 vs IMD: 1-2	1.020	0.758 – 1.374	0.895	0.974	0.811 – 1.170	0.779	0.765	0.671 - 0.872	**<0.001**	1.098	0.902 - 1.336	0.348
NL2 vs pre-pandemic : IMD: 5-6 vs IMD: 1-2	1.009	0.714 – 1.426	0.958	0.933	0.754 – 1.153	0.519	1.043	0.896 - 1.214	0.588	0.981	0.781 - 1.233	0.871
NL3 vs pre-pandemic : IMD: 5-6 vs IMD: 1-2	0.960	0.726 – 1.270	0.776	0.948	0.798 – 1.126	0.541	1.077	0.952 - 1.218	0.235	1.247	1.037 - 1.500	**0.020**
NL1 vs pre-pandemic : IMD: 7-10 vs IMD: 1-2	0.929	0.690 – 1.251	0.629	0.984	0.819 – 1.181	0.861	0.695	0.609 - 0.792	**<0.001**	0.898	0.738 - 1.093	0.280
NL2 vs pre-pandemic : IMD: 7-10 vs IMD: 1-2	1.019	0.721 – 1.440	0.915	0.852	0.689 – 1.054	0.139	1.032	0.887 - 1.202	0.678	1.173	0.934 - 1.473	0.169
NL3 vs pre-pandemic : IMD: 7-10 vs IMD: 1-2	0.927	0.700 – 1.226	0.595	0.836	0.704 – 0.994	**0.042**	1.068	0.944 - 1.208	0.291	1.164	0.968 - 1.401	0.105

Supplementary Table S9: Comparison of HCU between deprivation groups and the highly deprived population throughout the pandemic for multi-morbid patients.

	Deprivation 3-4 vs 1-2		Deprivation 5-6 vs 1-2		Deprivation 7-10 vs 1-2	
	RR (95% CI)	p-value	RR (95% CI)	p-value	RR (95% CI)	p-value
**Primary HCU**						
New prescriptions	0.917 (0.878 – 0.957)	**<0.001**	0.867 (0.830 – 0.905)	**<0.001**	0.807 (0.773 – 0.843)	**<0.001**
BP	0.983 (0.844 – 1.145)	0.827	0.997 (0.856 – 1.161)	0.969	1.001 (0.860 – 1.166)	0.989
BMI	0.927 (0.796 – 1.081)	0.334	0.893 (0.766 – 1.041)	0.147	0.865 (0.742 – 1.008)	0.063
Cholesterol	0.939 (0.779 – 1.133)	0.510	0.969 (0.803 – 1.168)	0.738	0.958 (0.794 – 1.155)	0.652
HbA1c	0.927 (0.779 – 1.104)	0.396	0.942 (0.791 – 1.122)	0.504	0.919 (0.772 – 1.094)	0.342
Smoking status	0.863 (0.785 – 0.949)	**0.003**	0.820 (0.745 – 0.901)	**<0.001**	0.766 (0.697 – 0.843)	**<0.001**
**Secondary HCU**						
Planned admissions	0.873 (0.773 – 0.986)	**0.029**	0.741 (0.652 – 0.842)	**<0.001**	0.705 (0.619 – 0.802)	**<0.001**
Unplanned admissions	0.826 (0.696 – 0.980)	**0.029**	0.669 (0.557 – 0.802)	**<0.001**	0.645 (0.536 – 0.774)	**<0.001**

Supplementary Table S10: Associated interaction between the first national lockdown and deprivation group on primary HCU compared to pre-pandemic rate ratios between deprivation groups, for multi-morbid patients.

	Deprivation 3-4 vs 1-2		Deprivation 5-6 vs 1-2		Deprivation 7-10 vs 1-2	
	RR (95% CI)	p-value	RR (95% CI)	p-value	RR (95% CI)	p-value
**Primary HCU**						
New prescriptions	1.022 (0.913 – 1.145)	0.697	1.012 (0.904 – 1.133)	0.831	0.992 (0.886 – 1.111)	0.888
BP	1.097 (0.886 – 1.358)	0.390	1.127 (0.910 – 1.396)	0.267	1.166 (0.942 – 1.444)	0.156
BMI	1.018 (0.760 – 1.362)	0.905	1.025 (0.766 – 1.372)	0.865	1.102 (0.823 – 1.475)	0.508
Cholesterol	0.958 (0.644 – 1.424)	0.829	1.004 (0.675 – 1.492)	0.986	0.853 (0.574 – 1.269)	0.428
HbA1c	0.988 (0.665 – 1.467)	0.950	0.996 (0.670 – 1.479)	0.983	0.890 (0.599 – 1.323)	0.561
Smoking status	0.939 (0.726 – 1.215)	0.628	0.945 (0.731 – 1.223)	0.665	0.957 (0.739 – 1.238)	0.733
**Secondary HCU**						
Planned admissions	1.011 (0.576 – 1.770)	0.970	0.748 (0.392 – 1.404)	0.371	0.731 (0.387 – 1.359)	0.326
Unplanned admissions	1.153 (0.513 – 2.592)	0.729	1.202 (0.490 – 2.919)	0.685	0.889 (0.346 – 2.217)	0.803

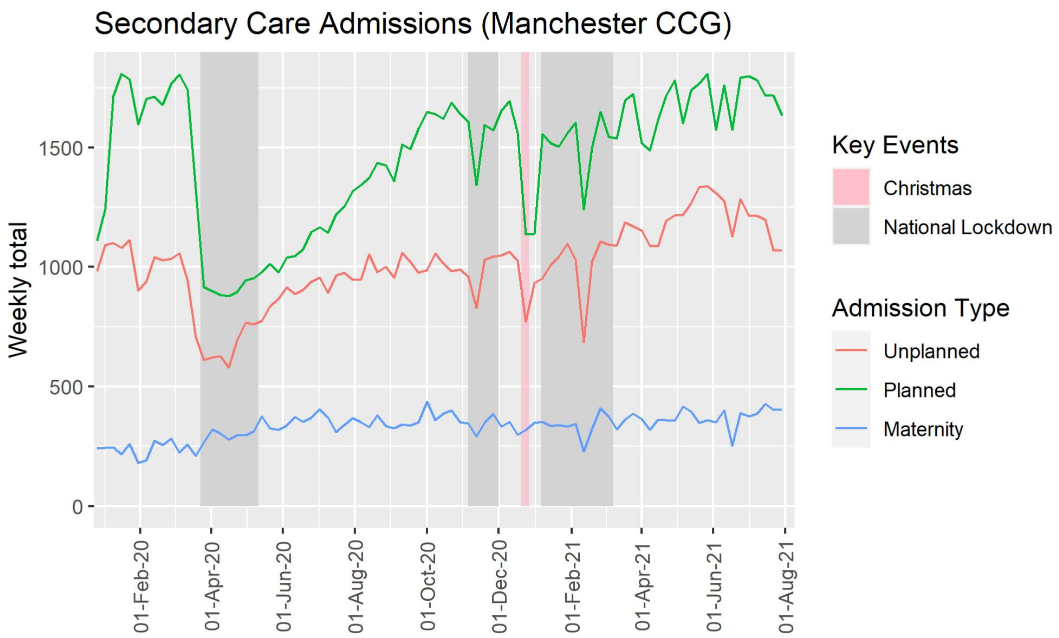

Supplementary Figure S4: Secondary healthcare utilisation across Manchester CCG, from January 2020 until August 2021.

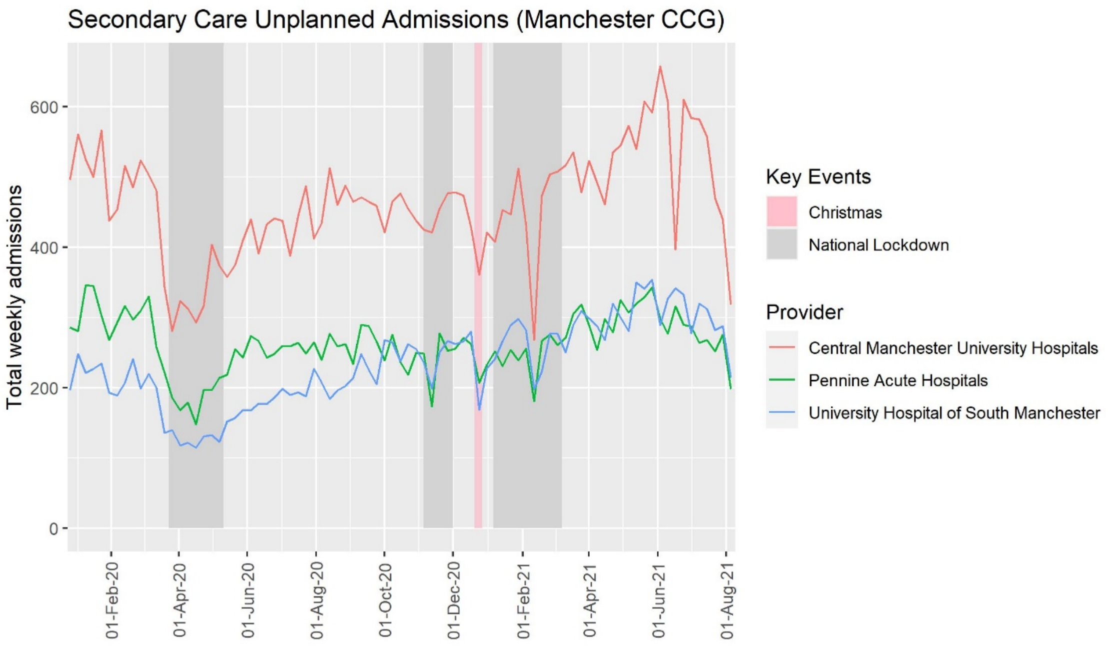

Supplementary Figure S5: Total weekly unplanned admissions of MCCG population at the three main providers (local to 96.6% of people) between January 2020 and August 2021.

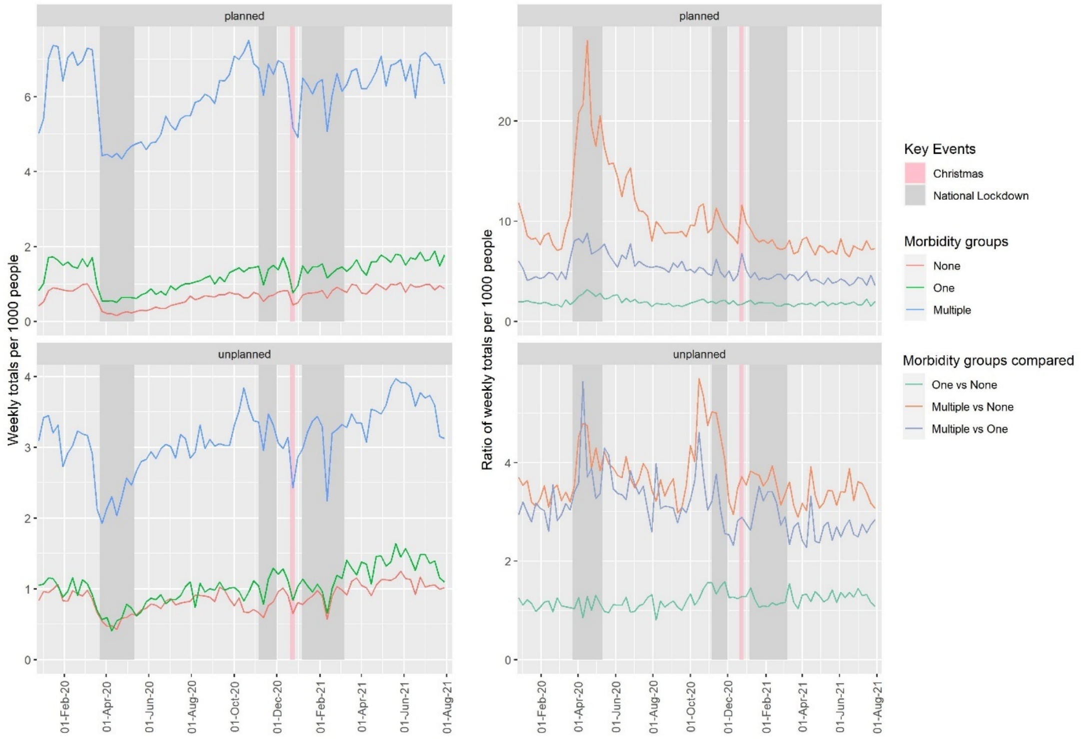

Supplementary Figure S6: Weekly planned and unplanned admissions per 1000 people within morbidity group in the MCCG population and the ratio between these scaled rates, between January 2020 and August 2021.

Supplementary Table S11: Estimates of the ratios of planned and unplanned admission rates for each LTC group in the final four weeks of the study compared to pre-pandemic rates.

	Planned			Unplanned		
LTC	Rate Ratio	95% CI	p-value	Rate Ratio	95% CI	p-value
Cancer	0.653	0.586 – 0.726	**<0.001**	0.926	0.773 – 1.105	0.401
Cardiovascular	0.938	0.901 – 0.977	**0.002**	0.978	0.915 – 1.044	0.502
Endocrine	0.997	0.951 – 1.044	0.893	1.000	0.930 – 1.076	0.990
Gastrointestinal	0.959	0.921 – 0.998	**0.039**	1.104	1.039 – 1.171	**0.001**
Musculoskeletal or Skin	1.025	0.974 – 1.078	0.340	1.093	1.017 – 1.173	**0.014**
Neurological	1.053	0.908 – 1.218	0.489	1.137	0.946 – 1.360	0.165
Psychiatric	1.006	0.959 – 1.054	0.814	1.113	1.049 – 1.181	**<0.001**
Renal or Urological	0.926	0.885 – 0.969	**0.001**	0.839	0.750 – 0.938	**0.002**
Respiratory	0.966	0.910 – 1.025	0.255	1.020	0.947 – 1.098	0.597
Sensory Impairment or Learning Disability	0.894	0.841 – 0.950	**<0.001**	1.016	0.926 – 1.113	0.736
Substance Abuse	1.196	1.074 – 1.329	**0.001**	1.139	1.013 – 1.278	**0.028**

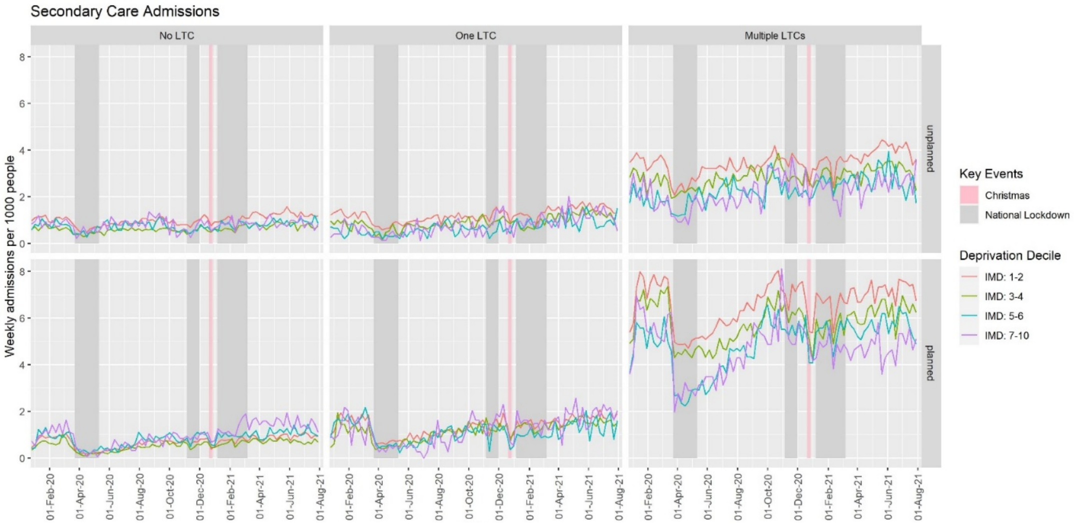

Supplementary Figure S7: Rates of weekly planned and unplanned admissions by deprivation and number of long-term conditions of the Manchester CCG population, between January 2020 and August 2021.

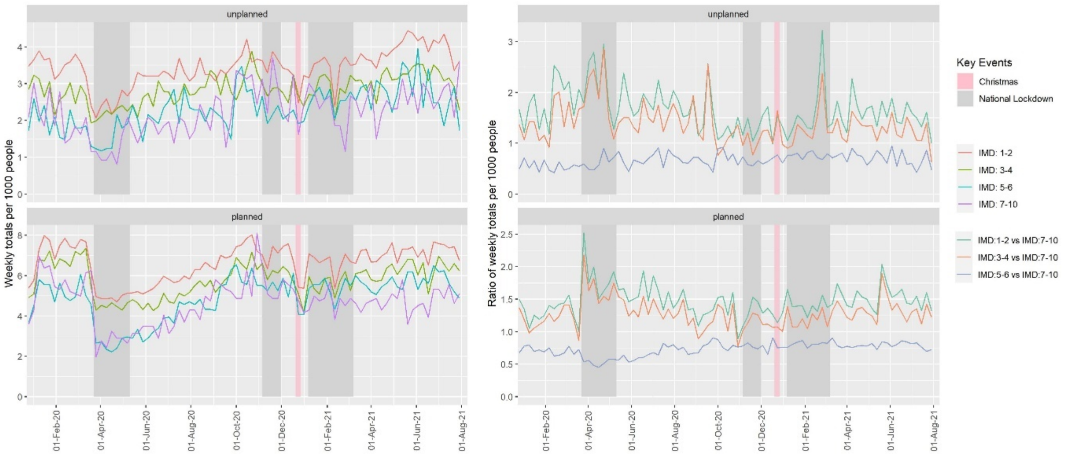

Supplementary Figure S8: Weekly planned and unplanned secondary care admissions per 1000 people within multi-morbid patients by deprivation group and the ratio of these compared to the least-deprived group (IMD: 7-10), between January 2020 and August 2021.

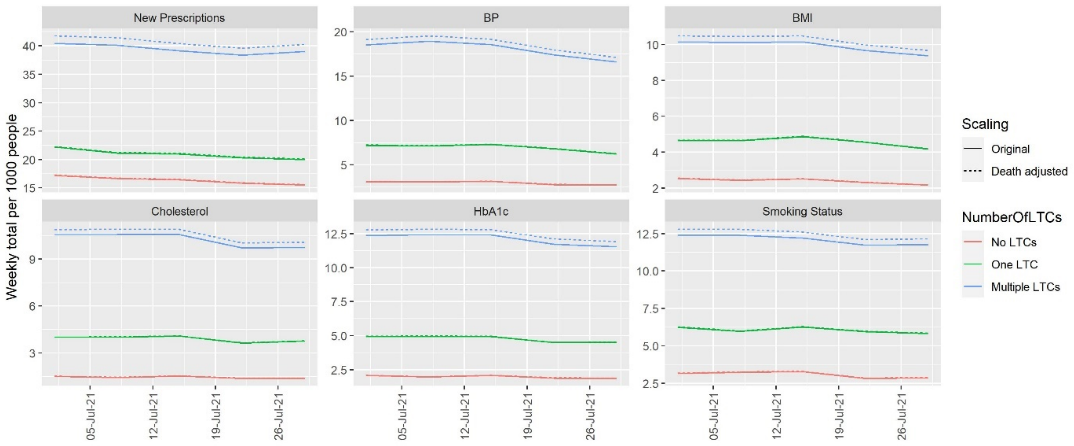

Supplementary Figure S9: Comparison between unadjusted (original) and death-adjusted weekly primary HCU measures per 1000 people, by morbidity in July 2021.

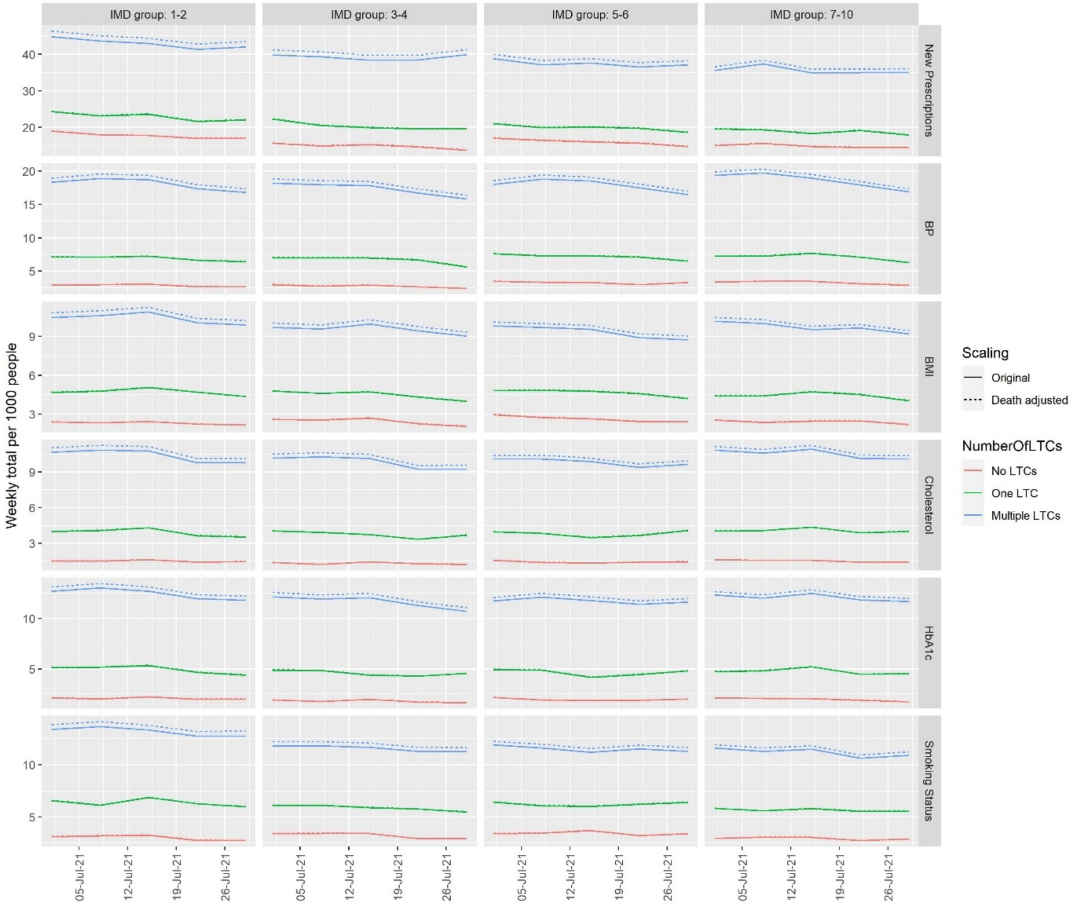

Supplementary Figure 10: Comparison between unadjusted (original) and death-adjusted weekly primary HCU measures per 1000 people, by deprivation group in July 2021.

Supplementary Table S12: Comparison between unadjusted (original) and death-adjusted weekly secondary care admissions per 1000 people, by morbidity group in July 2021.

Number of LTCs (morbidity group)	Admission Type	Week start	Unadjusted Weekly admissions per 1000 people	Death-Adjusted Weekly admissions per 1000 people	Difference
None	unplanned	01/07/2021	1.0235	1.0258	0.0023
None	unplanned	08/07/2021	1.0460	1.0483	0.0024
None	unplanned	15/07/2021	1.0560	1.0584	0.0024
None	unplanned	22/07/2021	0.9934	0.9957	0.0022
None	unplanned	29/07/2021	1.0185	1.0208	0.0023
One	unplanned	01/07/2021	1.4832	1.4898	0.0066
One	unplanned	08/07/2021	1.3576	1.3637	0.0060
One	unplanned	15/07/2021	1.3969	1.4031	0.0062
One	unplanned	22/07/2021	1.1536	1.1587	0.0051
One	unplanned	29/07/2021	1.0987	1.1035	0.0049
Multiple	unplanned	01/07/2021	3.6952	3.8118	0.1166
Multiple	unplanned	08/07/2021	3.7372	3.8551	0.1179
Multiple	unplanned	15/07/2021	3.5932	3.7066	0.1134
Multiple	unplanned	22/07/2021	3.1553	3.2548	0.0995
Multiple	unplanned	29/07/2021	3.1253	3.2239	0.0986
None	planned	01/07/2021	0.9859	0.9882	0.0022
None	planned	08/07/2021	0.9884	0.9907	0.0022
None	planned	15/07/2021	0.8458	0.8477	0.0019
None	planned	22/07/2021	0.9559	0.9581	0.0021
None	planned	29/07/2021	0.8733	0.8753	0.0020
One	planned	01/07/2021	1.6244	1.6317	0.0072

One	planned	08/07/2021	1.6558	1.6632	0.0073
One	planned	15/07/2021	1.8834	1.8918	0.0084
One	planned	22/07/2021	1.4832	1.4898	0.0066
One	planned	29/07/2021	1.7736	1.7814	0.0079
Multiple	planned	01/07/2021	7.1864	7.4131	0.2267
Multiple	planned	08/07/2021	7.0485	7.2708	0.2224
Multiple	planned	15/07/2021	6.8385	7.0542	0.2157
Multiple	planned	22/07/2021	6.8745	7.0914	0.2169
Multiple	planned	29/07/2021	6.3526	6.5530	0.2004
