# Supplementary material for: Healthcare utilisation in patients with long-term conditions during the COVID-19 pandemic: a population-based observational study of all patients across Greater Manchester, UK
Source: BMJ Open. 2023 Jul 7;13(7):e066873. doi: 10.1136/bmjopen-2022-066873 (PMC10335594; doi:10.1136/bmjopen-2022-066873)
Supplement: Author's manuscript [file bmjopen-2022-066873.draft_revisions.pdf]

BMJ Open is committed to open peer review. As part of this commitment we make the peer review history of every article we publish publicly available.

When an article is published we post the peer reviewers' comments and the authors' responses online. We also post the versions of the paper that were used during peer review. These are the versions that the peer review comments apply to.

The versions of the paper that follow are the versions that were submitted during the peer review process. They are not the versions of record or the final published versions. They should not be cited or distributed as the published version of this manuscript.

BMJ Open is an open access journal and the full, final, typeset and author-corrected version of record of the manuscript is available on our site with no access controls, subscription charges or pay-per-view fees (http://bmjopen.bmj.com).

If you have any questions on BMJ Open's open peer review process please email
info.bmjopen@bmj.com

Healthcare Utilisation in Patients with Long-Term Conditions During the COVID-19 Pandemic: A Population Based Study Across Greater Manchester, UK

Journal:	*BMJ Open*
Manuscript ID	bmjopen-2022-066873
Article Type:	Original research
Date Submitted by the Author:	07-Sep-2022

Complete List of Authors:	Sammut-Powell, Camilla; The University of Manchester Faculty of Biology Medicine and Health, Division of Informatics, Imaging and Data Science; The University of Manchester Faculty of Biology Medicine and Health, National Institute for Health Research Applied Research Collaboration Greater Manchester<br>Williams, Richard; The University of Manchester Faculty of Biology Medicine and Health, Division of Informatics, Imaging and Data Science; The University of Manchester Faculty of Biology Medicine and Health, National Institute for Health Research Greater Manchester Patient Safety Translational Research Centre<br>Sperrin, Matthew; The University of Manchester Faculty of Biology Medicine and Health, Division of Informatics, Imaging and Data Science<br>Thomas, Owain; Langworthy Medical Practice<br>Peek, N; The University of Manchester Faculty of Biology Medicine and Health, Division of Informatics, Imaging, and Data Science; The University of Manchester Faculty of Biology Medicine and Health, National Institute for Health Research Applied Research Collaboration Greater Manchester<br>Grant, Stuart; The University of Manchester Division of Cardiovascular Sciences, ERC, Manchester University Hospitals Foundation Trust

Keywords:	COVID-19, Epidemiology < TROPICAL MEDICINE, Public health < INFECTIOUS DISEASES

SCHOLARONE™ Manuscripts

I, the Submitting Author has the right to grant and does grant on behalf of all authors of the Work (as defined in the below author licence), an exclusive licence and/or a non-exclusive licence for contributions from authors who are: i) UK Crown employees; ii) where BMJ has agreed a CC-BY licence shall apply, and/or iii) in accordance with the terms applicable for US Federal Government officers or employees acting as part of their official duties; on a worldwide, perpetual, irrevocable, royalty-free basis to BMJ Publishing Group Ltd ("BMJ") its licensees and where the relevant Journal is co-owned by BMJ to the co-owners of the Journal, to publish the Work in this journal and any other BMJ products and to exploit all rights, as set out in our licence.

The Submitting Author accepts and understands that any supply made under these terms is made by BMJ to the Submitting Author unless you are acting as an employee on behalf of your employer or a postgraduate student of an affiliated institution which is paying any applicable article publishing charge ("APC") for Open Access articles. Where the Submitting Author wishes to make the Work available on an Open Access basis (and intends to pay the relevant APC), the terms of reuse of such Open Access shall be governed by a Creative Commons licence – details of these licences and which Creative Commons licence will apply to this Work are set out in our licence referred to above.

Other than as permitted in any relevant BMJ Author's Self Archiving Policies, I confirm this Work has not been accepted for publication elsewhere, is not being considered for publication elsewhere and does not duplicate material already published. I confirm all authors consent to publication of this Work and authorise the granting of this licence.

For peer review only

Healthcare Utilisation in Patients with Long-Term Conditions During the COVID-19 Pandemic: A Population Based Study Across Greater Manchester, UK

Camilla Sammut-Powell[1,2], Richard Williams[1,2,3], Matthew Sperrin[1], Owain Thomas[4], Niels Peek [1,2,3,5], Stuart W. Grant[6]

1. Division of Informatics, Imaging and Data Science, Faculty of Biology, Medicine and Health, University of Manchester, Manchester, M13 9PL, UK
2. National Institute for Health Research Applied Research Collaboration Greater Manchester, Faculty of Biology, Medicine and Health, University of Manchester, Manchester, M13 9PL, UK
3. National Institute for Health Research Greater Manchester Patient Safety Translational Research Centre, Faculty of Biology, Medicine and Health, University of Manchester, Manchester, M13 9PL, UK
4. Langworthy Medical Practice, Salford, M6 5WW, UK
5. National Institute for Health Research Manchester Biomedical Research Centre, Faculty of Biology, Medicine and Health, University of Manchester, Manchester, M13 9PL, UK
6. Division of Cardiovascular Sciences, University of Manchester, ERC, Manchester University Hospitals Foundation Trust, Manchester, M23 9LT, UK.

Corresponding Author: Stuart W Grant, Division of Cardiovascular Sciences, University of Manchester, ERC, Manchester University Hospitals Foundation Trust, Manchester, M23 9LT, UK.

Corresponding Author's Email: stuart.grant@manchester.ac.uk

For peer review only

ABSTRACT

Objectives: Data on population healthcare utilisation (HCU) across both primary and secondary care during the COVID-19 pandemic are lacking. We describe primary and secondary HCU stratified by long-term conditions (LTCs) and deprivation, during the first 19 months of COVID-19 pandemic across a large urban area in the United Kingdom.

Design: A retrospective, observational study.

Setting: All primary and secondary care that contributed to the Greater Manchester Care Record throughout 30th-December-2019 to 1st-August-2021.

Participants: 3,225,169 patients were registered with or attended an NHS primary or secondary service in Greater Manchester.

Primary outcomes: Primary care HCU (incident prescribing and recording of healthcare information) and secondary care HCU (planned and unplanned admissions) were assessed.

Results: The first national lockdown was associated with reductions in all primary HCU measures, ranging from 24.7% (24.0% to 25.5%) for incident prescribing to 84.9% (84.2% to 85.5%) for cholesterol monitoring. Secondary HCU also dropped significantly for planned (47% (42.9% to 51.5%)) and unplanned admissions (35.0% (28.3% to 41.6%)). Only secondary care had significant reductions in HCU during the second national lockdown. Primary HCU measures had not recovered to pre-pandemic levels by the end of the study. The secondary admission rate ratio between multi-morbid patients and those without LTCs increased during the first lockdown by a factor of 2.4 (2.0 to 2.9;p<0.001) for planned admissions and 1.3 (1.1 to 1.5;p=0.006) for unplanned admissions. No significant changes in this ratio were observed in primary HCU.

Conclusion: Major changes in primary and secondary HCU have been observed during the COVID-19 pandemic. Secondary HCU reduced more in those without LTCs and the ratio of utilisation between the most and least deprived increased for the majority of HCU measures. Overall primary HCU measures and secondary care HCU for some LTC groups had not returned to pre-pandemic levels by the end of the study.

STRENGTHS AND LIMITATIONS OF THIS STUDY

Strengths

- This study includes data on over 3 million individuals, representing all patients registered with a general practitioner across an entire geographical area
- Health care utilisation from both primary and secondary care across the spectrum of long term conditions during the COVID-19 pandemic was analysed
- The effects of social deprivation and its interaction with morbidity on healthcare utilisation during the pandemic were estimated

Limitations

For peer review only - http://bmjopen.bmj.com/site/about/guidelines.xhtml

- Historical data prior to the start of the COVID-19 pandemic were not available limiting the trend analyses that could be performed
- Data from secondary care providers was limited to only a subset of the population

INTRODUCTION

On the 30th of January 2020, the World Health Organization (WHO) declared a public health emergency of international concern with governments urged to prepare for global spread of COVID-19.[1] With case numbers increasing and the virus spreading globally, COVID-19 was characterised as a pandemic six weeks later and rapidly developed into a global public health emergency. As of the 17th of December 2021 approximately 273 million cases and 5.3 million COVID-19 associated deaths have been reported globally.[2]Governments across the world enacted a range of measures aimed at controlling the spread of the virus,[3] and increasing healthcare capacity.[4,5] Despite these measures healthcare systems have been overwhelmed and diversion of healthcare resources to address increased demand specific to COVID-19 has been required.[6,7] The impact of this diversion of resources on the care of patients with non COVID-19 illnesses has been exacerbated by reduced staff availability due to COVID-19 infection amongst healthcare workers.[8]

Numerous studies have been undertaken to assess the impact of the pandemic on healthcare provision in a variety of settings. An analysis of UK general practitioner (GP) data demonstrated that diagnoses of common physical and mental health conditions decreased substantially early in the pandemic.[9] The number of urgent GP referrals for cancer fell by 60% in April 2020 compared to the same month in 2019.[10] Hospital administrative data has demonstrated a decline in patients presenting with acute coronary syndrome from mid-February 2020 onwards, [11] and a separate analysis demonstrated a 43% reduction in patients undergoing percutaneous coronary interventions for ST-elevation myocardial infarctions compared to previous years.[12] Modelling studies have suggested that approximately 28,000,000 elective surgical procedures were cancelled over a 12-week period of peak disruption caused by the pandemic.[13]

Most studies to date investigating the impact of the pandemic on healthcare utilisation (HCU) have assessed specific patient groups, largely focussed on secondary care.[14] Changes to HCU during the COVID-19 pandemic for both primary and secondary care stratified across the range of long-term medical conditions (LTCs) and different levels of social deprivation have not previously been described. The Greater Manchester Care Record (GMCR) includes electronic health records from all primary and secondary care National Health Service (NHS) providers in the metropolitan county of Greater Manchester (GM). GM has been significantly affected by COVID-19,[15] and the GMCR provides a unique opportunity to study the impact of the pandemic on primary and secondary HCU in patients with LTCs across this defined urban area.

METHODS

Design and Data Source

This was a retrospective, observational, service evaluation using routinely collected data. The data analysed were from two sources: 1) HCU data from the GMCR which is an integrated patient record containing data from primary and secondary NHS services across Greater Manchester (GM) and 2) contextual Government COVID-19 data [16] regarding the number of new COVID-19 cases and COVID-19 related hospital admissions.

Greater Manchester Care Record

For peer review only - http://bmjopen.bmj.com/site/about/guidelines.xhtml

The GMCR is populated with data from primary care (General Practitioners), secondary care (acute and community hospitals), mental health trusts and social care across an entire geographical region. A total of nine secondary care organisations (including 12 hospitals), 3 mental health trusts and 10 clinical commissioning groups (CCGs) contribute data. The primary purpose of the GMCR is for direct patient care as it provides clinicians with information from other health care providers relevant to their patient encounters that would ordinarily be inaccessible. However, it has also been made available in de-identified format for research relating to COVID-19.

UK Government COVID-19 Data

Data regarding the number of new COVID-19 cases and COVID-19 related hospital admissions were collected by the UK government throughout the pandemic. The number of new cases by specimen date was extracted for Manchester and the number of COVID-19 admissions were extracted for each of the secondary acute providers serving the people with a Manchester CCG (MCCG), included within the GMCR. The data is freely available from https://coronavirus.data.gov.uk/details/download and full details of the data extraction are provided in the Supplementary Materials (Supplementary Table S1).

Data processing and approvals

All identifiable data including free text are redacted. Some non-identifying demographic data are available such as recorded gender, year of birth, lower layer super output area (LSOA), index of multiple deprivation and ethnicity. The University of Manchester is permitted to perform research on this data via a Greater Manchester wide data protection impact assessment (DPIA). The basis for this DPIA is the control of patient information (COPI) notice issued by the Secretary of State for Health and Social Care in March 2020 which allowed confidential patient information to be shared for the purposes of research into COVID-19.[17] The study was approved by both the GMCR Expert Review Group and Research Governance Group. All data made available to the analysts was de-identified and aggregated and therefore did not require specific ethical approval.

Study populations and key time points

The main study population consisted of all patients that were registered with a GP within GM on 1st January 2020, defined as the GM population. The 1st January 2020 is the index study date. For the primary care analyses the entire GM population were considered. However, secondary care data were only available for patients registered to a Manchester CCG, hence, the secondary healthcare utilisation analyses were limited to these people. The dates of the national lockdowns initiated in response to the COVID-19 pandemic were indicated in addition to Christmas week due to expected changes in HCU during these periods. The first national lockdown ran from the 23rd March 2020 to the 11th May 2020, the second national lockdown ran from 5th November 2020 to 1st December 2020 and the third national lockdown ran from 6th January 2021 to the 8th March 2021.

Long-term medical conditions

Long-term medical conditions (LTCs) were defined as per Barnett et al,[18] and were grouped into the following categories: cancer, cardiovascular, endocrine, gastrointestinal, musculoskeletal or skin, neurological, psychiatric, renal or urological, respiratory, sensory impairment or learning disability, and substance abuse. A resident was identified as being diagnosed with a LTC by interrogating the GMCR record prior to the index date. If a long-term medical condition was diagnosed after the 1st

For peer review only - http://bmjopen.bmj.com/site/about/guidelines.xhtml

January 2020 the patient was not recorded as having the LTC for this analysis. People that were identified as belonging to multiple LTC groups were assigned to each corresponding LTC group and defined as multi-morbid. The full list of long-term conditions and groupings are provided in Supplementary Table S2.

Index of Multiple Deprivation

The 2019 Index of Multiple Deprivation (IMD) is the official measure of relative deprivation provided by the Office for National Statistics which combines information from seven different domains to produce an overall relative measure of deprivation for each LSOA. Each LSOA is ranked from least to most deprived, and deciles of relative deprivation are generated.[19] For this study the available IMD deciles were categorised into four groups, representing the most deprived (deciles 1-2), highly deprived (deciles 3-4), moderately deprived (deciles 5-6) and the least deprived LSOAs (deciles 7-10). The least deprived group consisted of 4 deciles to avoid multiple small groups because of the skew towards more deprived deciles in GM.

Measuring Healthcare Utilisation

For primary HCU, surrogate markers were evaluated consisting of first prescriptions and recording of healthcare information in the GP record. First prescriptions were identified by the issuing of any new prescription for an individual patient by a primary care healthcare professional and are referred to as incident prescriptions throughout the manuscript. This measure was selected as issuing of an incident prescription requires contact with a healthcare professional. Healthcare information recorded included: recoding of smoking status, measurements of cholesterol, blood pressure (BP), blood glucose (HbA1c) and body mass index (BMI). The values of these measurements were not used in the analysis.

For secondary HCU, the number of acute provider admissions were evaluated. To enable population admission rates to be calculated, a denominator was calculated by assigning each resident within Manchester CCG to a secondary care provider according to the most common provider observed within their LSOA. In cases where there the most common secondary provider was unclear one of the two most common providers was randomly assigned to that LSOA. Secondary care admissions were categorised into planned, unplanned, maternity, transfers and 'other' admission, defined according to the admission type field available in the provider data. Daily aggregate level data counts of all utilisation measures were provided. A full description of the data processing applied is available at https://github.com/rw251/gm-idcr/tree/master/projects/001%20-%20Grant.

Statistical Analysis

Weekly totals of HCU data were evaluated for the entire population. The rate ratios (RRs) of utilisation in the weeks before and after the initiation of the first and second national lockdowns (1st national lockdown: w/c 23rd March 2020 vs w/c 9th March 2020; 2nd national lockdown: w/c 9th November 2020 vs w/c 19th October 2020) were estimated across all measures of HCU to determine the association between the initiation of each national lockdown and HCU.

The effect of the initiation of the third national lockdown was not estimated since the weeks prior coincided with Christmas, where utilisation is expected to be reduced. The pre-pandemic weeks (prior to 9th March 2020) were compared against each of the national lockdown periods to determine if there was a significant change in the rates of HCU associated with each of the national lockdowns using Poisson regression. A Poisson regression model, linear in time, was fit to the weekly

For peer review only - http://bmjopen.bmj.com/site/about/guidelines.xhtml

rates of utilisation after the initiation of the first national lockdown until the end of the study to determine the overall change of utilisation throughout the pandemic. A direct comparison between utilisation observed in a calendar week in 2021 vs 2020 was conducted for calendar weeks 2 to 11, using rate ratios; it was assumed that the data in calendar weeks 2 to 11 in 2020 were unaffected by the pandemic and consequently act as a control. Additionally, the rate ratios between utilisation measures in the final four weeks of the study and the pre-pandemic period were estimated using Poisson regression to compare how utilisation differed from pre-pandemic levels by the end of the study.

Subgroup analyses were performed across LTC and IMD groups. To compare subgroups, we further provided the rates of utilisation per 1000 people by dividing by the total number of people assigned to the corresponding subgroup and multiplying by 1000. For example, when comparing the rates across number of LTCs (none, one or multiple), for the people without any LTCs, the rate of weekly secondary care admissions is defined as the total number of admissions experienced by this subgroup within a given week divided by the total number of people within the subgroup, multiplied by 1000. The interactive effect between the each of the national lockdowns and subgroup HCU was estimated using log-linear regression.

A sensitivity analysis to adjust for deaths that occurred during the study was performed, where rates of utilisation were re-calculated in July 2021 dividing by the total numbers of patients that were still alive (death-adjusted), and compared to the un-adjusted rates. July 2021 was chosen since this was one month before the end of the study and therefore captured the majority of deaths that occurred throughout the study; hence if no difference was observed between the death-adjusted and un-adjusted rates, the un-adjusted rates would pertain across all weeks. All analyses were performed in R version 4.0.0, using the packages 'tidyverse'[20], 'scales'[21], 'reshape2'[22] and 'cowplot'[23].

Patient and public involvement

Two public representatives provided input throughout the project. Both representatives gave their full support to the proposed project and are preparing a patient and public summary of the research for dissemination.

RESULTS

The total population captured within the GMCR includes 3,225,169 patients, of whom 693,749 were registered with a Manchester CCG. The prevalence of LTCs is shown in Table 1. The most common LTCs observed were psychiatric (GMCR: 26.5%, MCCG: 20.7%), cardiovascular (GMCR: 17.4%, MCCG: 10.5%), respiratory (GMCR: 17.1%, MCCG: 13.4%) and gastrointestinal (GMCR: 15.5%, MCCG: 11.8%). Levels of deprivation were high, with 41.4% of the GM population and the majority of those registered within Manchester CCG (58.5%) in the most deprived quintile (decile of 1 or 2; Table 1).

Overall primary HCU

There was a rapid decrease in all weekly primary HCU measures starting just prior to the first national lockdown (Figure 1). The largest drops in activity associated with the initiation of the first national lockdown were for recording of healthcare information (% drop (95% CI): BP: 82.4% (82.0%-82.9%); BMI: 79.5% (78.8%-80.1%); Cholesterol 84.9% (84.2%-85.5%); HbA1c 84.0% (83.3%-84.6%); Smoking status 62.2% (61.3%-63.1%). There was still a significant drop in the new prescriptions but the change

For peer review only - http://bmjopen.bmj.com/site/about/guidelines.xhtml

was proportionally smaller (24.7%; 95% CI: 24.0%-25.5%). These reductions were sustained throughout the first national lockdown (Supplementary Table S3). The initiation of the second national lockdown was associated with an increase or no significant change in primary HCU (% increase (95%CI): new prescriptions: -0.3% (-1.3%-0.6%); BP: 4.1% (2.2%-6.1%); BMI: 2.4% (0.0%-4.8%); Cholesterol: 10.0% (7.3%-12.8%); HbA1c: 6.5% (4.1%-8.9%); Smoking status: -0.2% (-2.1%-1.8%)).

Table 1: Long-term conditions and social deprivation identified in the GM population and the Manchester CCG subpopulation.

	Greater Manchester (N=3,225,169)	%	Manchester CCG (N=693,749)	%
**LTC Group***				
Cancer	50954	1.6	6307	0.9
Cardiovascular	561195	17.4	72912	10.5
Endocrine	393274	12.2	64557	9.3
Gastrointestinal	501060	15.5	81957	11.8
Musculoskeletal or Skin	284103	8.8	52593	7.6
Neurological	46672	1.4	7340	1.1
Psychiatric	854454	26.5	143707	20.7
Renal or Urological	130436	4.0	16617	2.4
Respiratory	550648	17.1	93174	13.4
Sensory Impairment or Learning Disability	314264	9.7	45909	6.6
Substance Abuse	115532	3.6	24068	3.5
**Number of LTCs**				
None	1530501	47.5	399618	57.6
One	631648	19.6	127428	18.4
Multiple	1063020	33.0	166703	24.0
**IMD Group**				
1 to 2	1335061	41.4	405862	58.5
3 to 4	675296	20.9	185118	26.7
5 to 6	424139	13.2	67192	9.7
7 to 10	788553	24.4	35087	5.1
Missing	2120	0.1	490	0.1

LTC – long term condition; IMD – index of multiple deprivation. *These data represent the overall prevalence of each long term condition in the population. Each individual can be represented in more than one LTC row.

All primary care HCU moderately increased weekly from the initiation of the first national lockdown to the end of the study (RR (95%CI): new prescriptions: 1.00 (1.00 - 1.00; p<0.001); BP: 1.01 (1.01 – 1.01;p<0.001); BMI: 1.01 (1.01 – 1.01;p<0.001); Cholesterol: 1.02 (1.02 – 1.02;p<0.001); HbA1c: 1.01 (1.01 – 1.02;p<0.001); Smoking status: 1.01 (1.01 – 1.01;p<0.001)). Despite this, by the end of the study, all measures were still recorded less often than in the pre-pandemic period (RR (95% CI; p-value); new prescriptions: 0.828 (0.825 – 0.832; p<0.001); BP: 0.583 (0.580-0.587; p<0.001); BMI: 0.669 (0.664-0.675; p<0.001); cholesterol: 0.896 (0.888-0.905; p<0.001); HbA1c: 0.935 (0.927-0.943; p<0.001); smoking status: 0.711 (0.706-0.717; p<0.001)).

For peer review only

All primary care measures were lower across calendar weeks 2-11 when comparing 2021 data with 2020 data (p<0.001; Supplementary Table S4). The measuring of BP and BMI remained consistently lower throughout these weeks by an average of 50.0% (95%CI: 49.8%-50.2%; p<0.001) and 42.5% (95% CI: 42.1%-42.8%; p<0.001), respectively. Even though the rates of cholesterol and HbA1c measurements taken in calendar week 11 were similar in 2021 (pandemic) and 2020 (pre-pandemic): 0.937 (95% CI: 0.916 – 0.958) and 0.973 (95% CI: 0.953 – 0.992), respectively, they were still significantly lower in 2021.

Primary HCU by multi-morbidity and deprivation

Multi-morbid patients and patients with one LTC had consistently higher levels of primary HCU than patients with no LTCs throughout the study period (Supplementary Table 3a; Figure 2). The ratio of weekly HCU rates per 1000 people between multi-morbid patients and those with no LTCs significantly increased for new prescriptions (RR: 1.281; 95%CI: 1.169 – 1.404; p <0.001), BP (RR: 1.187; 95%CI: 1.007 – 1.400; p = 0.042) and smoking status (RR: 1.356; 95%CI: 1.126 – 1.632; p = 0.002) during the first national lockdown, and decreased for BMI (RR: 0.736; 95%CI 0.613 – 0.885; p=0.001) and smoking status (RR: 0.803; 95%CI 0.675 – 0.956; p=0.014) in the third national lockdown. No significant changes in HCU between multi-morbid patients and those with no LTCs were observed for HBA1c or cholesterol or during the second national lockdown for all primary care HCU (Supplementary Table S6; Supplementary Figure S1).

Primary HCU by deprivation

People that were less deprived had lower rates of new prescriptions compared to the most deprived group (IMD 1-2), (RR (95%CI); 3-4 vs 1-2: 0.915 (0.874 – 0.959); 5-6 vs 1-2: 0.920 (0.878 – 0.964); 7-10 vs 1-2: 0.875 (0.835 – 0.917); Figure 2, Supplementary Table S7). Similarly, smoking status had a lower rate of measurement in the least deprived patients compared to the most deprived patients (RR: 0.885; 95% CI: 0.801 – 0.877). No other differences were observed with regards to deprivation across primary HCU. The least deprived group had experienced an additional reduction in smoking status during the third national lockdown (RR: 0.836; 95%CI 0.704 - 0.994; p=0.042) but no other interactions between deprivation and national lockdowns were evident (Supplementary Table S9).

Interaction between multi-morbidity and deprivation for primary HCU

Differences in HCU by deprivation were overall larger within multi-morbid patients (Supplementary Table S8; Figure 2). Differences in HCU between deprivation groups were not attributable to only one LTC group (Supplementary Figure S2). In multi-morbid patients, there were no significant changes in the ratio of weekly HCU per 1000 people between the least deprived group and the other deprivation groups across all primary HCU measures during the first national lockdown compared to pre-pandemic weeks (Supplementary Table S10; Supplementary Figure S3).

Overall secondary HCU

There has been large variation in planned and unplanned secondary HCU over the course of the COVID-19 pandemic (Supplementary Figure S4). There was a 47.4% (95%CI: 42.9% - 51.5%, p<0.001)

For peer review only - http://bmjopen.bmj.com/site/about/guidelines.xhtml

reduction in planned and 35.3% (95%CI: 28.3% - 41.6%; p<0.001) reduction in unplanned weekly admission rates per 1000 people associated with the initiation of the first national lockdown (planned: 2.51 to 1.32; unplanned: 1.36 to 0.88). The initiation of the second national lockdown was also associated with a significant reduction in secondary HCU; planned weekly admission rates per 1000 people reduced by 20.4% (95%CI 14.4%-25.9%; p<0.001) and unplanned reduced by 15.6% (95%CI 7.3%-23.1%; p<0.001). The reductions were sustained throughout these lockdowns; only unplanned admissions in the third national lockdown were not significantly lower than in pre-pandemic rates (Supplementary Table S3). The patterns observed in secondary admissions were consistent across all three main contributing secondary care providers (Supplementary Figure S5).

Both planned and unplanned weekly admissions were on average lower from the beginning of the first national lockdown up until the end of the study period, compared to the pre-pandemic admissions (planned: RR 0.850, 95%CI 0.837 – 0.864, p<0.001; unplanned: RR 0.976, 95%CI 0.957 – 0.996, p=0.016). However, the admissions increased throughout the period (planned: p<0.001; unplanned: p<0.001) and when comparing the final four weeks of the study period with the pre-pandemic period, planned admission rates were not significantly different (RR: 1.105; 95% CI 0.987 – 1.044; p=0.290) and unplanned were higher (RR: 1.104, 95% CI 1.067 – 1.143; p<0.001). The direct comparison between calendar weeks 2-11 in 2021 vs 2020, indicated that planned admissions were lower on average by 11.3% (95% CI: 9.4% - 13.2%; p<0.001) but there was no difference in unplanned admissions (RR: 1.012; 95%CI 0.985 – 1.040; p=0.376). A week-by-week comparison is detailed in Supplementary Table S3.

Secondary HCU by multi-morbidity

Morbidity was associated with an increased rate of planned admissions throughout the study period: One vs No LTCs RR 1.904 (95% CI: 1.717 – 2.111; p<0.001); Multiple vs No LTCs RR 9.584 (95%CI 8.644 – 10.627; p<0.001); Multiple vs One LTC RR 5.033 (95%CI 4.540 – 5.581; p<0.001). This was also the case for unplanned admissions: One vs No LTCs RR 1.188 (95% CI: 1.112 – 1.270; p<0.001); Multiple vs No LTCs RR 3.636 (95%CI 3.401 – 3.887; p<0.001); Multiple vs One LTC RR 3.059 (95%CI 2.862 – 3.271; p<0.001) (Figure 3).

Whilst the ratio of weekly unplanned admissions per 1000 people between patients that were multi-morbid vs those without any LTCs was consistent throughout the majority of the pandemic, there was a significant increase during the first lockdown compared with pre-pandemic (RR 1.253, 95%CI 1.068 – 1.469; p=0.006) but no significant change was observed between those with one LTC and those without any LTCs (RR 0.987, 95%CI 0.842 – 1.157; p=0.865, Supplementary Figure S6). The ratio of planned admission rates per 1000 people in morbidity groups increased during the first national lockdown compared to that observed pre-pandemic: multi-morbid vs no LTC increased by a factor of 2.402 (95% CI 2.047 – 2.818; p<0.001) and one LTC vs no LTCs increased by a factor of 1.413 (95% CI 1.205 – 1.658; p<0.001) (Supplementary Table S5; Supplementary Figure S6), however this was not sustained throughout the pandemic. The average of the ratios of admission rates between multi-morbid patients vs patients without LTCs from the start of the first national lockdown until the end of the study period vs pre-pandemic was 1.176 (95% CI: 0.871 – 1.588; p =0.289) for planned admission rates and 1.097 (95%CI: 0.900 – 1.338; p=0.357) for unplanned admission rates.

For peer review only - http://bmjopen.bmj.com/site/about/guidelines.xhtml

Secondary care HCU for specific LTC groups

There were noticeable differences for both planned and unplanned admission rates within each LTC group over the study period (Figure 4). Planned admission rates were highest for patients with a renal or urological LTC. Unplanned admission rates were highest in patients with cancer or renal LTCs. Planned and unplanned admission rates were lowest overall for patients without any LTC. For cancer patients, the drop in the number of planned admissions at the initiation of the first national lockdown was sustained throughout the remainder of the study period, with an average reduction of 28.9% (95% CI: 25.1% - 32.5%; p<0.001) compared to pre-pandemic levels and unplanned admission rates decreased by 11.5% (95% CI 2.4% - 19.6%; p=0.014). Planned admission rates for people identified as having an endocrine, musculoskeletal or skin, neurological, psychiatric, or respiratory LTC returned to pre-pandemic levels by the end of the study period. However, planned admission rates for people identified as having cancer, cardiovascular, gastrointestinal, renal or urological and sensory impairment or learning disability remained lower than in the pre-pandemic period. Conversely, planned admission rates for people identified with a substance abuse LTC were higher by the end of the study period compared to the pre-pandemic period (RR: 1.196; 95%CI 1.07 – 1.329; p=0.001; Supplementary Table S11). Unplanned admissions rates were lower only for those that were identified with a renal or urological LTC. Patient groups with a gastrointestinal, musculoskeletal or skin, psychiatric or substance abuse LTC had higher rates of unplanned admissions at the end of the study compared to pre-pandemic levels. The remaining LTC groups had no significant change in unplanned admissions (Supplementary Table S11).

Secondary HCU by deprivation

People that were most deprived (IMD of 1 or 2) had the highest rates for both planned (RR (95%CI); 3-4 vs 1-2: 0.753 (0.694 – 0.818; p<0.001); 5-6 vs 1-2: 0.787 (0.724 – 0.854;p<0.001); 7-10 vs 1-2: 0.812 (0.748 – 0.882; p<0.001)) and unplanned admissions (RR (95%CI); 3-4 vs 1-2: 0.686 (0.642 – 0.732; p<0.001); 5-6 vs 1-2: 0.670 (0.628 – 0.715; p<0.001); 7-10 vs 1-2: 0.683 (0.640 – 0.729; p<0.001)) throughout the study period. For multi-morbid patients, being highly deprived was associated with an increased rate in both planned and unplanned admissions compared to all other deprivation groups (Supplementary Table S3c; Supplementary Figure S7). The ratios of rates between deprivation groups within multi-morbid patients were not significantly different during the first national lockdown compared to pre-pandemic levels (Supplementary Table S10, Supplementary Figure S8).

DISCUSSION

Principle Findings and Interpretation

We have assessed primary HCU for over 3 million patients across GM and secondary HCU for a sub-group of almost 700,000 patients within Manchester CCG. Major changes in HCU occurred during the COVID-19 pandemic. There was a large reduction in both primary and secondary HCU at the beginning of the first national lockdown. Whilst there was a relatively consistent increase in primary care HCU from the first national lockdown, primary HCU remained was lower at the end of the study compared to pre-pandemic. Overall, both planned and unplanned secondary admissions had recovered to pre-pandemic levels by the end of the study period but this recovery was not observed across all LTC

For peer review only - http://bmjopen.bmj.com/site/about/guidelines.xhtml

subgroups. Changes in the ratio of HCU between multi-morbid patients and those without LTCs occurred during national lockdowns but were inconsistent across primary HCU measures.

Although some healthcare information measures can be completed remotely (e.g. smoking status, BP and BMI), primary HCU measures that require in-person contact with a healthcare professional (e.g. HbA1c and cholesterol) demonstrated similar patterns in HCU. The initial larger fall in healthcare information recording compared to incident prescribing in primary care may reflect a shift in focus away from secondary prevention during the first wave of the pandemic. Although these HCU measures have not returned to pre-pandemic levels, they have consistently increased since the first lockdown and this has occurred even though quality outcome framework targets and local enhanced services were largely suspended.

Despite a peak in COVID-19 admissions within the first national lockdown, secondary admissions fell by a larger volume. Reductions in secondary care admissions associated with the first lockdown have been reported across the UK.[11,24,25] Largely, these are reflective of cancellations of elective activity or delaying non-urgent care, to ensure capacity for patients with severe COVID-19 infection and to increase critical care capacity.[7] The observed deficit may not correspond entirely to an unmet need of patients with non-COVID-19 healthcare needs as there is some evidence that changes in behaviour according to sanitisation campaigns, social distancing and government restrictions may have resulted in fewer infections,[26] and injuries.[27] Additionally, emergency department attendances which are related to unplanned admissions (but were not directly assessed in this study) have been observed to have reduced.[14] It is also possible that the increased utilisation of remote management for secondary care patients has contributed to clinically appropriate reductions in admissions.

There has been no noticeable recovery in HCU for patients with cancer and for a number of other LTCs, recovery to pre-pandemic HCU levels has not occurred. In contrast, HCU of patients identified with substance abuse and/or a psychiatric condition exceeded pre-pandemic levels between the first and second national lockdowns, likely reaffirming the significant impact of the pandemic on mental health and psychiatric services.[28]

Implications for Clinicians and Policy Makers

It is inevitable that the changes in HCU observed in this study will have had an impact on both patients and healthcare providers above and beyond the direct impact of COVID-19. For patients with cancer, services had to adapt to mitigate the increased risk of death from COVID-19.[29] The initial reduction in the number of planned admissions was sustained throughout the study period and is likely to reflect changes in services but may also be due to patients with cancer being reluctant to seek healthcare. Delays in care for patients with cancer are known to impact prognosis,[30] and the pandemic has been found to have contributed to excess deaths in patients with cancer.[24] A proactive approach to encourage patients to attend screening and routine appointments will be needed to minimise the impact of the pandemic on patients with cancer and other emerging health inequalities.[31,32] Understanding the implications of reductions in the selected primary care HCU measures, particularly the decrease in assessing and recording healthcare information will require further long-term studies.

For peer review only

Strengths & Limitations

The strengths of this study include the complete coverage of a large geographical area for the primary care analyses and the inclusion of both primary and secondary HCU data. This is the first study to evaluate HCU across the full spectrum of LTC subpopulations and stratify according to multi-morbidity and deprivation. Data prior to 2020 were not available and consequently comparisons made (pre- vs post-pandemic) are reliant on the data between January and March 2020 being representative of pre-pandemic utilisation. Consequently, the comparison of pre-pandemic HCU to the end of the study period may have been influenced by seasonal variations in HCU. The secondary care analysis was only possible on a subset of the GM population due to delays in data from some GM secondary care providers. The study population represents a highly deprived population placed under strict restrictions during the pandemic. While this information is valuable, the findings may not be generalizable to other settings in the UK or internationally.

Although the measures of HCU that have been selected are relevant and reliable, they do not provide a complete picture of either primary or secondary HCU. There is no single effective measure to summarise HCU in primary care as there are many aspects that reflect HCU in this setting.[35] It remains possible that the shift towards increased remote consultations may have resulted in changes to primary care delivery that were not possible to accurately capture using our measures of primary HCU. Additionally, the cause of admission was not available for secondary HCU, hence we were unable to determine LTC-specific admissions. Whilst the current scaling and sub-populations do not take into consideration any deaths or new diagnoses that occurred after 1 Jan 2020, a sensitivity analysis accounting for deaths resulted in very small increases to rates (Supplementary Figures S9-S10; Supplementary Table S12) and scaled utilisation remained lower than pre-lockdown levels.

Conclusions

We have assessed the changes in HCU in primary and secondary care associated with the COVID-19 pandemic and UK national lockdowns for patients with LTCs across a large urban region. There was a significant reduction in both primary and secondary HCU associated with the first national lockdown. Subsequent national lockdowns were associated with reductions in secondary care but not in primary care. Whilst some measures of healthcare utilisation had returned to pre-pandemic levels by the end of the study, many had not. Proportionally, secondary care HCU increased in multi-morbid patients compared to those without LTCs during the first and second national lockdowns. Although changes to HCU during the pandemic have been similar overall, different patterns have been seen in specific LTC groups such as people with cancer. Over the course of the pandemic deprivation was associated with higher rates of HCU in multi-morbid patients but no significant differences were observed in the ratio of utilisation between the most and least deprived groups for the majority of HCU measures during national lockdowns.

ACKNOWLEDGEMENTS

We would like to thank Eric Lowndes and Sumaira Naseem for providing the public representation throughout our research. The authors recognise the Greater Manchester Care Record (a partnership of Greater Manchester Health and Social Care Partnership, Health Innovation Manchester and Graphnet Health, on behalf of Greater Manchester localities) in the provision of data required to

For peer review only - http://bmjopen.bmj.com/site/about/guidelines.xhtml

undertake this work. Using patient data is vital to improve health and care for everyone. There is huge potential to make better use of information from people's patient records, to understand more about disease, develop new treatments, monitor safety, and plan NHS services. Patient data should be kept safe and secure, to protect everyone's privacy, and it's important that there are safeguards to make sure that it is stored and used responsibly. Everyone should be able to find out about how patient data is used.

CONTRIBUTIONS

SG conceived the study. NP and RW led on the acquisition of data for the study. All authors were involved in the design of the study and interpretation of the data. CSP led the analysis of the data, supported by MS. CSP and SG prepared the manuscript. All authors revised the manuscript and approved the final version.

FUNDING

The time of RW and NP was partially funded by the National Institute for Health Research (NIHR) Greater Manchester Patient Safety Translational Research Centre (PSTRC-2016-003) and the NIHR Applied Research Collaboration Greater Manchester (NIHR200174). The time of NP was partially funded by the NIHR Manchester Biomedical Research Centre (IS-BRC-1215-20007). The time of CSP was fully funded by the NIHR Applied Research Collaboration Greater Manchester (NIHR200174). The views expressed are those of the author(s) and not necessarily those of the NIHR or the Department of Health and Social Care.

COMPETING INTERESTS

All authors have completed the ICMJE uniform disclosure form at http://www.icmje.org/disclosure-of-interest/ and declare: no support from any organisation for the submitted work; no financial relationships with any organisations that might have an interest in the submitted work in the previous three years; no other relationships or activities that could appear to have influenced the submitted work.

ETHICS APPROVAL

All data made available to the analysts was de-identified and aggregated and therefore did not require specific ethical approval.

DATA SHARING STATEMENT

Data and code were hosted in a secure environment and are not freely available to share.

REFERENCES

1. Scally G, Jacobson B, Abbasi K. The UK's public health response to covid-19. *BMJ*. 2020;369(May):1-3. doi:10.1136/bmj.m1932

2. Dong E, Du H, Gardner L. An interactive web-based dashboard to track COVID-19 in real time. *Lancet Infect Dis*. 2020;20(5):533-534. doi:10.1016/S1473-3099(20)30120-1

3. Bou-Karroum L, Khabsa J, Jabbour M, et al. Public health effects of travel-related policies on the COVID-19 pandemic: A mixed-methods systematic review. *J Infect*. 2021;83(4):413-423. doi:10.1016/j.jinf.2021.07.017

4. Lee JK, Bullen C, Ben Amor Y, et al. Institutional and behaviour-change interventions to support COVID-19 public health measures: A review by the Lancet Commission Task Force on

For peer review only

public health measures to suppress the pandemic. *Int Health*. 2021;13(5):399-409. doi:10.1093/inthealth/ihab022

5. World Health Organization (WHO). *Maintaining Essential Health Services: Operational Guidance for the COVID-19 Context: Interim Guidance*.; 2020.

6. Blanchet K, Alwan A, Antoine C, et al. Protecting essential health services in low-income and middle-income countries and humanitarian settings while responding to the COVID-19 pandemic. *BMJ Glob Heal*. 2020;5(10):1-9. doi:10.1136/bmjgh-2020-003675

7. Doidge JC, Gould DW, Ferrando-Vivas P, et al. Trends in intensive care for patients with covid-19 in england, wales, and northern ireland. *Am J Respir Crit Care Med*. 2021;203(5):565-574. doi:10.1164/rccm.202008-3212OC

8. Mutambudzi M, Niedwiedz C, Macdonald EB, et al. Occupation and risk of severe COVID-19: Prospective cohort study of 120 075 UK Biobank participants. *Occup Environ Med*. 2021;78(5):307-314. doi:10.1136/oemed-2020-106731

9. Williams R, Jenkins DA, Ashcroft DM, et al. Diagnosis of physical and mental health conditions in primary care during the COVID-19 pandemic: a retrospective cohort study. *Lancet Public Heal*. 2020;5(10):e543-e550. doi:10.1016/S2468-2667(20)30201-2

10. Mahase E. Covid-19: Urgent cancer referrals fall by 60%, showing “brutal” impact of pandemic. *BMJ*. 2020;369(June):m2386. doi:10.1136/bmj.m2210

11. Mafham MM, Spata E, Goldacre R, et al. COVID-19 pandemic and admission rates for and management of acute coronary syndromes in England. *Lancet*. 2020;396(10248):381-389. doi:10.1016/S0140-6736(20)31356-8

12. Kwok CS, Gale CP, Kinnaird T, et al. Impact of COVID-19 on percutaneous coronary intervention for ST-elevation myocardial infarction. *Heart*. 2020;106(23):1805-1811. doi:10.1136/heartjnl-2020-317650

13. Nepogodiev D, Omar OM, Glasbey JC, et al. Elective surgery cancellations due to the COVID-19 pandemic: global predictive modelling to inform surgical recovery plans. *Br J Surg*. 2020;107(11):1440-1449. doi:10.1002/bjs.11746

14. Moynihan R, Sanders S, Michaleff ZA, et al. Impact of COVID-19 pandemic on utilisation of healthcare services: A systematic review. *BMJ Open*. 2021;11(3):11-17. doi:10.1136/bmjopen-2020-045343

15. Marmot M, Allen J, Boyce T, Goldblatt P, Morrison J. *Build Back Fairer in Greater Manchester : Health Equity and Dignified Lives*. London, UK: Institute of Health Equity; 2021.

16. UK Health Security Agency. Coronavirus (COVID-19) in the UK. https://coronavirus.data.gov.uk/details/download.

17. Department of Health and Social Care. *COPI Notice*.; 2020. https://digital.nhs.uk/coronavirus/coronavirus-covid-19-response-information-governance-hub/control-of-patient-information-copi-notice.

18. Barnett K, Mercer SW, Norbury M, Watt G, Wyke S, Guthrie B. Epidemiology of multimorbidity and implications for health care, research, and medical education: A cross-sectional study. *Lancet*. 2012;380(9836):37-43. doi:10.1016/S0140-6736(12)60240-2

19. Ministry of Housing Communities and Local Governments. *The English Indices of Deprivation Things You Need to Know (IoD2019)*.; 2019. https://assets.publishing.service.gov.uk/government/uploads/system/uploads/attachment_d

For peer review only - http://bmjopen.bmj.com/site/about/guidelines.xhtml

ata/file/835115/IoD2019_Statistical_Release.pdf.

20. Wickham H, Averick M, Bryan J, et al. Welcome to the Tidyverse. *J Open Source Softw*. 2019;4(43):1686. doi:10.21105/joss.01686

21. Wickham H, Seidel D. scales: Scale Functions for Visualization. 2020. https://cran.r-project.org/package=scales.

22. Wickham H. Reshaping data with the reshape package. *J Stat Softw*. 2007;21(12):1-20. doi:10.18637/jss.v021.i12

23. Wilke CO. cowplot: Streamlined Plot Theme and Plot Annotations for "ggplot2." 2020. https://cran.r-project.org/package=cowplot.

24. Lai AG, Pasea L, Banerjee A, et al. Estimated impact of the COVID-19 pandemic on cancer services and excess 1-year mortality in people with cancer and multimorbidity: Near real-time data on cancer care, cancer deaths and a population-based cohort study. *BMJ Open*. 2020;10(11):1-9. doi:10.1136/bmjopen-2020-043828

25. Ball S, Banerjee A, Berry C, et al. Monitoring indirect impact of COVID-19 pandemic on services for cardiovascular diseases in the UK. *Heart*. 2020;106(24):1890-1897. doi:10.1136/heartjnl-2020-317870

26. Hussain AZ, Paudyal V, Hadi MA. Impact of the COVID-19 pandemic on the prescribing patterns of first-line antibiotics in english primary care: A longitudinal analysis of national prescribing dataset. *Antibiotics*. 2021;10(5):4-12. doi:10.3390/antibiotics10050591

27. Sephton BM, Mahapatra P, Shenouda M, Ferran N, Deierl K, Sinnett T. The effect of COVID-19 on a Major Trauma Network. An analysis of mechanism of injury pattern, referral load and operative case-mix. *Injury*. 2021;52:395-401.

28. Öngür D, Perlis R, Goff D. Psychiatry and COVID-19. *J Am Med Assoc*. 2020;324(12):1149-1150. doi:10.1001/jama.2020.9740

29. Pinato DJ, Scotti L, Gennari A, et al. Determinants of enhanced vulnerability to coronavirus disease 2019 in UK patients with cancer: a European study. *Eur J Cancer*. 2021;150:190-202.

30. Sud A, Jones ME, Broggio J, et al. Collateral damage: the impact on outcomes from cancer surgery of the COVID-19 pandemic. *Ann Oncol*. 2020;31(8):1065-1074. doi:10.1016/B978-0-323-60984-5.00062-7

31. Campbell C, Sommerfield T, Clark GRC, et al. COVID-19 and cancer screening in Scotland: A national and coordinated approach to minimising harm. *Prev Med (Baltim)*. 2021;151(October):106606.

32. Kontopantelis E, Mamas MA, Webb RT, et al. Excess deaths from COVID-19 and other causes by region, neighbourhood deprivation level and place of death during the first 30 weeks of the pandemic in England and Wales: A retrospective registry study. *Lancet Reg Heal - Eur*. 2021;7:100144. doi:10.1016/j.lanepe.2021.100144

33. Park S, Elliott J, Berlin A, Hamer-Hunt J, Haines A. Strengthening the UK primary care response to covid-19. *BMJ*. 2020;370:3-6. doi:10.1136/bmj.m3691

34. Murphy M, Scott LJ, Salisbury C, et al. Implementation of remote consulting in UK primary care following the COVID-19 pandemic : a mixed-methods longitudinal study. *Br J Gen Pract*. 2021;(March):166-177.

35. Da Silva RB, Contandriopoulos AP, Pineault R, Tousignant P. A global approach to evaluation

For peer review only

of health services utilization: Concepts and measures. *Healthc Policy*. 2011;6(4):106-117. doi:10.12927/hcpol.2011.22351

FIGURE LEGENDS

Figure 1: Weekly primary care utilisation per 1000 people of the Greater Manchester population between January 2020 and August 2021. The first week covered 30th December 2019 to 5th January 2020, hence utilisation was expected to be considerably lower between this and the following week due to the UK bank holiday and seasonal effects expected for this calendar week.

Figure 2: Rates of primary care measures recorded per 1000 people per week, identified according to number of long-term conditions and deprivation group, between January 2020 and August 2021.

Figure 3: (A:) Weekly rates of planned and unplanned admissions per 1000 people that were identified as having zero, one or multiple LTCs, within the Manchester CCG subpopulation between January 2020 and August 2021. (B:) Government reported COVID-19 admissions in Manchester University NHS Foundation Trust, Pennine Acute Hospitals NHS Foundation Trust and Pennine Care NHS Foundation Trust (extracted 08-Sept-21), and (C:) Government reported cases in Manchester (extracted 08-Sept- 21). MFT = Manchester University Hospital Foundation Trust, Pennine = Pennine Acute Hospitals NHS Trust & Pennine Care NHS Foundation Trust

Figure 4: Weekly rates of planned and unplanned admissions identified in patients with each of the long-term conditions within the Manchester CCG subpopulation, between January 2020 and August 2021.

For peer review only

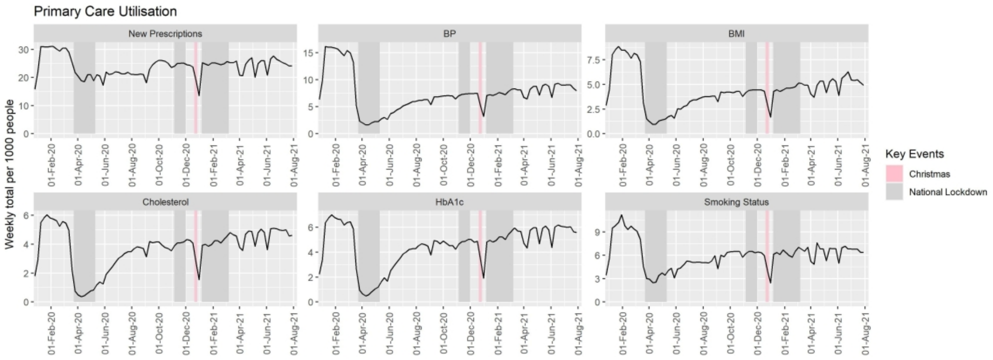

Figure 1:
Weekly primary care utilisation per 1000 people of the Greater Manchester population between January 2020 and August 2021. The first week covered 30th December 2019 to 5th January 2020, hence utilisation was expected to be considerably lower between this and the following week due to the UK bank holiday and seasonal effects expected for this calendar week.

338x127mm (300 x 300 DPI)

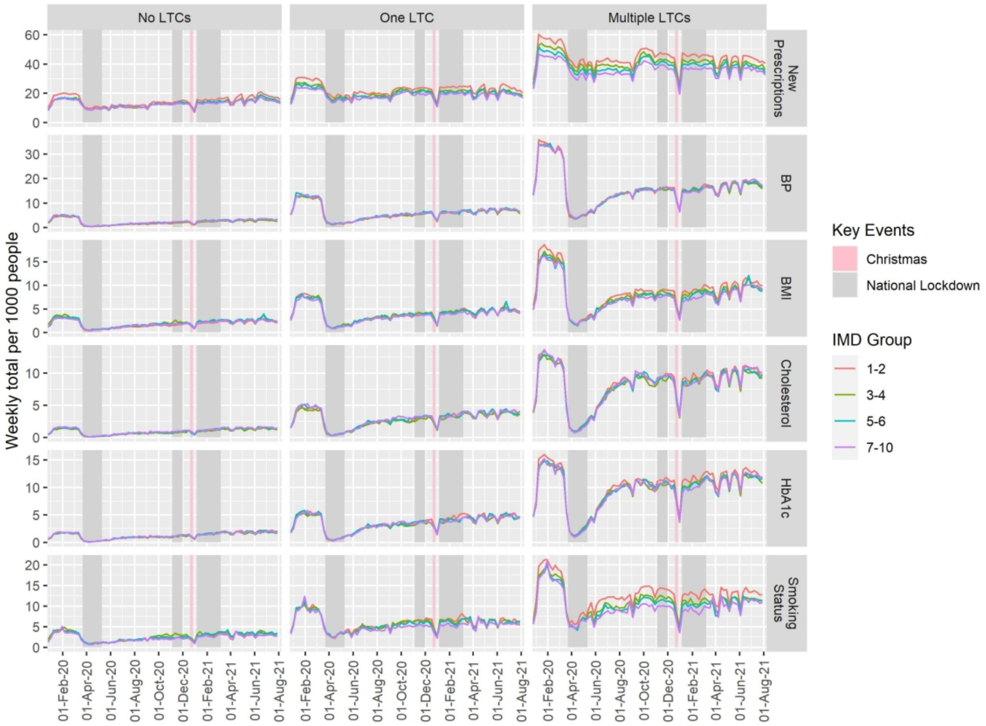

Figure 2: Rates of primary care measures recorded per 1000 people per week, identified according to number of long-term conditions and deprivation group, between January 2020 and August 2021.

232x181mm (350 x 350 DPI)

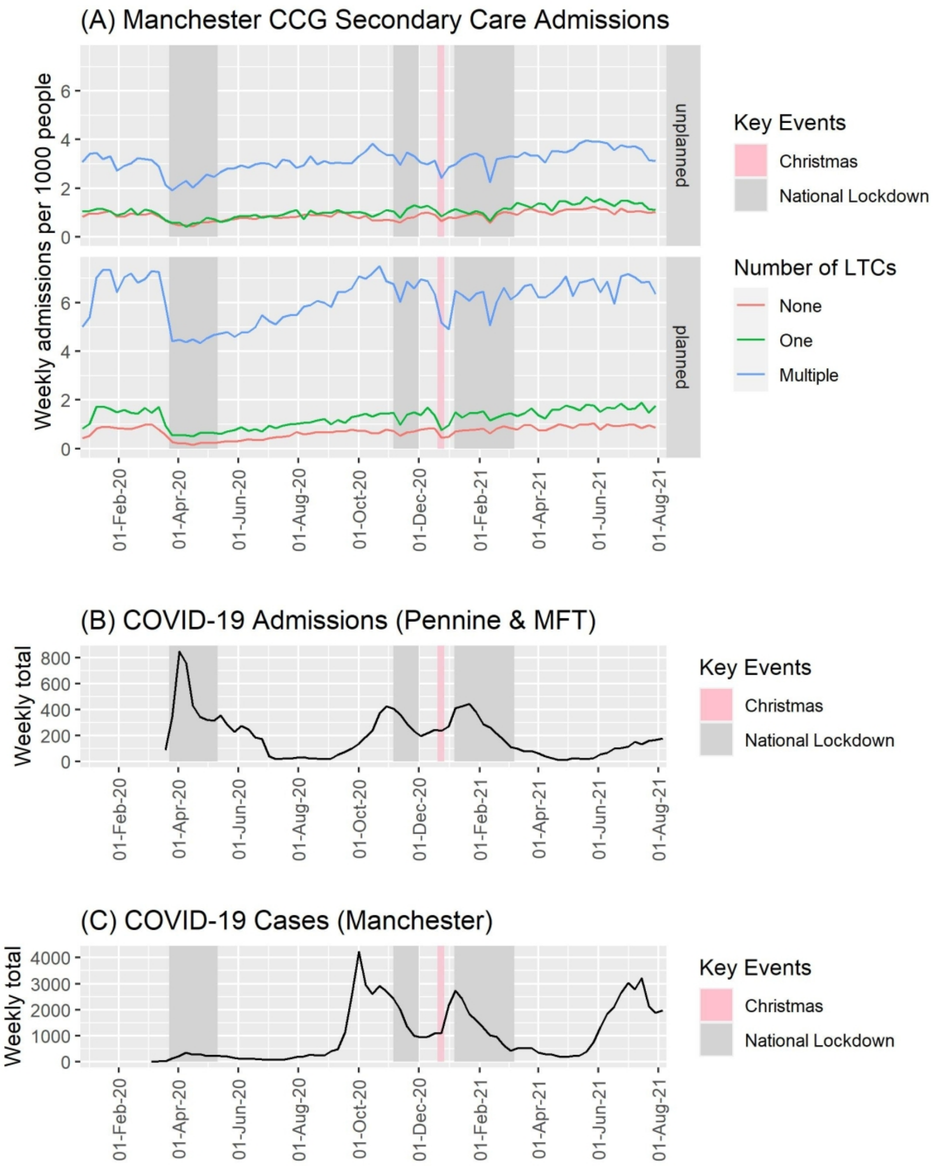

Figure 3: (A:) Weekly rates of planned and unplanned admissions per 1000 people that were identified as having zero, one or multiple LTCs, within the Manchester CCG subpopulation between January 2020 and August 2021. (B:) Government reported COVID-19 admissions in Manchester University NHS Foundation Trust, Pennine Acute Hospitals NHS Foundation Trust and Pennine Care NHS Foundation Trust (extracted 08-Sept-21), and (C:) Government reported cases in Manchester (extracted 08-Sept-21). MFT = Manchester University Hospital Foundation Trust, Pennine = Pennine Acute Hospitals NHS Trust & Pennine Care NHS Foundation Trust

169x211mm (300 x 300 DPI)

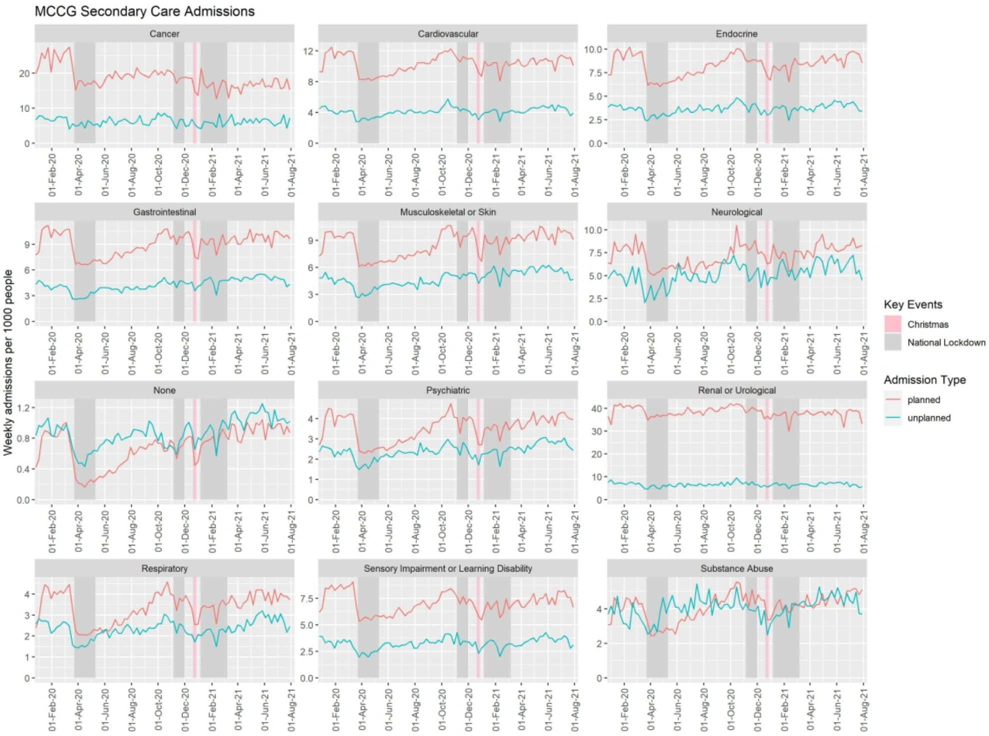

Figure 4: Weekly rates of planned and unplanned admissions identified in patients with each of the long-term conditions within the Manchester CCG subpopulation, between January 2020 and August 2021.

338x254mm (300 x 300 DPI)

SUPPLEMENTARY MATERIALS

COVID-19 Government data extraction

The Government data were extracted on 08-09-2021 from https://api.coronavirus.data.gov.uk/ . For the new cases, we extracted data for Manchester only. The API for Manchester cases by specimen date that was used is:

https://api.coronavirus.data.gov.uk/v2/data?areaType=utla&areaCode=E08000003&metric=newCasesBySpecimenDate&format=csv

For the secondary admissions, we exported the data for the trusts which were the main providers in the Manchester CCG subpopulation. The hospital groupings were not consistent across data sources; Table S2 provides the mapping between the Government listed NHS trusts and the secondary providers covering nearly all admissions experienced by Manchester CCG population in the GMCR.

Supplementary Table S1: Mapping between NHS acute providers according to data source

GMCR acute provider	Government listed NHS Trust	Government data API
University Hospital of South Manchester<br>Central Manchester University Hospitals	Manchester University NHS Foundation Trust	https://api.coronavirus.data.gov.uk/v2/data?areaType=nhsTrust&areaCode=R0A&metric=newAdmissions&format=csv
Pennine Acute Hospitals	Pennine Acute Hospitals NHS Trust	https://api.coronavirus.data.gov.uk/v2/data?areaType=nhsTrust&areaCode=RW6&metric=newAdmissions&format=csv
Pennine Acute Hospitals	Pennine Care NHS Foundation Trust	https://api.coronavirus.data.gov.uk/v2/data?areaType=nhsTrust&areaCode=RT2&metric=newAdmissions&format=csv

For peer review only

Supplementary Table S2: Long-term medical conditions and their groupings

Grouping	Long-term medical condition
**Cardiovascular**	Hypertension<br>Atrial fibrillation<br>Heart failure<br>Peripheral vascular disease<br>Stroke & transient ischaemic attack<br>Coronary heart disease
**Respiratory**	Bronchiectasis<br>Asthma<br>Chronic obstructive pulmonary disease<br>Chronic sinusitis
**Gastrointestinal**	Viral Hepatitis<br>Chronic liver disease<br>Inflammatory bowel disease<br>Diverticular disease of intestine<br>Treated constipation<br>Irritable bowel syndrome<br>Treated dyspepsia
**Neurological**	Multiple sclerosis<br>Parkinson's disease<br>Migraine<br>Epilepsy
**Endocrine**	Thyroid disorders<br>Diabetes
**Psychiatric**	Anorexia or bulimia<br>Schizophrenia (and related non-organic psychosis) or bipolar disorder<br>Dementia<br>Anxiety & other neurotic, stress related & somatoform disorders<br>Depression
**Substance Abuse**	Alcohol problems<br>Other psychoactive substance misuse
**Musculoskeletal/Skin**	Psoriasis or eczema<br>Rheumatoid arthritis, other inflammatory polyarthropathies & systematic connective tissue disorders<br>Painful condition
**Sensory impairment or learning disability**	Learning disability<br>Blindness & low vision<br>Glaucoma<br>Hearing loss
**Renal/Urological**	Chronic kidney disease<br>Prostate disorders

For peer review only

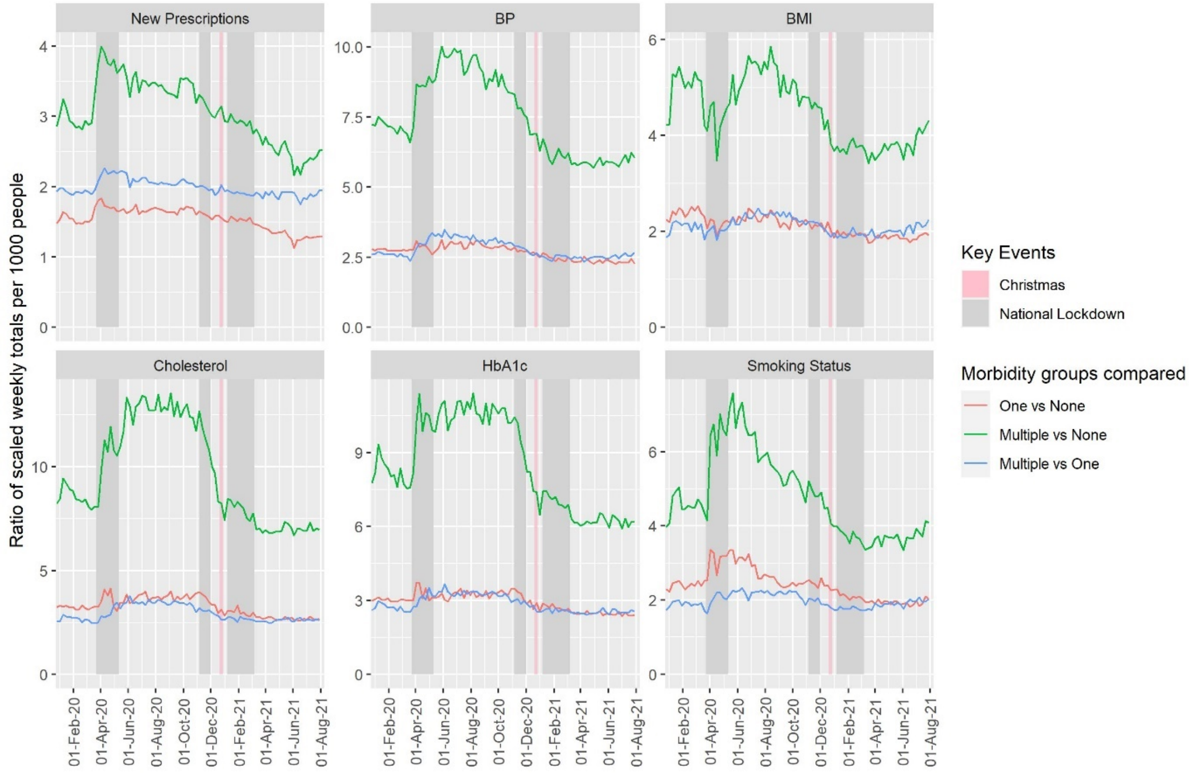

Supplementary Figure S1: Ratio of weekly primary care HCU measures per 1000 people between morbidity groups, between January 2020 and August 2021.

review only

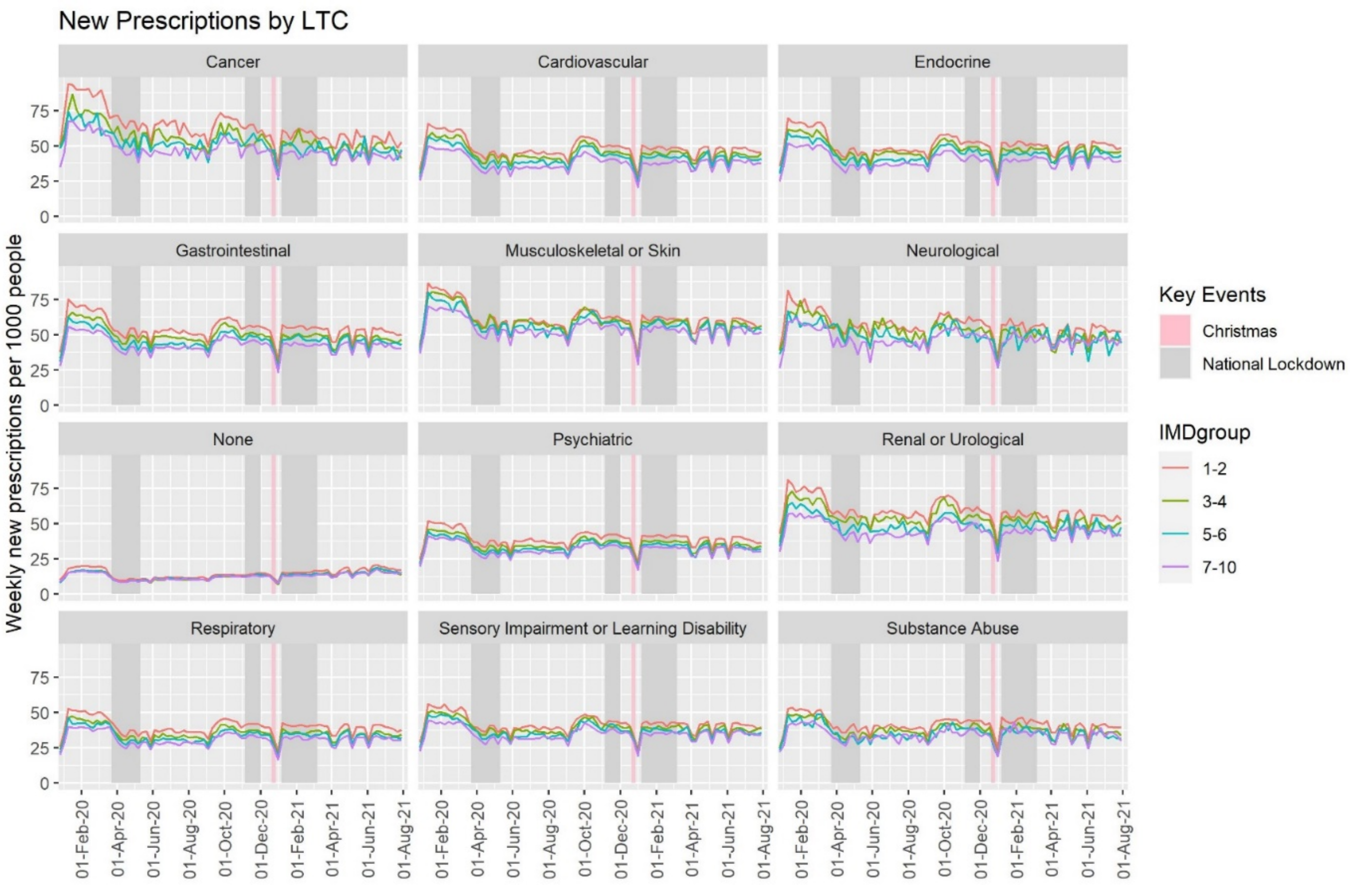

Supplementary Figure S2: Weekly new primary care prescriptions per 1000 people within each long-term condition group and deprivation group, between January 2020 and August 2021.

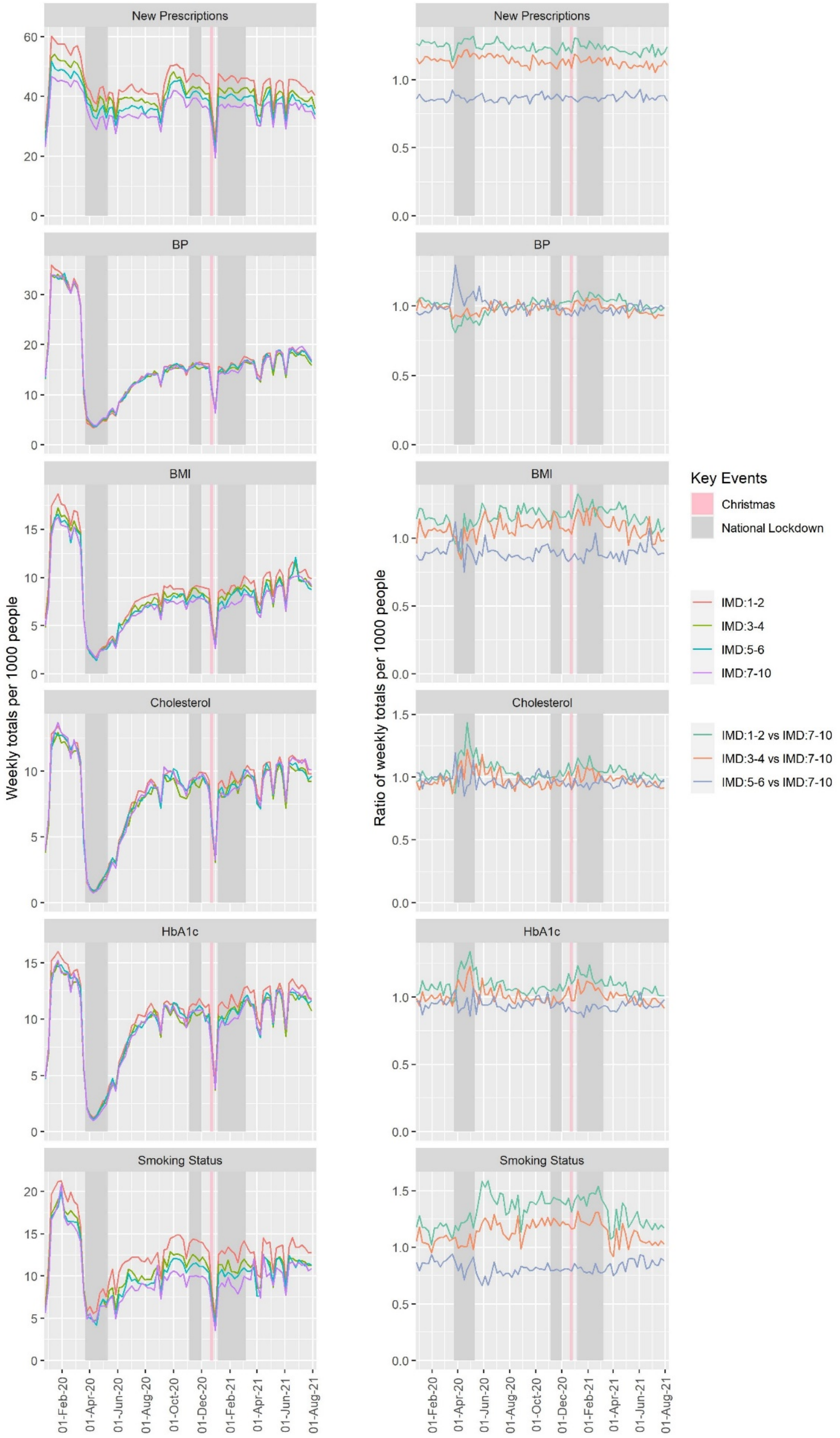

Supplementary Figure S3: Weekly new primary care HCU measures per 1000 people within multi-morbid patients by deprivation group and the ratio of these compared to the least-deprived group (IMD: 7-10), between January 2020 and August 2021.

Supplementary Table S3: Associated effects of the national lockdowns on primary HCU compare to pre-pandemic HCU. NL1 = First national lockdown, NL2 = Second national lockdown, NL3 = Third national lockdown.

	Rate Ratio	95% CI	p-value
	**New Prescriptions**		
NL1 vs pre-pandemic	0.681	0.679 – 0.684	**<0.001**
NL2 vs pre-pandemic	0.835	0.832 – 0.838	**<0.001**
NL3 vs pre-pandemic	0.843	0.841 – 0.846	**<0.001**
	**BP**		
NL1 vs pre-pandemic	0.143	0.142 – 0.144	**<0.001**
NL2 vs pre-pandemic	0.494	0.491 – 0.497	**<0.001**
NL3 vs pre-pandemic	0.510	0.508 – 0.513	**<0.001**
	**BMI**		
NL1 vs pre-pandemic	0.168	0.167 – 0.170	**<0.001**
NL2 vs pre-pandemic	0.559	0.555 – 0.564	**<0.001**
NL3 vs pre-pandemic	0.592	0.588 – 0.596	**<0.001**
	**Cholesterol**		
NL1 vs pre-pandemic	0.128	0.126 – 0.130	**<0.001**
NL2 vs pre-pandemic	0.769	0.762 – 0.776	**<0.001**
NL3 vs pre-pandemic	0.788	0.782 – 0.793	**<0.001**
	**HbA1c**		
NL1 vs pre-pandemic	0.148	0.146 – 0.150	**<0.001**
NL2 vs pre-pandemic	0.785	0.778 – 0.791	**<0.001**
NL3 vs pre-pandemic	0.837	0.832 – 0.842	**<0.001**
	**Smoking Status**		
NL1 vs pre-pandemic	0.346	0.344 – 0.349	**<0.001**
NL2 vs pre-pandemic	0.692	0.687 – 0.696	**<0.001**
NL3 vs pre-pandemic	0.689	0.685 – 0.692	**<0.001**
	**Planned Admissions**		
NL1 vs pre-pandemic	0.544	0.530 – 0.559	**<0.001**
NL2 vs pre-pandemic	0.921	0.897 – 0.946	**<0.001**
NL3 vs pre-pandemic	0.902	0.883 – 0.922	**<0.001**
	**Unplanned Admissions**		
NL1 vs pre-pandemic	0.659	0.638 – 0.681	**<0.001**
NL2 vs pre-pandemic	0.953	0.922 – 0.986	**0.005**
NL3 vs pre-pandemic	0.984	0.958 – 1.010	0.227

For peer review only

Supplementary Table S4: Percentage decrease (95%CI) in HCU in 2021 compared with the same calendar week in 2020.

Calendar Week	2	3	4	5	6	7	8	9	10	11	Average	p-value
**Primary HCU**												
New prescriptions	20.0 (19.3 – 20.8)	21.2 (20.5 – 21.9)	18.4 (17.7 – 19.2)	18.7 (18.0 – 19.5)	19.6 (18.9 – 20.3)	18.7 (17.9 – 19.5)	15.5 (14.8 – 16.3)	15.5 (14.8 – 16.3)	17.2 (16.5 – 18.0)	12.8 (12.0 – 13.6)	17.8 (17.6 – 18.1)	<0.001
Blood Pressure	55.2 (54.5 – 55.9)	55.8 (55.1 – 56.5)	54.4 (53.7 – 55.1)	52.0 (51.3 – 52.7)	52.4 (51.6 – 53.1)	52.1 (51.4 – 52.9)	45.9 (45.0 – 46.7)	46.4 (45.7 – 47.2)	44.3 (43.4 – 45.1)	38.7 (37.8 – 39.7)	50.0 (49.8 – 50.2)	<0.001
BMI	44.4 (43.3 – 45.5)	49.7 (48.7 – 50.8)	49.7 (48.7 – 50.8)	45.3 (44.3 – 46.5)	45.7 (44.6 – 46.8)	43.2 (42.0 – 44.3)	37.8 (36.6 – 39.1)	36.7 (35.6 – 38.0)	36.3 (35.1 – 37.6)	32.6 (31.3 – 34.0)	42.5 (42.1 – 42.8)	<0.001
Cholesterol	27.5 (25.8 – 29.1)	34.0 (32.4 – 35.4)	34.2 (32.7 – 35.6)	26.9 (25.3 – 28.5)	26.2 (24.5 – 27.7)	27.4 (25.8 – 29.1)	17.1 (15.3 – 19.0)	18.0 (16.2 – 19.8)	11.9 (10.0 – 13.8)	6.3 (4.2 – 8.4)	18.4 (17.8 – 18.9)	<0.001
HbA1c	22.0 (20.4 – 23.6)	28.6 (27.2 – 30.1)	29.4 (27.9 – 30.8)	23.0 (21.5 – 24.6)	21.7 (20.1 – 23.2)	24.1 (22.6 – 25.7)	12.4 (10.6 – 14.2)	10.7 (9.0 – 12.5)	7.7 (5.9 – 9.5)	2.7 (0.8 – 4.7)	18.6 (18.1 – 19.1)	<0.001
Smoking Status	33.3 (32.1 – 34.5)	37.8 (36.7 – 38.9)	34.7 (33.6 – 35.9)	43.4 (42.6 – 44.5)	38.9 (37.8 – 40.0)	38.4 (37.3 – 39.6)	32.9 (31.8 – 34.2)	24.7 (23.5 – 26.1)	25.1 (23.9 – 26.6)	18.8 (17.4 – 20.4)	33.3 (33.0 – 33.7)	<0.001
**Secondary HCU**												
Planned admissions	11.6 (5.3 – 17.5)	16.8 (10.9 – 22.3)	12.5 (6.3 – 18.2)	-0.4 (-7.6 – 6.3)	27.1 (21.6 – 32.3)	12.5 (6.2 – 18.4)	1.7 (-5.2 – 8.2)	12.5 (6.3 – 18.3)	14.8 (8.9 – 20.4)	2.6 (-4.1 – 8.9)	11.3 (9.4 – 13.2)	<0.001
Unplanned admissions	8.4 (0.2 – 15.9)	3.3 (-5.2 – 11.2)	11.2 (-7.4 – 9.1)	-14.2 (-24.9 – -4.4)	26.9 (19.4 – 33.8)	2.0 (-6.8 – 10.1)	-7.7 (-17.2 – 1.1)	-5.7 (-15.1 – 2.9)	-3.1 (-12.2 – 5.2)	-25.7 (-37.0 – -15.5)	-1.2 (-4.0 – 1.5)	0.376

For peer review only

Supplementary Table S5: Associated effects of morbidity on the rates of weekly totals of primary and secondary HCU (per 1000 people), throughout the study period. LTC = Long term condition.

	Rate Ratio	95% CI	p-value
	**New Prescriptions**		
Multiple LTCs vs No LTCs	3.040	2.881 – 3.208	<0.001
One LTC vs No LTCs	1.534	1.454 – 1.619	<0.001
	**BP**		
Multiple LTCs vs No LTCs	7.347	6.197 – 8.711	<0.001
One LTC vs No LTCs	2.659	2.243 – 3.153	<0.001
	**BMI**		
Multiple LTCs vs No LTCs	4.429	3.801 – 5.162	<0.001
One LTC vs No LTCs	2.106	1.807 – 2.454	<0.001
	**Cholesterol**		
Multiple LTCs vs No LTCs	9.360	7.654 – 11.446	<0.001
One LTC vs No LTCs	3.226	2.638 – 3.945	<0.001
	**HbA1c**		
Multiple LTCs vs No LTCs	8.291	6.847 – 10.038	<0.001
One LTC vs No LTCs	2.918	2.410 – 3.533	<0.001
	**Smoking Status**		
Multiple LTCs vs No LTCs	4.674	4.192 – 5.211	<0.001
One LTC vs No LTCs	2.372	2.127 – 2.645	<0.001
	**Planned Admissions**		
Multiple LTCs vs No LTCs	9.584	8.644 – 10.627	<0.001
One LTC vs No LTCs	1.904	1.717 – 2.111	<0.001
	**Unplanned Admissions**		
Multiple LTCs vs No LTCs	3.636	3.401 – 3.887	<0.001
One LTC vs No LTCs	1.188	1.112 – 1.270	<0.001

For peer review only

Supplementary Table S6: Estimated rate ratios (RRs) from log-linear regression models for each HCU measure observed pre-pandemic and in the national lockdowns, adjusted for the number of long term conditions (LTCs). NL1 = First national lockdown, NL2 = Second national lockdown, NL3 = Third national lockdown.

	**Primary Care**											
	**New Prescriptions**			**BP**			**BMI**			**Cholesterol**		
	RR	95% CI	p-value	RR	95% CI	p-value	RR	95% CI	p-value	RR	95% CI	p-value
NL1 vs pre-pandemic	0.580	0.543 - 0.619	**<0.001**	0.126	0.112 - 0.141	**<0.001**	0.190	0.165 - 0.218	**<0.001**	0.103	0.084 - 0.127	**<0.001**
NL2 vs pre-pandemic	0.800	0.742 - 0.862	**<0.001**	0.469	0.409 - 0.537	**<0.001**	0.615	0.523 - 0.722	**<0.001**	0.624	0.490 - 0.796	**<0.001**
NL3 vs pre-pandemic	0.855	0.804 - 0.909	**<0.001**	0.581	0.520 - 0.648	**<0.001**	0.752	0.660 - 0.856	**<0.001**	0.859	0.705 - 1.046	**0.128**
Multiple LTCs vs No LTCs	2.950	2.779 - 3.131	**<0.001**	7.155	6.429 - 7.963	**<0.001**	5.091	4.483 - 5.781	**<0.001**	8.576	7.077 - 10.392	**<0.001**
One LTC vs No LTCs	1.531	1.442 - 1.625	**<0.001**	2.753	2.474 - 3.064	**<0.001**	2.400	2.114 - 2.726	**<0.001**	3.222	2.659 - 3.904	**<0.001**
NL1 vs pre-pandemic : Multiple LTCs vs No LTCs	1.281	1.169 - 1.404	**<0.001**	1.187	1.007 - 1.400	**0.042**	0.847	0.696 - 1.030	0.095	1.220	0.907 - 1.640	0.186
NL2 vs pre-pandemic : Multiple LTCs vs No LTCs	1.073	0.964 - 1.193	0.194	1.091	0.901 - 1.321	0.369	0.911	0.726 - 1.144	0.418	1.318	0.934 - 1.858	0.114
NL3 vs pre-pandemic : Multiple LTCs vs No LTCs	0.984	0.903 - 1.073	0.713	0.859	0.736 - 1.004	0.056	0.736	0.613 - 0.885	**0.001**	0.920	0.697 - 1.216	0.555
NL1 vs pre-pandemic : One LTC vs No LTCs	1.126	1.027 - 1.234	**0.012**	1.025	0.870 - 1.209	0.763	0.889	0.731 - 1.081	0.236	1.102	0.820 - 1.482	0.515
NL2 vs pre-pandemic : One LTC vs No LTCs	1.043	0.937 - 1.160	0.439	0.996	0.823 - 1.206	0.968	0.888	0.708 - 1.115	0.304	1.139	0.808 - 1.606	0.454
NL3 vs pre-pandemic : One LTC vs No LTCs	0.994	0.912 - 1.084	0.895	0.896	0.767 - 1.046	0.162	0.811	0.674 - 0.975	**0.026**	0.921	0.697 - 1.216	0.557
	**Primary Care (ctd)**						**Secondary Care**					
	**HbA1c**			**Smoking Status**			**Planned Admissions**			**Unplanned Admissions**		
	RR	95% CI	p-value	RR	95% CI	p-value	RR	95% CI	p-value	RR	95% CI	p-value

NL1 vs pre-pandemic	0.120	0.096 - 0.148	**<0.001**	0.272	0.238 - 0.310	**<0.001**	0.272	0.243 - 0.305	**<0.001**	0.582	0.511 - 0.663	**<0.001**
NL2 vs pre-pandemic	0.716	0.557 - 0.920	**0.010**	0.667	0.573 - 0.777	**<0.001**	0.820	0.719 - 0.935	**0.003**	0.803	0.690 - 0.934	**0.005**
NL3 vs pre-pandemic	0.967	0.789 - 1.185	0.745	0.816	0.721 - 0.923	**<0.001**	0.935	0.841 - 1.040	0.214	0.933	0.826 - 1.055	0.267
Multiple LTCs vs No LTCs	8.223	6.744 - 10.026	**<0.001**	4.595	4.074 - 5.183	**<0.001**	8.298	7.481 - 9.205	**<0.001**	3.359	2.981 - 3.786	**<0.001**
One LTC vs No LTCs	3.022	2.478 - 3.684	**<0.001**	2.404	2.131 - 2.712	**<0.001**	1.834	1.653 - 2.034	**<0.001**	1.111	0.986 - 1.252	0.083
NL1 vs pre-pandemic : Multiple LTCs vs No LTCs	1.221	0.900 - 1.658	0.197	1.356	1.126 - 1.632	**0.002**	2.402	2.047 - 2.818	**<0.001**	1.253	1.042 - 1.506	**0.017**
NL2 vs pre-pandemic : Multiple LTCs vs No LTCs	1.145	0.803 - 1.633	0.449	1.074	0.866 - 1.332	0.511	1.174	0.975 - 1.413	0.090	1.283	1.036 - 1.588	**0.023**
NL3 vs pre-pandemic : Multiple LTCs vs No LTCs	0.852	0.639 - 1.136	0.271	0.803	0.675 - 0.956	**0.014**	0.957	0.824 - 1.112	0.563	1.073	0.902 - 1.276	0.421
NL1 vs pre-pandemic : One LTC vs No LTCs	1.082	0.797 - 1.468	0.610	1.279	1.062 - 1.540	**0.010**	1.413	1.205 - 1.658	**<0.001**	0.987	0.821 - 1.186	0.885
NL2 vs pre-pandemic : One LTC vs No LTCs	1.058	0.742 - 1.508	0.754	1.028	0.829 - 1.275	0.799	1.063	0.883 - 1.280	0.513	1.295	1.046 - 1.604	**0.018**
NL3 vs pre-pandemic : One LTC vs No LTCs	0.894	0.671 - 1.192	0.442	0.87	0.730 - 1.036	0.116	0.971	0.835 - 1.128	0.695	1.075	0.904 - 1.279	0.407

For peer review only

Supplementary Table S7: Associated effects of deprivation groups on primary and secondary HCU, throughout the study.

	**Primary Care**											
Deprivation group compared to group 1-2	**New Prescriptions**			**BP**			**BMI**			**Cholesterol**		
	RR	95% CI	p-value	RR	95% CI	p-value	RR	95% CI	p-value	RR	95% CI	p-value
3-4	0.915	0.874 – 0.959	**<0.001**	1.018	0.869 – 1.193	0.821	1.007	0.865 – 1.172	0.928	0.964	0.797 – 1.166	0.702
5-6	0.920	0.878 – 0.964	**<0.001**	1.117	0.954 – 1.309	0.169	1.025	0.881 – 1.192	0.751	1.076	0.889 – 1.302	0.450
7-10	0.875	0.835 – 0.917	**<0.001**	1.134	0.968 – 1.328	0.120	0.988	0.849 – 1.150	0.879	1.091	0.902 – 1.320	0.369
	**Primary Care (ctd)**						**Secondary Care**					
	**HbA1c**			**Smoking Status**			**Planned Admissions**			**Unplanned Admissions**		
	RR	95% CI	p-value	RR	95% CI	p-value	RR	95% CI	p-value	RR	95% CI	p-value
3-4	0.950	0.794 – 1.137	0.574	0.947	0.858 – 1.047	0.287	0.753	0.694 – 0.818	<0.001	0.686	0.642 – 0.732	<0.001
5-6	1.038	0.868 – 1.242	0.682	0.945	0.856 – 1.045	0.269	0.787	0.724 – 0.854	<0.001	0.67	0.628 – 0.715	<0.001
7-10	1.031	0.862 – 1.234	0.737	0.885	0.801 – 0.977	0.016	0.812	0.748 – 0.882	<0.001	0.683	0.640 – 0.729	<0.001

For peer review only

Supplementary Table S8: Comparison of HCU between deprivation groups and the highly deprived population throughout the pandemic for multi-morbid patients.

	Deprivation 3-4 vs 1-2		Deprivation 5-6 vs 1-2		Deprivation 7-10 vs 1-2	
	RR (95% CI)	p-value	RR (95% CI)	p-value	RR (95% CI)	p-value
**Primary HCU**						
New prescriptions	0.917 (0.878 – 0.957)	**<0.001**	0.867 (0.830 – 0.905)	**<0.001**	0.807 (0.773 – 0.843)	**<0.001**
BP	0.983 (0.844 – 1.145)	0.827	0.997 (0.856 – 1.161)	0.969	1.001 (0.860 – 1.166)	0.989
BMI	0.927 (0.796 – 1.081)	0.334	0.893 (0.766 – 1.041)	0.147	0.865 (0.742 – 1.008)	0.063
Cholesterol	0.939 (0.779 – 1.133)	0.510	0.969 (0.803 – 1.168)	0.738	0.958 (0.794 – 1.155)	0.652
HbA1c	0.927 (0.779 – 1.104)	0.396	0.942 (0.791 – 1.122)	0.504	0.919 (0.772 – 1.094)	0.342
Smoking status	0.863 (0.785 – 0.949)	**0.003**	0.820 (0.745 – 0.901)	**<0.001**	0.766 (0.697 – 0.843)	**<0.001**
**Secondary HCU**						
Planned admissions	0.873 (0.773 – 0.986)	**0.029**	0.741 (0.652 – 0.842)	**<0.001**	0.705 (0.619 – 0.802)	**<0.001**
Unplanned admissions	0.826 (0.696 – 0.980)	**0.029**	0.669 (0.557 – 0.802)	**<0.001**	0.645 (0.536 – 0.774)	**<0.001**

For peer review only

Supplementary Table S9: Estimated rate ratios (RRs) from log-linear regression models for each HCU observed pre-pandemic and in the national lockdowns, adjusted for the deprivation group. NL1 = First national lockdown, NL2 = Second national lockdown, NL3 = Third national lockdown, IMD = index of multiple deprivation.

	**Primary Care**											
	**New Prescriptions**			**BP**			**BMI**			**Cholesterol**		
	RR	95% CI	p-value	RR	95% CI	p-value	RR	95% CI	p-value	RR	95% CI	p-value
NL1 vs pre-pandemic	0.668	0.629 – 0.711	**<0.001**	0.133	0.118 – 0.149	**<0.001**	0.161	0.139 – 0.186	**<0.001**	0.125	0.102 – 0.154	**<0.001**
NL2 vs pre-pandemic	0.819	0.763 – 0.879	**<0.001**	0.491	0.430 – 0.560	**<0.001**	0.555	0.469 – 0.656	**<0.001**	0.782	0.615 – 0.994	**0.045**
NL3 vs pre-pandemic	0.837	0.790 – 0.886	**<0.001**	0.520	0.467 – 0.579	**<0.001**	0.599	0.523 – 0.686	**<0.001**	0.824	0.678 – 1.001	0.051
IMD: 3-4 vs IMD: 1-2	0.895	0.846 – 0.947	**<0.001**	1.011	0.910 – 1.123	0.837	0.994	0.871 – 1.135	0.933	0.977	0.808 – 1.181	0.810
IMD: 5-6 vs IMD: 1-2	0.891	0.843 – 0.943	**<0.001**	1.086	0.978 – 1.206	0.122	0.998	0.874 – 1.139	0.971	1.087	0.899 – 1.314	0.389
IMD: 7-10 vs IMD: 1-2	0.846	0.800 – 0.895	**<0.001**	1.099	0.989 – 1.220	0.079	0.977	0.856 – 1.116	0.732	1.128	0.933 – 1.364	0.213
NL1 vs pre-pandemic : IMD: 3-4 vs IMD: 1-2	1.043	0.956 – 1.137	0.342	1.076	0.916 – 1.265	0.372	1.056	0.861 – 1.296	0.600	0.976	0.729 – 1.308	0.871
NL2 vs pre-pandemic : IMD: 3-4 vs IMD: 1-2	1.033	0.935 – 1.143	0.522	1.006	0.834 – 1.214	0.947	1.070	0.845 – 1.357	0.574	0.984	0.701 – 1.382	0.926
NL3 vs pre-pandemic : IMD: 3-4 vs IMD: 1-2	1.021	0.941 – 1.108	0.617	0.981	0.843 – 1.142	0.806	1.021	0.843 – 1.237	0.831	0.978	0.743 – 1.287	0.872
NL1 vs pre-pandemic : IMD: 5-6 vs IMD: 1-2	1.043	0.956 – 1.137	0.342	1.124	0.956 – 1.320	0.158	1.064	0.867 – 1.305	0.553	1.037	0.774 – 1.389	0.810
NL2 vs pre-pandemic : IMD: 5-6 vs IMD: 1-2	1.048	0.948 – 1.159	0.360	1.029	0.853 – 1.241	0.765	1.022	0.806 – 1.295	0.857	1.000	0.712 – 1.404	0.999
NL3 vs pre-pandemic : IMD: 5-6 vs IMD: 1-2	1.025	0.944 – 1.112	0.559	1.002	0.861 – 1.166	0.980	1.025	0.846 – 1.242	0.803	0.960	0.729 – 1.264	0.773
NL1 vs pre-pandemic : IMD: 7-10 vs IMD: 1-2	1.034	0.949 – 1.128	0.444	1.159	0.986 – 1.363	0.073	1.088	0.887 – 1.334	0.419	0.889	0.663 – 1.191	0.430
NL2 vs pre-pandemic : IMD: 7-10 vs IMD: 1-2	1.048	0.948 – 1.159	0.362	1.037	0.860 – 1.251	0.701	1.018	0.803 – 1.290	0.885	1.006	0.716 – 1.413	0.973
NL3 vs pre-pandemic : IMD: 7-10 vs IMD: 1-2	1.019	0.939 – 1.106	0.648	0.969	0.833 – 1.128	0.686	0.971	0.801 – 1.177	0.764	0.923	0.701 – 1.216	0.570
	**Primary Care (ctd)**						**Secondary Care**					
	**HbA1c**			**Smoking Status**			**Planned Admissions**			**Unplanned Admissions**		

For peer review only

	RR	95% CI	p-value	RR	95% CI	p-value	RR	95% CI	p-value	RR	95% CI	p-value
NL1 vs pre-pandemic	0.142	0.115 – 0.175	**<0.001**	0.352	0.309 – 0.401	**<0.001**	**0.566**	**0.516 - 0.621**	**<0.001**	**0.642**	**0.558 - 0.737**	**<0.001**
NL2 vs pre-pandemic	0.794	0.622 – 1.013	0.064	0.734	0.632 – 0.853	**<0.001**	0.916	0.823 - 1.020	0.110	0.944	0.803 - 1.109	0.479
NL3 vs pre-pandemic	0.871	0.714 – 1.061	0.171	0.735	0.651 – 0.831	**<0.001**	**0.894**	**0.820 - 0.976**	**0.012**	0.945	0.829 - 1.077	0.393
IMD: 3-4 vs IMD: 1-2	0.947	0.781 – 1.149	0.582	0.977	0.868 – 1.101	0.707	**0.761**	**0.699 - 0.828**	**<0.001**	0.654	0.576 - 0.743	**<0.001**
IMD: 5-6 vs IMD: 1-2	1.038	0.856 – 1.259	0.705	0.978	0.868 – 1.101	0.713	**0.799**	**0.734 - 0.870**	**<0.001**	**0.598**	**0.527 - 0.680**	**<0.001**
IMD: 7-10 vs IMD: 1-2	1.041	0.858 – 1.263	0.681	0.972	0.863 – 1.094	0.636	**0.848**	**0.779 - 0.923**	**<0.001**	**0.643**	**0.566 - 0.731**	**<0.001**
NL1 vs pre-pandemic : IMD: 3-4 vs IMD: 1-2	1.000	0.743 – 1.347	0.999	0.958	0.798 – 1.150	0.646	0.988	0.867 - 1.126	0.858	1.097	0.902 - 1.335	0.351
NL2 vs pre-pandemic : IMD: 3-4 vs IMD: 1-2	0.991	0.702 – 1.401	0.961	0.997	0.806 – 1.233	0.977	1.001	0.860 - 1.165	0.990	1.017	0.810 - 1.277	0.883
NL3 vs pre-pandemic : IMD: 3-4 vs IMD: 1-2	0.991	0.749 – 1.311	0.949	0.97	0.816 – 1.152	0.725	0.997	0.881 - 1.127	0.959	1.061	0.882 - 1.276	0.525
NL1 vs pre-pandemic : IMD: 5-6 vs IMD: 1-2	1.020	0.758 – 1.374	0.895	0.974	0.811 – 1.170	0.779	0.765	0.671 - 0.872	**<0.001**	1.098	0.902 - 1.336	0.348
NL2 vs pre-pandemic : IMD: 5-6 vs IMD: 1-2	1.009	0.714 – 1.426	0.958	0.933	0.754 – 1.153	0.519	1.043	0.896 - 1.214	0.588	0.981	0.781 - 1.233	0.871
NL3 vs pre-pandemic : IMD: 5-6 vs IMD: 1-2	0.960	0.726 – 1.270	0.776	0.948	0.798 – 1.126	0.541	1.077	0.952 - 1.218	0.235	1.247	1.037 - 1.500	**0.020**
NL1 vs pre-pandemic : IMD: 7-10 vs IMD: 1-2	0.929	0.690 – 1.251	0.629	0.984	0.819 – 1.181	0.861	0.695	0.609 - 0.792	**<0.001**	0.898	0.738 - 1.093	0.280
NL2 vs pre-pandemic : IMD: 7-10 vs IMD: 1-2	1.019	0.721 – 1.440	0.915	0.852	0.689 – 1.054	0.139	1.032	0.887 - 1.202	0.678	1.173	0.934 - 1.473	0.169
NL3 vs pre-pandemic : IMD: 7-10 vs IMD: 1-2	0.927	0.700 – 1.226	0.595	0.836	0.704 – 0.994	**0.042**	1.068	0.944 - 1.208	0.291	1.164	0.968 - 1.401	0.105

For peer review only

Supplementary Table S10: Associated interaction between the first national lockdown and deprivation group on primary HCU compared to pre-pandemic rate ratios between deprivation groups, for multi-morbid patients.

	Deprivation 3-4 vs 1-2		Deprivation 5-6 vs 1-2		Deprivation 7-10 vs 1-2	
	RR (95% CI)	p-value	RR (95% CI)	p-value	RR (95% CI)	p-value
**Primary HCU**						
New prescriptions	1.022 (0.913 – 1.145)	0.697	1.012 (0.904 – 1.133)	0.831	0.992 (0.886 – 1.111)	0.888
BP	1.097 (0.886 – 1.358)	0.390	1.127 (0.910 – 1.396)	0.267	1.166 (0.942 – 1.444)	0.156
BMI	1.018 (0.760 – 1.362)	0.905	1.025 (0.766 – 1.372)	0.865	1.102 (0.823 – 1.475)	0.508
Cholesterol	0.958 (0.644 – 1.424)	0.829	1.004 (0.675 – 1.492)	0.986	0.853 (0.574 – 1.269)	0.428
HbA1c	0.988 (0.665 – 1.467)	0.950	0.996 (0.670 – 1.479)	0.983	0.890 (0.599 – 1.323)	0.561
Smoking status	0.939 (0.726 – 1.215)	0.628	0.945 (0.731 – 1.223)	0.665	0.957 (0.739 – 1.238)	0.733
**Secondary HCU**						
Planned admissions	1.011 (0.576 – 1.770)	0.970	0.748 (0.392 – 1.404)	0.371	0.731 (0.387 – 1.359)	0.326
Unplanned admissions	1.153 (0.513 – 2.592)	0.729	1.202 (0.490 – 2.919)	0.685	0.889 (0.346 – 2.217)	0.803

For peer review only

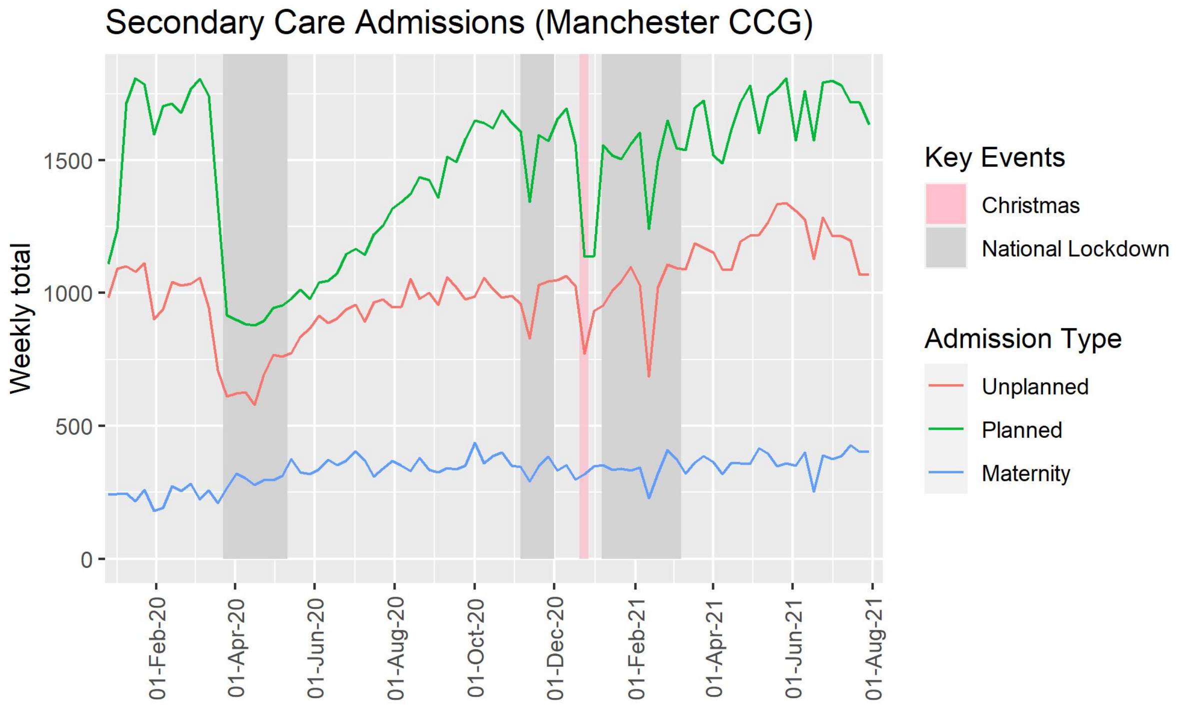

Supplementary Figure S4: Secondary healthcare utilisation across Manchester CCG, from January 2020 until August 2021.

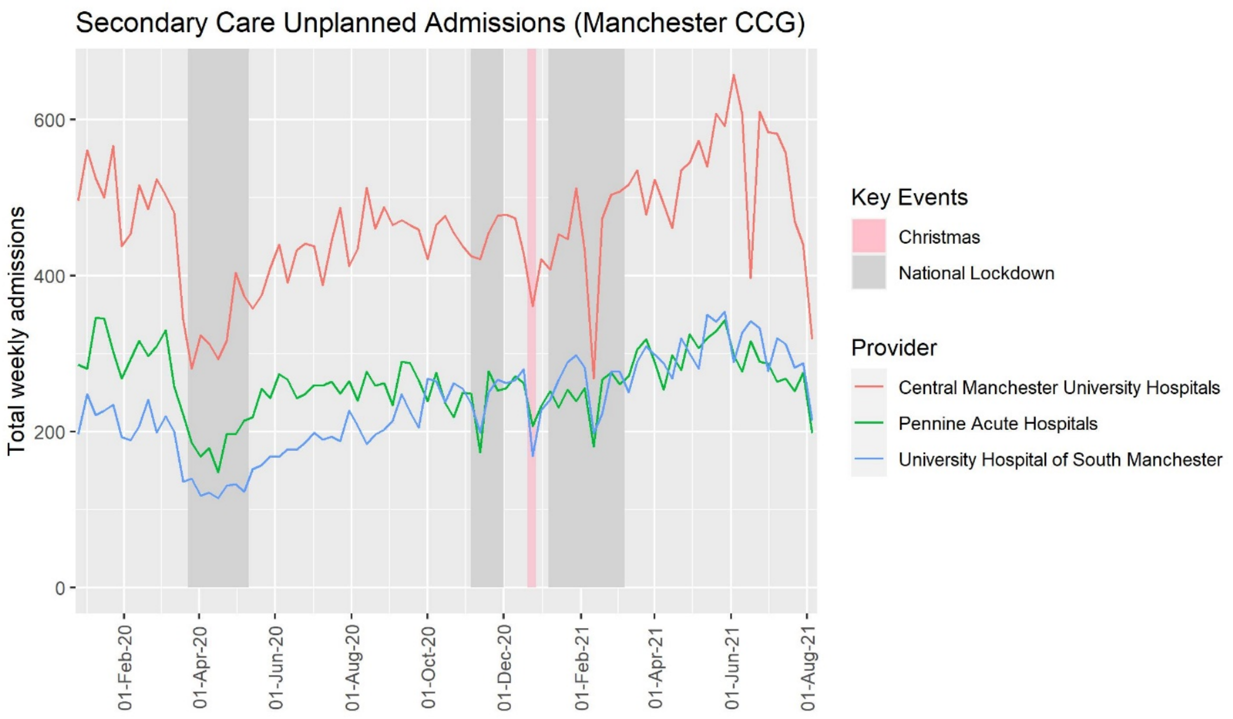

Supplementary Figure S5: Total weekly unplanned admissions of MCCG population at the three main providers (local to 96.6% of people) between January 2020 and August 2021.

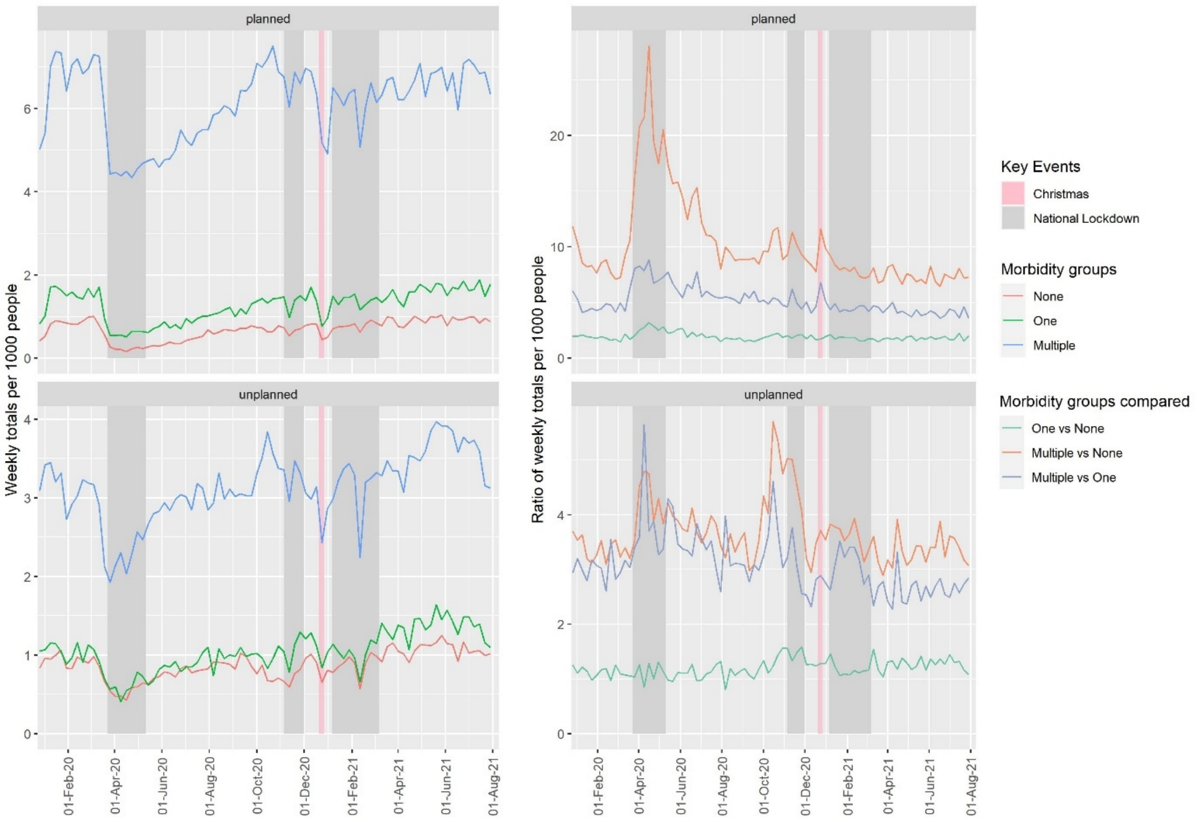

Supplementary Figure S6: Weekly planned and unplanned admissions per 1000 people within morbidity group in the MCCG population and the ratio between these scaled rates, between January 2020 and August 2021.

Supplementary Table S11: Estimates of the ratios of planned and unplanned admission rates for each LTC group in the final four weeks of the study compared to pre-pandemic rates.

	Planned			Unplanned		
LTC	Rate Ratio	95% CI	p-value	Rate Ratio	95% CI	p-value
Cancer	0.653	0.586 – 0.726	**<0.001**	0.926	0.773 – 1.105	0.401
Cardiovascular	0.938	0.901 – 0.977	**0.002**	0.978	0.915 – 1.044	0.502
Endocrine	0.997	0.951 – 1.044	0.893	1.000	0.930 – 1.076	0.990
Gastrointestinal	0.959	0.921 – 0.998	**0.039**	1.104	1.039 – 1.171	**0.001**
Musculoskeletal or Skin	1.025	0.974 – 1.078	0.340	1.093	1.017 – 1.173	**0.014**
Neurological	1.053	0.908 – 1.218	0.489	1.137	0.946 – 1.360	0.165
Psychiatric	1.006	0.959 – 1.054	0.814	1.113	1.049 – 1.181	**<0.001**
Renal or Urological	0.926	0.885 – 0.969	**0.001**	0.839	0.750 – 0.938	**0.002**
Respiratory	0.966	0.910 – 1.025	0.255	1.020	0.947 – 1.098	0.597
Sensory Impairment or Learning Disability	0.894	0.841 – 0.950	**<0.001**	1.016	0.926 – 1.113	0.736
Substance Abuse	1.196	1.074 – 1.329	**0.001**	1.139	1.013 – 1.278	**0.028**

For peer review only

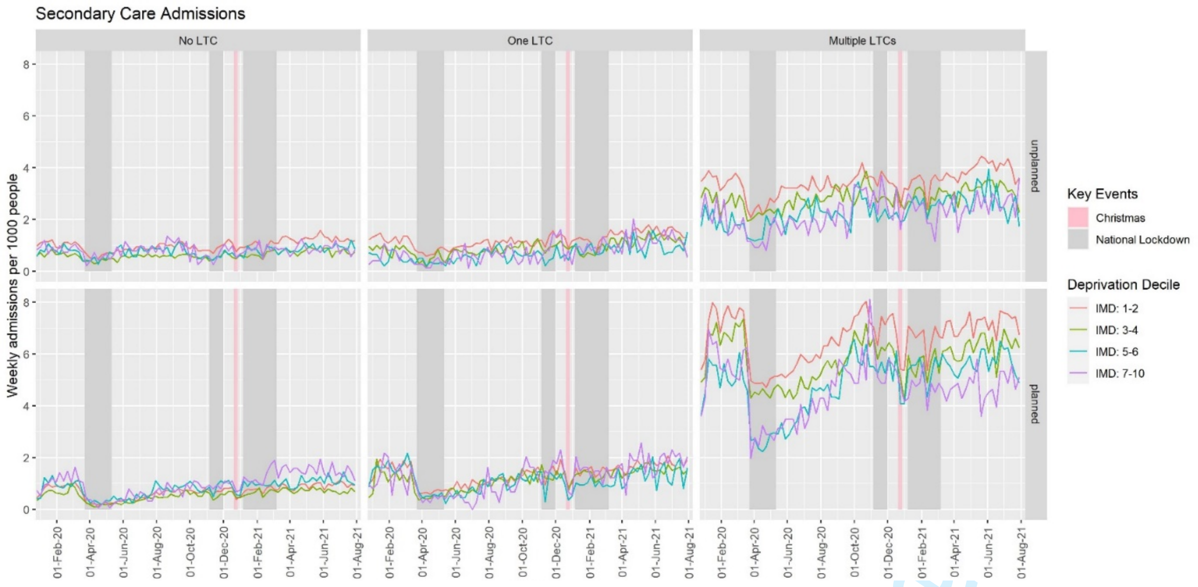

Supplementary Figure S7: Rates of weekly planned and unplanned admissions by deprivation and number of long-term conditions of the Manchester CCG population, between January 2020 and August 2021.

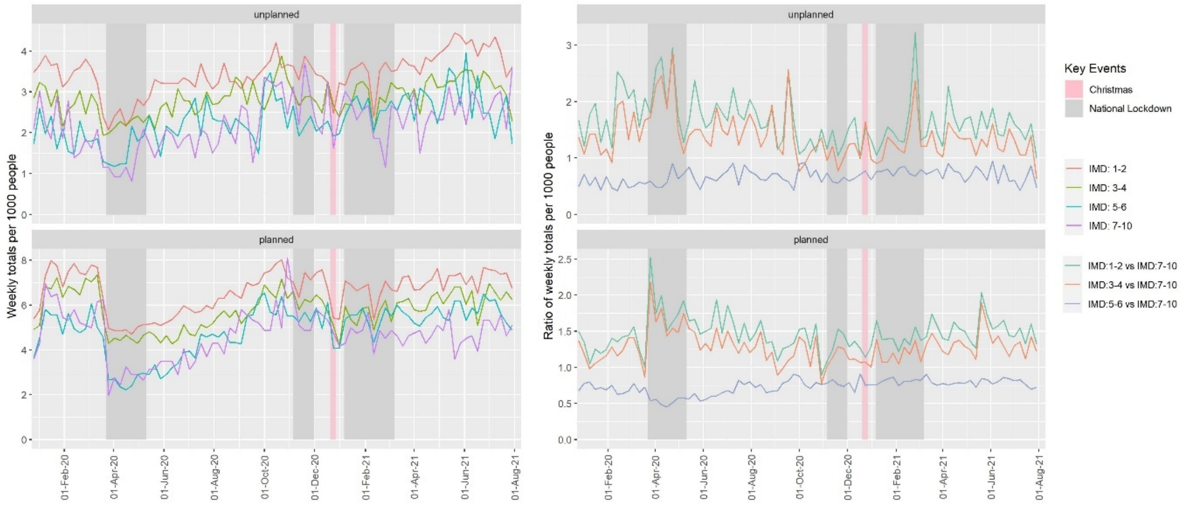

Supplementary Figure S8: Weekly planned and unplanned secondary care admissions per 1000 people within multi-morbid patients by deprivation group and the ratio of these compared to the least-deprived group (IMD: 7-10), between January 2020 and August 2021.

For peer review only

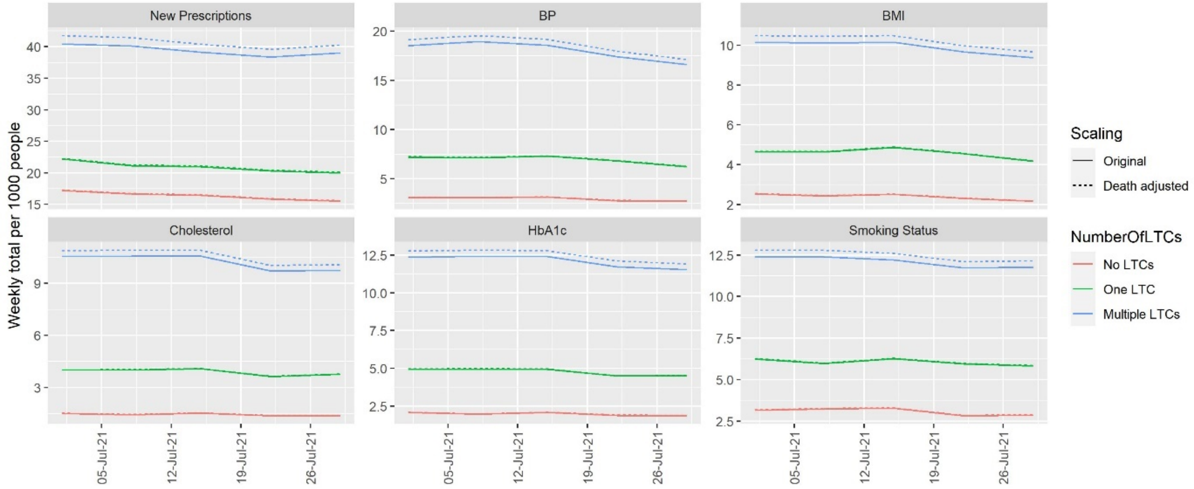

Supplementary Figure S9: Comparison between unadjusted (original) and death-adjusted weekly primary HCU measures per 1000 people, by morbidity in July 2021.

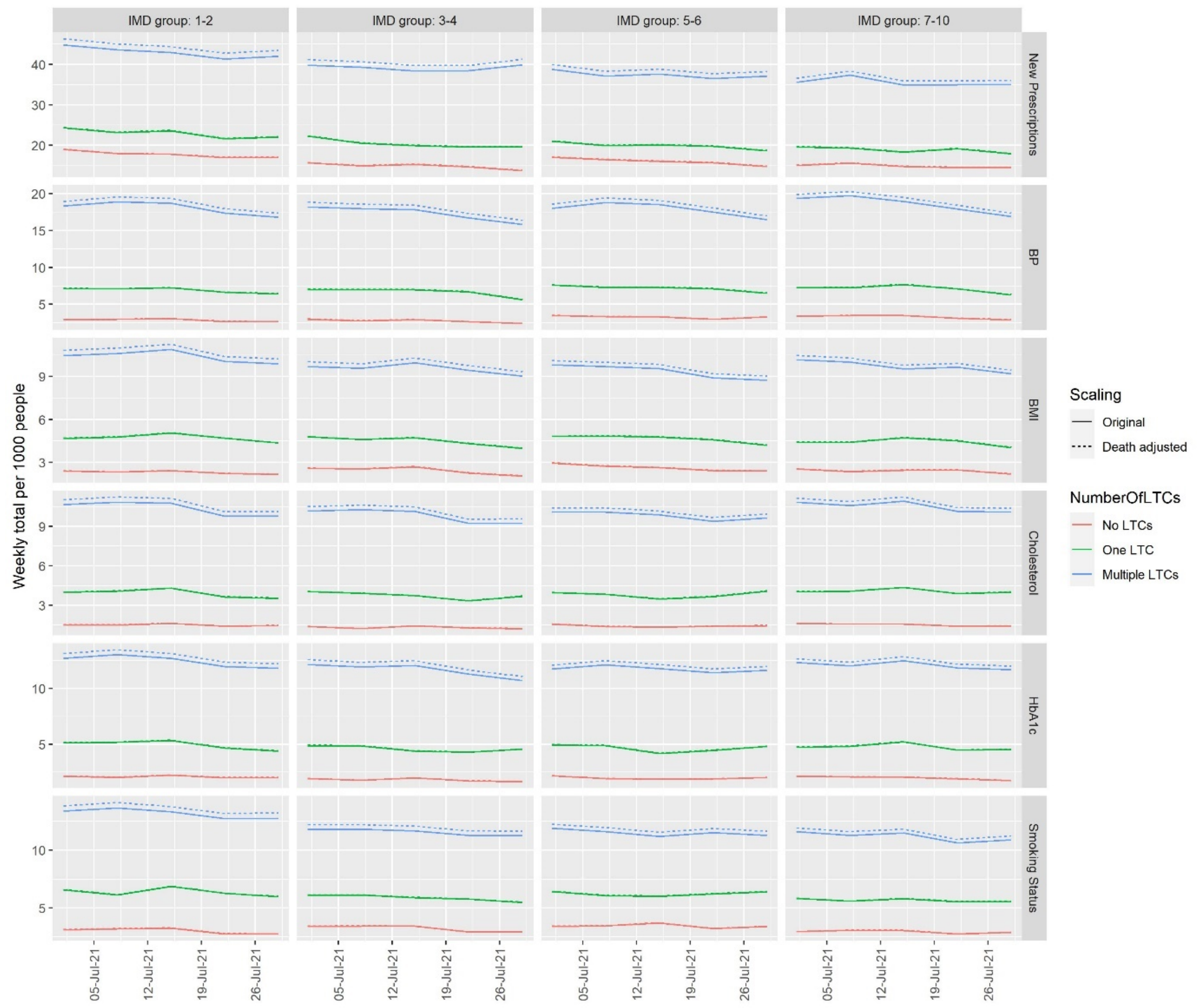

Supplementary Figure 10: Comparison between unadjusted (original) and death-adjusted weekly primary HCU measures per 1000 people, by deprivation group in July 2021.

Supplementary Table S12: Comparison between unadjusted (original) and death-adjusted weekly secondary care admissions per 1000 people, by morbidity group in July 2021.

Number of LTCs (morbidity group)	Admission Type	Week start	Unadjusted Weekly admissions per 1000 people	Death-Adjusted Weekly admissions per 1000 people	Difference
None	unplanned	01/07/2021	1.0235	1.0258	0.0023
None	unplanned	08/07/2021	1.0460	1.0483	0.0024
None	unplanned	15/07/2021	1.0560	1.0584	0.0024
None	unplanned	22/07/2021	0.9934	0.9957	0.0022
None	unplanned	29/07/2021	1.0185	1.0208	0.0023
One	unplanned	01/07/2021	1.4832	1.4898	0.0066
One	unplanned	08/07/2021	1.3576	1.3637	0.0060
One	unplanned	15/07/2021	1.3969	1.4031	0.0062
One	unplanned	22/07/2021	1.1536	1.1587	0.0051
One	unplanned	29/07/2021	1.0987	1.1035	0.0049
Multiple	unplanned	01/07/2021	3.6952	3.8118	0.1166
Multiple	unplanned	08/07/2021	3.7372	3.8551	0.1179
Multiple	unplanned	15/07/2021	3.5932	3.7066	0.1134
Multiple	unplanned	22/07/2021	3.1553	3.2548	0.0995
Multiple	unplanned	29/07/2021	3.1253	3.2239	0.0986
None	planned	01/07/2021	0.9859	0.9882	0.0022
None	planned	08/07/2021	0.9884	0.9907	0.0022
None	planned	15/07/2021	0.8458	0.8477	0.0019
None	planned	22/07/2021	0.9559	0.9581	0.0021
None	planned	29/07/2021	0.8733	0.8753	0.0020
One	planned	01/07/2021	1.6244	1.6317	0.0072

For peer review only

One	planned	08/07/2021	1.6558	1.6632	0.0073
One	planned	15/07/2021	1.8834	1.8918	0.0084
One	planned	22/07/2021	1.4832	1.4898	0.0066
One	planned	29/07/2021	1.7736	1.7814	0.0079
Multiple	planned	01/07/2021	7.1864	7.4131	0.2267
Multiple	planned	08/07/2021	7.0485	7.2708	0.2224
Multiple	planned	15/07/2021	6.8385	7.0542	0.2157
Multiple	planned	22/07/2021	6.8745	7.0914	0.2169
Multiple	planned	29/07/2021	6.3526	6.5530	0.2004

For peer review only

The RECORD statement – checklist of items, extended from the STROBE statement, that should be reported in observational studies using routinely collected health data.

	Item No.	STROBE items	Location in manuscript where items are reported	RECORD items	Location in manuscript where items are reported
**Title and abstract**					
	1	(a) Indicate the study's design with a commonly used term in the title or the abstract (b) Provide in the abstract an informative and balanced summary of what was done and what was found		RECORD 1.1: The type of data used should be specified in the title or abstract. When possible, the name of the databases used should be included.<br><br>RECORD 1.2: If applicable, the geographic region and timeframe within which the study took place should be reported in the title or abstract.<br><br>RECORD 1.3: If linkage between databases was conducted for the study, this should be clearly stated in the title or abstract.	1.1 Data and database specified in the abstract methods (Page 2)<br><br>1.2 Geographic region is specified in the title (Page 1), the timeframe is specified in the abstract methods. (Page 2)<br><br>1.3 No linkage between databases was conducted
**Introduction**					
Background rationale	2	Explain the scientific background and rationale for the investigation being reported			Introduction paragraphs 1-4 (Page 3)
Objectives	3	State specific objectives, including any prespecified hypotheses			Introduction paragraph 4 (Page 3)
**Methods**					
Study Design	4	Present key elements of study design early in the paper			Methods: Design and Data Source (Page 3)
Setting	5	Describe the setting, locations, and relevant dates, including			Methods:

For peer review only

		periods of recruitment, exposure, follow-up, and data collection			Design and Data Source<br>Study Populations and Key Time Points<br>(Pages 3 and 4)
Participants	6	*(a) Cohort study* - Give the eligibility criteria, and the sources and methods of selection of participants. Describe methods of follow-up<br>*Case-control study* - Give the eligibility criteria, and the sources and methods of case ascertainment and control selection. Give the rationale for the choice of cases and controls<br>*Cross-sectional study* - Give the eligibility criteria, and the sources and methods of selection of participants<br><br>*(b) Cohort study* - For matched studies, give matching criteria and number of exposed and unexposed<br>*Case-control study* - For matched studies, give matching criteria and the number of controls per case		RECORD 6.1: The methods of study population selection (such as codes or algorithms used to identify subjects) should be listed in detail. If this is not possible, an explanation should be provided.<br><br>RECORD 6.2: Any validation studies of the codes or algorithms used to select the population should be referenced. If validation was conducted for this study and not published elsewhere, detailed methods and results should be provided.<br><br>RECORD 6.3: If the study involved linkage of databases, consider use of a flow diagram or other graphical display to demonstrate the data linkage process, including the number of individuals with linked data at each stage.	Methods:<br>Long-term medical conditions<br>Index of Multiple Deprivation<br>(Pages 4 and 5)
Variables	7	Clearly define all outcomes, exposures, predictors, potential confounders, and effect modifiers. Give diagnostic criteria, if applicable.		RECORD 7.1: A complete list of codes and algorithms used to classify exposures, outcomes, confounders, and effect modifiers should be provided. If these cannot be reported, an explanation should be provided.	Methods:<br>Long-term medical conditions<br>Index of Multiple Deprivation<br>Measuring Healthcare

For peer review only

					Utilisation (Pages 4 and 5)
Data sources/ measurement	8	For each variable of interest, give sources of data and details of methods of assessment (measurement). Describe comparability of assessment methods if there is more than one group			Measuring Healthcare Utilisation (Page 5)
Bias	9	Describe any efforts to address potential sources of bias			NA
Study size	10	Explain how the study size was arrived at			Study populations and key time points (Page 4)
Quantitative variables	11	Explain how quantitative variables were handled in the analyses. If applicable, describe which groupings were chosen, and why			Methods: Statistical Analysis (Pages 5 and 6)
Statistical methods	12	(a) Describe all statistical methods, including those used to control for confounding<br>(b) Describe any methods used to examine subgroups and interactions<br>(c) Explain how missing data were addressed<br>(d) *Cohort study* - If applicable, explain how loss to follow-up was addressed<br>*Case-control study* - If applicable, explain how matching of cases and controls was addressed<br>*Cross-sectional study* - If applicable, describe analytical methods taking account of sampling strategy			Methods: Statistical Analysis (Pages 5 and 6)

For peer review only

		(e) Describe any sensitivity analyses			
Data access and cleaning methods		..		RECORD 12.1: Authors should describe the extent to which the investigators had access to the database population used to create the study population.<br><br>RECORD 12.2: Authors should provide information on the data cleaning methods used in the study.	12.1 Methods: Design and Data Source (Page 3)<br><br>12.2 NA
Linkage		..		RECORD 12.3: State whether the study included person-level, institutional-level, or other data linkage across two or more databases. The methods of linkage and methods of linkage quality evaluation should be provided.	NA
**Results**					
Participants	13	(a) Report the numbers of individuals at each stage of the study (*e.g.*, numbers potentially eligible, examined for eligibility, confirmed eligible, included in the study, completing follow-up, and analysed)<br>(b) Give reasons for non-participation at each stage.<br>(c) Consider use of a flow diagram		RECORD 13.1: Describe in detail the selection of the persons included in the study (*i.e.,* study population selection) including filtering based on data quality, data availability and linkage. The selection of included persons can be described in the text and/or by means of the study flow diagram.	NA
Descriptive data	14	(a) Give characteristics of study participants (*e.g.*, demographic, clinical, social) and information on exposures and potential confounders<br>(b) Indicate the number of participants with missing data for each variable of interest			Results paragraph 1 (Page 6)

For peer review only

		(c) *Cohort study* - summarise follow-up time (*e.g.*, average and total amount)			
Outcome data	15	*Cohort study* - Report numbers of outcome events or summary measures over time<br>*Case-control study* - Report numbers in each exposure category, or summary measures of exposure<br>*Cross-sectional study* - Report numbers of outcome events or summary measures			Results (Pages 6 – 10)
Main results	16	(a) Give unadjusted estimates and, if applicable, confounder-adjusted estimates and their precision (e.g., 95% confidence interval). Make clear which confounders were adjusted for and why they were included<br>(b) Report category boundaries when continuous variables were categorized<br>(c) If relevant, consider translating estimates of relative risk into absolute risk for a meaningful time period			Results (Pages 6 – 10)
Other analyses	17	Report other analyses done—e.g., analyses of subgroups and interactions, and sensitivity analyses			Supplementary materials
**Discussion**					
Key results	18	Summarise key results with reference to study objectives			Discussion paragraph 1 (Page 10)
Limitations	19	Discuss limitations of the study, taking into account sources of potential bias or imprecision.		RECORD 19.1: Discuss the implications of using data that were not created or collected to answer the	Discussion Strengths &

For peer review only

		Discuss both direction and magnitude of any potential bias		specific research question(s). Include discussion of misclassification bias, unmeasured confounding, missing data, and changing eligibility over time, as they pertain to the study being reported.	Limitations (Page 12)
Interpretation	20	Give a cautious overall interpretation of results considering objectives, limitations, multiplicity of analyses, results from similar studies, and other relevant evidence			Discussion (Pages 10-12)
Generalisability	21	Discuss the generalisability (external validity) of the study results			Discussion Strengths & Limitations (Page 12)
**Other Information**					
Funding	22	Give the source of funding and the role of the funders for the present study and, if applicable, for the original study on which the present article is based			Page 13
Accessibility of protocol, raw data, and programming code		..		RECORD 22.1: Authors should provide information on how to access any supplemental information such as the study protocol, raw data, or programming code.	Page 13

*Reference: Benchimol EI, Smeeth L, Guttmann A, Harron K, Moher D, Petersen I, Sørensen HT, von Elm E, Langan SM, the RECORD Working Committee. The REporting of studies Conducted using Observational Routinely-collected health Data (RECORD) Statement. *PLoS Medicine* 2015; in press.

*Checklist is protected under Creative Commons Attribution (CC BY) license.

For peer review only - http://bmjopen.bmj.com/site/about/guidelines.xhtml

BMJ Open

Healthcare utilisation in patients with long-term conditions during the COVID-19 pandemic: a population-based observational study of all patients across Greater Manchester, UK

Journal:	*BMJ Open*
Manuscript ID	bmjopen-2022-066873.R1
Article Type:	Original research
Date Submitted by the Author:	05-Feb-2023
Complete List of Authors:	Sammut-Powell, Camilla; The University of Manchester Faculty of Biology Medicine and Health, Division of Informatics, Imaging and Data Science; The University of Manchester Faculty of Biology Medicine and Health, National Institute for Health Research Applied Research Collaboration Greater Manchester<br>Williams, Richard; The University of Manchester Faculty of Biology Medicine and Health, Division of Informatics, Imaging and Data Science; The University of Manchester Faculty of Biology Medicine and Health, National Institute for Health Research Greater Manchester Patient Safety Translational Research Centre<br>Sperrin, Matthew; The University of Manchester Faculty of Biology Medicine and Health, Division of Informatics, Imaging and Data Science<br>Thomas, Owain; Langworthy Medical Practice<br>Peek, N; The University of Manchester Faculty of Biology Medicine and Health, Division of Informatics, Imaging, and Data Science; The University of Manchester Faculty of Biology Medicine and Health, National Institute for Health Research Applied Research Collaboration Greater Manchester<br>Grant, Stuart; The University of Manchester Division of Cardiovascular Sciences, ERC, Manchester University Hospitals Foundation Trust
**Primary Subject Heading**:	Epidemiology
Secondary Subject Heading:	General practice / Family practice, Health services research
Keywords:	COVID-19, Epidemiology < TROPICAL MEDICINE, Public health < INFECTIOUS DISEASES

SCHOLARONE™
Manuscripts

I, the Submitting Author has the right to grant and does grant on behalf of all authors of the Work (as defined in the below author licence), an exclusive licence and/or a non-exclusive licence for contributions from authors who are: i) UK Crown employees; ii) where BMJ has agreed a CC-BY licence shall apply, and/or iii) in accordance with the terms applicable for US Federal Government officers or employees acting as part of their official duties; on a worldwide, perpetual, irrevocable, royalty-free basis to BMJ Publishing Group Ltd ("BMJ") its licensees and where the relevant Journal is co-owned by BMJ to the co-owners of the Journal, to publish the Work in this journal and any other BMJ products and to exploit all rights, as set out in our licence.

The Submitting Author accepts and understands that any supply made under these terms is made by BMJ to the Submitting Author unless you are acting as an employee on behalf of your employer or a postgraduate student of an affiliated institution which is paying any applicable article publishing charge ("APC") for Open Access articles. Where the Submitting Author wishes to make the Work available on an Open Access basis (and intends to pay the relevant APC), the terms of reuse of such Open Access shall be governed by a Creative Commons licence – details of these licences and which Creative Commons licence will apply to this Work are set out in our licence referred to above.

Other than as permitted in any relevant BMJ Author's Self Archiving Policies, I confirm this Work has not been accepted for publication elsewhere, is not being considered for publication elsewhere and does not duplicate material already published. I confirm all authors consent to publication of this Work and authorise the granting of this licence.

For peer review only

Healthcare utilisation in patients with long-term conditions during the COVID-19 pandemic: a population-based observational study of all patients across Greater Manchester, UK

Camilla Sammut-Powell[1,2], Richard Williams[1,2,3], Matthew Sperrin[1], Owain Thomas[4], Niels Peek [1,2,3,5], Stuart W. Grant[6]

1. Division of Informatics, Imaging and Data Science, Faculty of Biology, Medicine and Health, University of Manchester, Manchester, M13 9PL, UK
2. National Institute for Health Research Applied Research Collaboration Greater Manchester, Faculty of Biology, Medicine and Health, University of Manchester, Manchester, M13 9PL, UK
3. National Institute for Health Research Greater Manchester Patient Safety Translational Research Centre, Faculty of Biology, Medicine and Health, University of Manchester, Manchester, M13 9PL, UK
4. Langworthy Medical Practice, Salford, M6 5WW, UK
5. National Institute for Health Research Manchester Biomedical Research Centre, Faculty of Biology, Medicine and Health, University of Manchester, Manchester, M13 9PL, UK
6. Division of Cardiovascular Sciences, University of Manchester, ERC, Manchester University Hospitals Foundation Trust, Manchester, M23 9LT, UK.

Corresponding Author: Prof. Niels Peek, Division of Informatics, Imaging and Data Science, Faculty of Biology, Medicine and Health, University of Manchester, Manchester, M13 9PL, UK.

Corresponding Author's Email: niels.peek@manchester.ac.uk

For peer review only

ABSTRACT

Objectives: Data on population healthcare utilisation (HCU) across both primary and secondary care during the COVID-19 pandemic are lacking. We describe primary and secondary HCU stratified by long-term conditions (LTCs) and deprivation, during the first 19 months of COVID-19 pandemic across a large urban area in the United Kingdom.

Design: A retrospective, observational study.

Setting: All primary and secondary care that contributed to the Greater Manchester Care Record throughout 30th-December-2019 to 1st-August-2021.

Participants: 3,225,169 patients were registered with or attended an NHS primary or secondary care service.

Primary outcomes: Primary care HCU (incident prescribing and recording of healthcare information) and secondary care HCU (planned and unplanned admissions) were assessed.

Results: The first national lockdown was associated with reductions in all primary HCU measures, ranging from 24.7% (24.0% to 25.5%) for incident prescribing to 84.9% (84.2% to 85.5%) for cholesterol monitoring. Secondary HCU also dropped significantly for planned (47% (42.9% to 51.5%)) and unplanned admissions (35.0% (28.3% to 41.6%)). Only secondary care had significant reductions in HCU during the second national lockdown. Primary HCU measures had not recovered to pre-pandemic levels by the end of the study. The secondary admission rate ratio between multi-morbid patients and those without LTCs increased during the first lockdown by a factor of 2.4 (2.0 to 2.9;p<0.001) for planned admissions and 1.3 (1.1 to 1.5;p=0.006) for unplanned admissions. No significant changes in this ratio were observed in primary HCU.

Conclusion: Major changes in primary and secondary HCU have been observed during the COVID-19 pandemic. Secondary HCU reduced more in those without LTCs and the ratio of utilisation between patients from the most and least deprived areas increased for the majority of HCU measures. Overall primary HCU and secondary care HCU for some LTC groups had not returned to pre-pandemic levels by the end of the study.

For peer review only

STRENGTHS AND LIMITATIONS OF THIS STUDY

Strengths

- This study includes data on over 3 million individuals, representing all patients registered with a general practitioner across an entire geographical area.
- Both primary and secondary care services were analysed in this study.
- Five surrogate markers of health care utilisation were considered in the primary care analyses.

Limitations

- Historical data prior to the start of the COVID-19 pandemic were not available which limited the trend analyses that could be performed.
- Data from secondary care providers was limited to only a subset of the population.

For peer review only

INTRODUCTION

On the 30th of January 2020, the World Health Organization (WHO) declared a public health emergency of international concern with governments urged to prepare for global spread of COVID-19.[1] With case numbers increasing and the virus spreading globally, COVID-19 was characterised as a pandemic six weeks later and rapidly developed into a global public health emergency. As of the 17th of December 2021 approximately 273 million cases and 5.3 million COVID-19 associated deaths have been reported globally.[2]Governments across the world enacted a range of measures aimed at controlling the spread of the virus,[3] and increasing healthcare capacity.[4,5] Despite these measures healthcare systems have been overwhelmed and diversion of healthcare resources to address increased demand specific to COVID-19 has been required.[6,7] The impact of this diversion of resources on the care of patients with non COVID-19 illnesses has been exacerbated by reduced staff availability due to COVID-19 infection amongst healthcare workers.[8]

Numerous studies have been undertaken to assess the impact of the pandemic on healthcare provision in a variety of settings. An analysis of UK general practitioner (GP) data demonstrated that diagnoses of common physical and mental health conditions decreased substantially early in the pandemic.[9] The number of urgent GP referrals for cancer fell by 60% in April 2020 compared to the same month in 2019.[10] Hospital administrative data has demonstrated a decline in patients presenting with acute coronary syndrome from mid-February 2020 onwards, [11] and a separate analysis demonstrated a 43% reduction in patients undergoing percutaneous coronary interventions for ST-elevation myocardial infarctions compared to previous years.[12] Modelling studies have suggested that approximately 28,000,000 elective surgical procedures were cancelled over a 12-week period of peak disruption caused by the pandemic.[13]

Most studies to date investigating the impact of the pandemic on healthcare utilisation (HCU) have assessed specific patient groups, largely focussed on secondary care.[14] Changes to HCU during the COVID-19 pandemic for both primary and secondary care stratified across the range of long-term medical conditions (LTCs) and different levels of social deprivation have not previously been described. The Greater Manchester Care Record (GMCR) includes electronic health records from all primary and secondary care National Health Service (NHS) providers in the metropolitan county of Greater Manchester (GM). GM has been significantly affected by COVID-19,[15] and the GMCR provides a unique opportunity to study the impact of the pandemic on primary and secondary HCU in patients with LTCs across this defined urban area.

For peer review only - http://bmjopen.bmj.com/site/about/guidelines.xhtml

METHODS

Design and Data Source

This was a retrospective, observational, service evaluation using routinely collected data. The data analysed were from two sources: 1) HCU data from the GMCR which is an integrated patient record containing data from primary and secondary NHS services across Greater Manchester (GM) and 2) contextual Government COVID-19 data [16] regarding the number of new COVID-19 cases and COVID-19 related hospital admissions.

Greater Manchester Care Record

The GMCR is populated with data from primary care (General Practitioners), secondary care (acute and community hospitals), mental health trusts and social care across an entire geographical region. A total of nine secondary care organisations (including 12 hospitals), 3 mental health trusts and 10 clinical commissioning groups (CCGs) contribute data. The primary purpose of the GMCR is for direct patient care as it provides clinicians with information from other health care providers relevant to their patient encounters that would ordinarily be inaccessible. However, it has also been made available in de-identified format for research relating to COVID-19.

UK Government COVID-19 Data

Data regarding the number of new COVID-19 cases and COVID-19 related hospital admissions were collected by the UK government throughout the pandemic. The number of new cases by specimen date was extracted for Manchester and the number of COVID-19 admissions were extracted for each of the secondary acute providers serving the people with a Manchester CCG (MCCG), included within the GMCR. The data is freely available from https://coronavirus.data.gov.uk/details/download and full details of the data extraction are provided in the Supplementary Materials (Supplementary Table S1).

Data processing and approvals

All identifiable data including free text are redacted. Some non-identifying demographic data are available such as recorded gender, year of birth, lower layer super output area (LSOA), index of multiple deprivation and ethnicity. The University of Manchester is permitted to perform research on this data via a Greater Manchester wide data protection impact assessment (DPIA). The basis for this DPIA is the control of patient information (COPI) notice issued by the Secretary of State for Health and Social Care in March 2020 which allowed confidential patient information to be shared for the purposes of research into COVID-19.[17] The study was approved by both the GMCR Expert Review Group and Research Governance Group. All data made available to the analysts was de-identified and aggregated and therefore did not require specific ethical approval.

Study populations and key time points

The main study population consisted of all patients that were registered with a GP within GM on 1st January 2020, defined as the GM population. The 1st January 2020 is the index study date. For the primary care analyses the entire GM population were considered. However, secondary care data were only available for patients registered to a Manchester CCG, hence, the secondary healthcare utilisation analyses were limited to these people. The dates of the national lockdowns initiated in response to the COVID-19 pandemic were indicated in addition to Christmas week due to expected changes in HCU during these periods. The first national lockdown ran from the 23rd March 2020 to

For peer review only - http://bmjopen.bmj.com/site/about/guidelines.xhtml

the 11th May 2020, the second national lockdown ran from 5th November 2020 to 1st December 2020 and the third national lockdown ran from 6th January 2021 to the 8th March 2021.

Long-term medical conditions

Long-term medical conditions (LTCs) were defined as per Barnett et al,[18] and were grouped into the following categories: cancer, cardiovascular, endocrine, gastrointestinal, musculoskeletal or skin, neurological, psychiatric, renal or urological, respiratory, sensory impairment or learning disability, and substance abuse. A resident was identified as being diagnosed with a LTC by interrogating the GMCR record prior to the index date. If a long-term medical condition was diagnosed after the 1st January 2020 the patient was not recorded as having the LTC for this analysis. People that were identified as belonging to multiple LTC groups were assigned to each corresponding LTC group and defined as multi-morbid. The full list of long-term conditions and groupings are provided in Supplementary Table S2.

Index of Multiple Deprivation

The 2019 Index of Multiple Deprivation (IMD) is the official measure of relative deprivation provided by the Office for National Statistics which combines information from seven different domains to produce an overall relative measure of deprivation for each LSOA. Each LSOA is ranked from least to most deprived, and deciles of relative deprivation are generated.[19] For this study the available IMD deciles were categorised into four groups, representing the most deprived (deciles 1-2), highly deprived (deciles 3-4), moderately deprived (deciles 5-6) and the least deprived LSOAs (deciles 7-10). The least deprived LSOA group consisted of 4 deciles to avoid multiple small groups because of the skew towards more deprived LSOA deciles in GM.

Measuring Healthcare Utilisation

For primary HCU, appointment data were not available within the GMCR. Surrogate markers of HCU were therefore evaluated consisting of first prescriptions and recording of healthcare information in the GP record. First prescriptions were identified by the issuing of any new prescription (non-repeat prescription) for an individual patient by a primary care healthcare professional and are referred to as incident prescriptions throughout the manuscript. This measure was selected as issuing of an incident prescription requires contact with a healthcare professional. Healthcare information recorded included: recoding of smoking status, measurements of cholesterol, blood pressure (BP), blood glucose (HbA1c) and body mass index (BMI). The values of these measurements were not used in the analysis.

For secondary HCU, the number of acute provider admissions were evaluated. To enable population admission rates to be calculated, a denominator was calculated by assigning each resident within Manchester CCG to a secondary care provider according to the most common provider observed within their LSOA. In cases where there the most common secondary provider was unclear one of the two most common providers was randomly assigned to that LSOA. Secondary care admissions were categorised into planned, unplanned, maternity, transfers and 'other' admission, defined according to the admission type field available in the provider data. Daily aggregate level data counts of all utilisation measures were provided. A full description of the data processing applied is available at https://github.com/rw251/gm-idcr/tree/master/projects/001%20-%20Grant.

For peer review only - http://bmjopen.bmj.com/site/about/guidelines.xhtml

Statistical Analysis

Weekly totals of HCU data were evaluated for the entire population. The rate ratios (RRs) of utilisation in the weeks before and after the initiation of the first and second national lockdowns (1st national lockdown: w/c 23rd March 2020 vs w/c 9th March 2020; 2nd national lockdown: w/c 9th November 2020 vs w/c 19th October 2020) were estimated across all measures of HCU to determine the association between the initiation of each national lockdown and HCU.

The effect of the initiation of the third national lockdown was not estimated since the weeks prior coincided with Christmas, where utilisation is expected to be reduced. The pre-pandemic weeks (prior to 9th March 2020) were compared against each of the national lockdown periods to determine if there was a significant change in the rates of HCU associated with each of the national lockdowns using Poisson regression. A Poisson regression model, linear in time, was fit to the weekly rates of utilisation after the initiation of the first national lockdown until the end of the study to determine the overall change of utilisation throughout the pandemic. A direct comparison between utilisation observed in a calendar week in 2021 vs 2020 was conducted for calendar weeks 2 to 11, using rate ratios; it was assumed that the data in calendar weeks 2 to 11 in 2020 were unaffected by the pandemic and consequently act as a control. Additionally, the rate ratios between utilisation measures in the final four weeks of the study and the pre-pandemic period were estimated using Poisson regression to compare how utilisation differed from pre-pandemic levels by the end of the study.

Subgroup analyses were performed across LTC and IMD groups. To compare subgroups, we further provided the rates of utilisation per 1000 people by dividing by the total number of people assigned to the corresponding subgroup and multiplying by 1000. For example, when comparing the rates across number of LTCs (none, one or multiple), for the people without any LTCs, the rate of weekly secondary care admissions is defined as the total number of admissions experienced by this subgroup within a given week divided by the total number of people within the subgroup, multiplied by 1000. The interactive effect between the each of the national lockdowns and subgroup HCU was estimated using log-linear regression.

A sensitivity analysis to adjust for deaths that occurred during the study was performed, where rates of utilisation were re-calculated in July 2021 dividing by the total numbers of patients that were still alive (death-adjusted), and compared to the un-adjusted rates. July 2021 was chosen since this was one month before the end of the study and therefore captured the majority of deaths that occurred throughout the study; hence if no difference was observed between the death-adjusted and un-adjusted rates, the un-adjusted rates would pertain across all weeks. All analyses were performed in R version 4.0.0, using the packages 'tidyverse'[20], 'scales'[21], 'reshape2'[22] and 'cowplot'[23].

Patient and public involvement

Two public representatives provided input throughout the project. Both representatives gave their full support to the proposed project and are preparing a patient and public summary of the research for dissemination.

RESULTS

The total population captured within the GMCR includes 3,225,169 patients, of whom 693,749 were registered with a Manchester CCG. The mean age of the population was 38.2 years old (SD 22.8),

For peer review only - http://bmjopen.bmj.com/site/about/guidelines.xhtml

with 49.0% of the population registered as female. The majority of the population (64.5%) had a registered ethnicity as white. Asian or British Asian patients represented 11.5% of the population and Black or Black British patients represented 4.0%. Ethnicity data were not available for 12.0% of patients. The prevalence of LTCs is shown in Table 1. The most common LTCs observed were psychiatric (GMCR: 26.5%, MCCG: 20.7%), cardiovascular (GMCR: 17.4%, MCCG: 10.5%), respiratory (GMCR: 17.1%, MCCG: 13.4%) and gastrointestinal (GMCR: 15.5%, MCCG: 11.8%). Levels of deprivation were high, with 41.4% of the GM population and the majority of those registered within Manchester CCG (58.5%) residing in areas that are in the most deprived quintile (decile of 1 or 2; Table 1).

Overall primary HCU

There was a rapid decrease in all weekly primary HCU measures starting just prior to the first national lockdown (Figure 1). The largest drops in activity associated with the initiation of the first national lockdown were for recording of healthcare information (% drop (95% CI): BP: 82.4% (82.0%-82.9%); BMI: 79.5% (78.8%-80.1%); Cholesterol 84.9% (84.2%-85.5%); HbA1c 84.0% (83.3%-84.6%); Smoking status 62.2% (61.3%-63.1%). There was still a significant drop in the new prescriptions but the change was proportionally smaller (24.7%; 95% CI: 24.0%-25.5%). These reductions were sustained throughout the first national lockdown (Supplementary Table S3). The initiation of the second national lockdown was associated with an increase or no significant change in primary HCU (% increase (95%CI): new prescriptions: -0.3% (-1.3%-0.6%); BP: 4.1% (2.2%-6.1%); BMI: 2.4% (0.0%-4.8%); Cholesterol: 10.0% (7.3%-12.8%); HbA1c: 6.5% (4.1%-8.9%); Smoking status: -0.2% (-2.1%-1.8%)).

Table 1: Long-term conditions and social deprivation identified in the GM population and the Manchester CCG subpopulation.

	Greater Manchester (N=3,225,169)	%	Manchester CCG (N=693,749)	%
**LTC Group***				
Cancer	50954	1.6	6307	0.9
Cardiovascular	561195	17.4	72912	10.5
Endocrine	393274	12.2	64557	9.3
Gastrointestinal	501060	15.5	81957	11.8
Musculoskeletal or Skin	284103	8.8	52593	7.6
Neurological	46672	1.4	7340	1.1
Psychiatric	854454	26.5	143707	20.7
Renal or Urological	130436	4.0	16617	2.4
Respiratory	550648	17.1	93174	13.4
Sensory Impairment or Learning Disability	314264	9.7	45909	6.6
Substance Abuse	115532	3.6	24068	3.5
**Number of LTCs**				
None	1530501	47.5	399618	57.6
One	631648	19.6	127428	18.4
Multiple	1063020	33.0	166703	24.0
**IMD Group**				
1 to 2	1335061	41.4	405862	58.5

For peer review only - http://bmjopen.bmj.com/site/about/guidelines.xhtml

3 to 4	675296	20.9	185118	26.7
5 to 6	424139	13.2	67192	9.7
7 to 10	788553	24.4	35087	5.1
Missing	2120	0.1	490	0.1

LTC – long term condition; IMD – index of multiple deprivation. *These data represent the overall prevalence of each long term condition in the population. Each individual can be represented in more than one LTC row.

All primary care HCU increased with time from the initiation of the first national lockdown to the end of the study (annual RR (95%CI): new prescriptions: 1.189 (1.186 - 1.191; p<0.001); BP: 2.113 (2.104 – 2.122;p<0.001); BMI: 2.097 (2.086 – 2.108;p<0.001); Cholesterol: 2.221 (2.209 – 2.234;p<0.001); HbA1c: 2.165 (2.153 – 2.176;p<0.001); Smoking status: 1.538 (1.531 – 1.544;p<0.001)). Despite this, by the end of the study, all measures were still recorded less often than in the pre-pandemic period (RR (95% CI; p-value); new prescriptions: 0.828 (0.825 – 0.832; p<0.001); BP: 0.583 (0.580-0.587; p<0.001); BMI: 0.669 (0.664-0.675; p<0.001); cholesterol: 0.896 (0.888-0.905; p<0.001); HbA1c: 0.935 (0.927-0.943; p<0.001); smoking status: 0.711 (0.706-0.717; p<0.001)).

All primary care measures were lower across calendar weeks 2-11 when comparing 2021 data with 2020 data (p<0.001; Supplementary Table S4). The measuring of BP and BMI remained consistently lower throughout these weeks by an average of 50.0% (95%CI: 49.8%-50.2%; p<0.001) and 42.5% (95% CI: 42.1%-42.8%; p<0.001), respectively. Even though the rates of cholesterol and HbA1c measurements taken in calendar week 11 were similar in 2021 (pandemic) and 2020 (pre-pandemic): 0.937 (95% CI: 0.916 – 0.958) and 0.973 (95% CI: 0.953 – 0.992), respectively, they were still significantly lower in 2021.

Primary HCU by multi-morbidity and deprivation

Multi-morbid patients and patients with one LTC had consistently higher levels of primary HCU than patients with no LTCs throughout the study period (Supplementary Table S5; Figure 2). The ratio of weekly HCU rates per 1000 people between multi-morbid patients and those with no LTCs significantly increased for new prescriptions (RR: 1.281; 95%CI: 1.169 – 1.404; p <0.001), BP (RR: 1.187; 95%CI: 1.007 – 1.400; p = 0.042) and smoking status (RR: 1.356; 95%CI: 1.126 – 1.632; p = 0.002) during the first national lockdown, and decreased for BMI (RR: 0.736; 95%CI 0.613 – 0.885; p=0.001) and smoking status (RR: 0.803; 95%CI 0.675 – 0.956; p=0.014) in the third national lockdown. No significant changes in HCU between multi-morbid patients and those with no LTCs were observed for HBA1c or cholesterol or during the second national lockdown for all primary care HCU (Supplementary Table S6; Supplementary Figure S1).

Primary HCU by deprivation

People that were from less deprived areas had lower rates of new prescriptions compared to those from the most deprived areas (IMD 1-2), (RR (95%CI); 3-4 vs 1-2: 0.915 (0.874 – 0.959); 5-6 vs 1-2: 0.920 (0.878 – 0.964); 7-10 vs 1-2: 0.875 (0.835 – 0.917); Figure 2, Supplementary Table S7). Similarly, smoking status had a lower rate of measurement in patients from the least deprived areas compared to patients from the most deprived areas (RR: 0.885; 95% CI: 0.801 – 0.877). No other differences were observed with regards to deprivation across primary HCU. The group from the least

For peer review only - http://bmjopen.bmj.com/site/about/guidelines.xhtml

deprived areas had experienced an additional reduction in smoking status during the third national lockdown (RR: 0.836; 95%CI 0.704 - 0.994; p=0.042) but no other interactions between deprivation and national lockdowns were evident (Supplementary Table S8).

Interaction between multi-morbidity and deprivation for primary HCU

Differences in HCU by deprivation were overall larger within multi-morbid patients (Supplementary Table S9; Figure 2). Differences in HCU between deprivation groups were not attributable to only one LTC group (Supplementary Figure S2). In multi-morbid patients, there were no significant changes in the ratio of weekly HCU per 1000 people between the group from the least deprived areas and the groups from other deprivation areas, across all primary HCU measures, during the first national lockdown compared to pre-pandemic weeks (Supplementary Table S10; Supplementary Figure S3).

Overall secondary HCU

There has been large variation in planned and unplanned secondary HCU over the course of the COVID-19 pandemic (Supplementary Figure S4). There was a 47.4% (95%CI: 42.9% - 51.5%, p<0.001) reduction in planned and 35.3% (95%CI: 28.3% - 41.6%; p<0.001) reduction in unplanned weekly admission rates per 1000 people associated with the initiation of the first national lockdown (planned: 2.51 to 1.32; unplanned: 1.36 to 0.88). The initiation of the second national lockdown was also associated with a significant reduction in secondary HCU; planned weekly admission rates per 1000 people reduced by 20.4% (95%CI 14.4%-25.9%; p<0.001) and unplanned reduced by 15.6% (95%CI 7.3%-23.1%; p<0.001). The reductions were sustained throughout these lockdowns; only unplanned admissions in the third national lockdown were not significantly lower than in pre-pandemic rates (Supplementary Table S3). The patterns observed in secondary admissions were consistent across all three main contributing secondary care providers (Supplementary Figure S5).

Both planned and unplanned weekly admissions were on average lower from the beginning of the first national lockdown up until the end of the study period, compared to the pre-pandemic admissions (planned: RR 0.850, 95%CI 0.837 – 0.864, p<0.001; unplanned: RR 0.976, 95%CI 0.957 – 0.996, p=0.016). However, the admissions increased throughout the period (planned: p<0.001; unplanned: p<0.001) and when comparing the final four weeks of the study period with the pre-pandemic period, planned admission rates were not significantly different (RR: 1.105; 95% CI 0.987 – 1.044; p=0.290) and unplanned were higher (RR: 1.104, 95% CI 1.067 – 1.143; p<0.001). The direct comparison between calendar weeks 2-11 in 2021 vs 2020, indicated that planned admissions were lower on average by 11.3% (95% CI: 9.4% - 13.2%; p<0.001) but there was no difference in unplanned admissions (RR: 1.012; 95%CI 0.985 – 1.040; p=0.376). A week-by-week comparison is detailed in Supplementary Table S3.

Secondary HCU by multi-morbidity

Morbidity was associated with an increased rate of planned admissions throughout the study period: One vs No LTCs RR 1.904 (95% CI: 1.717 – 2.111; p<0.001); Multiple vs No LTCs RR 9.584 (95%CI 8.644 – 10.627; p<0.001); Multiple vs One LTC RR 5.033 (95%CI 4.540 – 5.581; p<0.001). This was also the case for unplanned admissions: One vs No LTCs RR 1.188 (95% CI: 1.112 – 1.270; p<0.001);

For peer review only - http://bmjopen.bmj.com/site/about/guidelines.xhtml

Multiple vs No LTCs RR 3.636 (95%CI 3.401 – 3.887; p<0.001); Multiple vs One LTC RR 3.059 (95%CI 2.862 – 3.271; p<0.001) (Figure 3).

Whilst the ratio of weekly unplanned admissions per 1000 people between patients that were multi-morbid vs those without any LTCs was consistent throughout the majority of the pandemic, there was a significant increase during the first lockdown compared with pre-pandemic (RR 1.253, 95%CI 1.068 – 1.469; p=0.006) but no significant change was observed between those with one LTC and those without any LTCs (RR 0.987, 95%CI 0.842 – 1.157; p=0.865, Supplementary Figure S6). The ratio of planned admission rates per 1000 people in morbidity groups increased during the first national lockdown compared to that observed pre-pandemic: multi-morbid vs no LTC increased by a factor of 2.402 (95% CI 2.047 – 2.818; p<0.001) and one LTC vs no LTCs increased by a factor of 1.413 (95% CI 1.205 – 1.658; p<0.001) (Supplementary Table S5; Supplementary Figure S6), however this was not sustained throughout the pandemic. The average of the ratios of admission rates between multi-morbid patients vs patients without LTCs from the start of the first national lockdown until the end of the study period vs pre-pandemic was 1.176 (95% CI: 0.871 – 1.588; p =0.289) for planned admission rates and 1.097 (95%CI: 0.900 – 1.338; p=0.357) for unplanned admission rates.

Secondary care HCU for specific LTC groups

There were noticeable differences for both planned and unplanned admission rates within each LTC group over the study period (Figure 4). Planned admission rates were highest for patients with a renal or urological LTC. Unplanned admission rates were highest in patients with cancer or renal LTCs. Planned and unplanned admission rates were lowest overall for patients without any LTC. For cancer patients, the drop in the number of planned admissions at the initiation of the first national lockdown was sustained throughout the remainder of the study period, with an average reduction of 28.9% (95% CI: 25.1% - 32.5%; p<0.001) compared to pre-pandemic levels and unplanned admission rates decreased by 11.5% (95% CI 2.4% - 19.6%; p=0.014). Planned admission rates for people identified as having an endocrine, musculoskeletal or skin, neurological, psychiatric, or respiratory LTC returned to pre-pandemic levels by the end of the study period. However, planned admission rates for people identified as having cancer, cardiovascular, gastrointestinal, renal or urological and sensory impairment or learning disability remained lower than in the pre-pandemic period. Conversely, planned admission rates for people identified with a substance abuse LTC were higher by the end of the study period compared to the pre-pandemic period (RR: 1.196; 95%CI 1.074 – 1.329; p=0.001; Supplementary Table S11). Unplanned admissions rates were lower only for those that were identified with a renal or urological LTC. Patient groups with a gastrointestinal, musculoskeletal or skin, psychiatric or substance abuse LTC had higher rates of unplanned admissions at the end of the study compared to pre-pandemic levels. The remaining LTC groups had no significant change in unplanned admissions (Supplementary Table S11).

Secondary HCU by deprivation

People from the most deprived areas (IMD of 1 or 2) had the highest rates for both planned (RR (95%CI); 3-4 vs 1-2: 0.753 (0.694 – 0.818; p<0.001); 5-6 vs 1-2: 0.787 (0.724 – 0.854;p<0.001); 7-10 vs 1-2: 0.812 (0.748 – 0.882; p<0.001)) and unplanned admissions (RR (95%CI); 3-4 vs 1-2: 0.686 (0.642 – 0.732; p<0.001); 5-6 vs 1-2: 0.670 (0.628 – 0.715; p<0.001); 7-10 vs 1-2: 0.683 (0.640 – 0.729; p<0.001))

For peer review only - http://bmjopen.bmj.com/site/about/guidelines.xhtml

throughout the study period. For multi-morbid patients, being from a highly deprived area was associated with an increased rate in both planned and unplanned admissions compared to all other deprivation areas (Supplementary Table S3c; Supplementary Figure S7). The ratios of rates between deprivation area groups within multi-morbid patients were not significantly different during the first national lockdown compared to pre-pandemic levels (Supplementary Table S10, Supplementary Figure S8).

DISCUSSION

Principle Findings and Interpretation

We have assessed primary HCU for over 3 million patients across GM and secondary HCU for a sub-group of almost 700,000 patients within Manchester CCG. Major changes in HCU occurred during the COVID-19 pandemic. There was a large reduction in both primary and secondary HCU at the beginning of the first national lockdown. Whilst there was a relatively consistent increase in primary care HCU from the first national lockdown, primary HCU remained was lower at the end of the study compared to pre-pandemic. Overall, both planned and unplanned secondary admissions had recovered to pre-pandemic levels by the end of the study period but this recovery was not observed across all LTC subgroups. Changes in the ratio of HCU between multi-morbid patients and those without LTCs occurred during national lockdowns but were inconsistent across primary HCU measures.

Although some healthcare information measures can be completed remotely (e.g. smoking status, BP and BMI), primary HCU measures that require in-person contact with a healthcare professional (e.g. HbA1c and cholesterol) demonstrated similar patterns in HCU. The initial larger fall in healthcare information recording compared to incident prescribing in primary care may reflect a shift in focus away from secondary prevention during the first wave of the pandemic. It is also possible that coding practices may have changed with the switch from face-to-face to remote consultations and that this switch has also impacted upon opportunistic BP/BMI/smoking status checks. Although these HCU measures have not returned to pre-pandemic levels, they have consistently increased since the first lockdown and this has occurred even though quality outcome framework targets and local enhanced services were largely suspended.

Despite a peak in COVID-19 admissions within the first national lockdown, secondary admissions fell by a larger volume. Reductions in secondary care admissions associated with the first lockdown have been reported across the UK.[11,24,25] Largely, these are reflective of cancellations of elective activity or delaying non-urgent care, to ensure capacity for patients with severe COVID-19 infection and to increase critical care capacity.[7] The observed deficit may not correspond entirely to an unmet need of patients with non-COVID-19 healthcare needs as there is some evidence that changes in behaviour according to sanitisation campaigns, social distancing and government restrictions may have resulted in fewer infections,[26] and injuries.[27] Additionally, emergency department attendances which are related to unplanned admissions (but were not directly assessed in this study) have been observed to have reduced.[14] It is also possible that the increased utilisation of remote management for secondary care patients has contributed to clinically appropriate reductions in admissions.

For peer review only - http://bmjopen.bmj.com/site/about/guidelines.xhtml

There has been no noticeable recovery in HCU for patients with cancer and for a number of other LTCs, recovery to pre-pandemic HCU levels has not occurred. In contrast, HCU of patients identified with substance abuse and/or a psychiatric condition exceeded pre-pandemic levels between the first and second national lockdowns, likely reaffirming the significant impact of the pandemic on mental health and psychiatric services.[28]

Implications for Clinicians and Policy Makers

It is inevitable that the changes in HCU observed in this study will have had an impact on both patients and healthcare providers above and beyond the direct impact of COVID-19. For patients with cancer, services had to adapt to mitigate the increased risk of death from COVID-19.[29] The initial reduction in the number of planned admissions was sustained throughout the study period and is likely to reflect changes in services but may also be due to patients with cancer being reluctant to seek healthcare. Delays in care for patients with cancer are known to impact prognosis,[30] and the pandemic has been found to have contributed to excess deaths in patients with cancer.[24] A proactive approach to encourage patients to attend screening and routine appointments will be needed to minimise the impact of the pandemic on patients with cancer and other emerging health inequalities.[31,32] Understanding the implications of reductions in the selected primary care HCU measures, particularly the decrease in assessing and recording healthcare information will require further long-term studies.

Strengths & Limitations

The strengths of this study include the complete coverage of a large geographical area for the primary care analyses and the inclusion of both primary and secondary HCU data. This is the first study to evaluate HCU across the full spectrum of LTC subpopulations and stratify according to multi-morbidity and deprivation. Data prior to 2020 were not available and consequently comparisons made (pre- vs post-pandemic) are reliant on the data between January and March 2020 being representative of pre-pandemic utilisation. Consequently, the comparison of pre-pandemic HCU to the end of the study period may have been influenced by seasonal variations in HCU. The secondary care analysis was only possible on a subset of the GM population due to delays in data from some GM secondary care providers. A most common provider method was used to assign patients for the secondary care analysis. A limitation of this approach is that patients may travel for secondary care to different hospitals outside of their assigned provider. While this limitation may impact on the rates of utilisation per 1000 people, it is highly unlikely to have caused variation in the rate over time.

The study population represents a largely deprived area placed under strict restrictions during the pandemic. While this information is valuable, the findings may not be generalizable to other settings in the UK or internationally. Although the measures of HCU that have been selected are relevant and reliable, they do not provide a complete picture of either primary or secondary HCU. There is no single effective measure to summarise HCU in primary care as there are many aspects that reflect HCU in this setting.[33] It remains possible that the shift towards increased remote consultations may have resulted in changes to primary care delivery that were not possible to accurately capture using our measures of primary HCU. Additionally, the cause of admission was not available for secondary

For peer review only - http://bmjopen.bmj.com/site/about/guidelines.xhtml

HCU, hence we were unable to determine LTC-specific admissions. Whilst the current scaling and sub-populations do not take into consideration any deaths or new diagnoses that occurred after 1 Jan 2020, a sensitivity analysis accounting for deaths resulted in very small increases to rates (Supplementary Figures S9-S10; Supplementary Table S12) and scaled utilisation remained lower than pre-lockdown levels.

Conclusions

We have assessed the changes in HCU in primary and secondary care associated with the COVID-19 pandemic and UK national lockdowns for patients with LTCs across a large urban region. There was a significant reduction in both primary and secondary HCU associated with the first national lockdown. Subsequent national lockdowns were associated with reductions in secondary care but not in primary care. Whilst some measures of healthcare utilisation had returned to pre-pandemic levels by the end of the study, many had not. Proportionally, secondary care HCU increased in multi-morbid patients compared to those without LTCs during the first and second national lockdowns. Although changes to HCU during the pandemic have been similar overall, different patterns have been seen in specific LTC groups such as people with cancer. Over the course of the pandemic deprivation was associated with higher rates of HCU in multi-morbid patients but no significant differences were observed in the ratio of utilisation between those residing in the most and least deprived areas for the majority of HCU measures during national lockdowns.

ACKNOWLEDGEMENTS

We would like to thank Eric Lowndes and Sumaira Naseem for providing the public representation throughout our research. The authors recognise the Greater Manchester Care Record (a partnership of Greater Manchester Health and Social Care Partnership, Health Innovation Manchester and Graphnet Health, on behalf of Greater Manchester localities) in the provision of data required to undertake this work. Using patient data is vital to improve health and care for everyone. There is huge potential to make better use of information from people's patient records, to understand more about disease, develop new treatments, monitor safety, and plan NHS services. Patient data should be kept safe and secure, to protect everyone's privacy, and it's important that there are safeguards to make sure that it is stored and used responsibly. Everyone should be able to find out about how patient data is used.

CONTRIBUTIONS

SG conceived the study. NP and RW led on the acquisition of data for the study. SG, NP, RW, OT, MS and CSP were involved in the design of the study and interpretation of the data. CSP led the analysis of the data, supported by MS. CSP and SG prepared the manuscript. All authors revised the manuscript and approved the final version.

FUNDING

The time of RW and NP was partially funded by the National Institute for Health Research (NIHR) Greater Manchester Patient Safety Translational Research Centre (PSTRC-2016-003) and the NIHR Applied Research Collaboration Greater Manchester (NIHR200174). The time of NP was partially funded by the NIHR Manchester Biomedical Research Centre (IS-BRC-1215-20007). The time of CSP was fully funded by the NIHR Applied Research Collaboration Greater Manchester (NIHR200174).

For peer review only - http://bmjopen.bmj.com/site/about/guidelines.xhtml

The views expressed are those of the author(s) and not necessarily those of the NIHR or the Department of Health and Social Care.

COMPETING INTERESTS

All authors have completed the ICMJE uniform disclosure form at http://www.icmje.org/disclosure-of-interest/ and declare: no support from any organisation for the submitted work; no financial relationships with any organisations that might have an interest in the submitted work in the previous three years; no other relationships or activities that could appear to have influenced the submitted work.

ETHICS APPROVAL

All data made available to the analysts was de-identified and aggregated and therefore did not require specific ethical approval.

DATA SHARING STATEMENT

Data and code were hosted in a secure environment and are not freely available to share.

REFERENCES

1. Scally G, Jacobson B, Abbasi K. The UK's public health response to covid-19. *The BMJ*. 2020;369(May):1-3. doi:10.1136/bmj.m1932

2. Dong E, Du H, Gardner L. An interactive web-based dashboard to track COVID-19 in real time. *The Lancet Infectious Diseases*. 2020;20(5):533-534. doi:10.1016/S1473-3099(20)30120-1

3. Bou-Karroum L, Khabsa J, Jabbour M, et al. Public health effects of travel-related policies on the COVID-19 pandemic: A mixed-methods systematic review. *Journal of Infection*. 2021;83(4):413-423. doi:10.1016/j.jinf.2021.07.017

4. Lee JK, Bullen C, Ben Amor Y, et al. Institutional and behaviour-change interventions to support COVID-19 public health measures: A review by the Lancet Commission Task Force on public health measures to suppress the pandemic. *International Health*. 2021;13(5):399-409. doi:10.1093/inthealth/ihab022

5. World Health Organization (WHO). *Maintaining Essential Health Services: Operational Guidance for the COVID-19 Context: Interim Guidance*.; 2020.

6. Blanchet K, Alwan A, Antoine C, et al. Protecting essential health services in low-income and middle-income countries and humanitarian settings while responding to the COVID-19 pandemic. *BMJ Global Health*. 2020;5(10):1-9. doi:10.1136/bmjgh-2020-003675

7. Doidge JC, Gould DW, Ferrando-Vivas P, et al. Trends in intensive care for patients with covid-19 in england, wales, and northern ireland. *American Journal of Respiratory and Critical Care Medicine*. 2021;203(5):565-574. doi:10.1164/rccm.202008-3212OC

8. Mutambudzi M, Niedwiedz C, Macdonald EB, et al. Occupation and risk of severe COVID-19: Prospective cohort study of 120 075 UK Biobank participants. *Occupational and Environmental Medicine*. 2021;78(5):307-314. doi:10.1136/oemed-2020-106731

9. Williams R, Jenkins DA, Ashcroft DM, et al. Diagnosis of physical and mental health conditions in primary care during the COVID-19 pandemic: a retrospective cohort study. *The Lancet Public Health*. 2020;5(10):e543-e550. doi:10.1016/S2468-2667(20)30201-2

For peer review only - http://bmjopen.bmj.com/site/about/guidelines.xhtml

10. Mahase E. Covid-19: Urgent cancer referrals fall by 60%, showing “brutal” impact of pandemic. *BMJ (Clinical research ed)*. 2020;369(June):m2386. doi:10.1136/bmj.m2210

11. Mafham MM, Spata E, Goldacre R, et al. COVID-19 pandemic and admission rates for and management of acute coronary syndromes in England. *The Lancet*. 2020;396(10248):381-389. doi:10.1016/S0140-6736(20)31356-8

12. Kwok CS, Gale CP, Kinnaird T, et al. Impact of COVID-19 on percutaneous coronary intervention for ST-elevation myocardial infarction. *Heart*. 2020;106(23):1805-1811. doi:10.1136/heartjnl-2020-317650

13. Nepogodiev D, Omar OM, Glasbey JC, et al. Elective surgery cancellations due to the COVID-19 pandemic: global predictive modelling to inform surgical recovery plans. *British Journal of Surgery*. 2020;107(11):1440-1449. doi:10.1002/bjs.11746

14. Moynihan R, Sanders S, Michaleff ZA, et al. Impact of COVID-19 pandemic on utilisation of healthcare services: A systematic review. *BMJ Open*. 2021;11(3):11-17. doi:10.1136/bmjopen-2020-045343

15. Marmot M, Allen J, Boyce T, Goldblatt P, Morrison J. *Build Back Fairer in Greater Manchester : Health Equity and Dignified Lives*. Institute of Health Equity; 2021.

16. UK Health Security Agency. Coronavirus (COVID-19) in the UK. https://coronavirus.data.gov.uk/details/download

17. Department of Health and Social Care. *COPI Notice*.; 2020.

18. Barnett K, Mercer SW, Norbury M, Watt G, Wyke S, Guthrie B. Epidemiology of multimorbidity and implications for health care, research, and medical education: A cross-sectional study. *The Lancet*. 2012;380(9836):37-43. doi:10.1016/S0140-6736(12)60240-2

19. Ministry of Housing Communities and Local Governments. *The English Indices of Deprivation Things You Need to Know (IoD2019)*.; 2019.

20. Wickham H, Averick M, Bryan J, et al. Welcome to the Tidyverse. *Journal of Open Source Software*. 2019;4(43):1686. doi:10.21105/joss.01686

21. Wickham H, Seidel D. scales: Scale Functions for Visualization. Published online 2020.

22. Wickham H. Reshaping data with the reshape package. *Journal of Statistical Software*. 2007;21(12):1-20. doi:10.18637/jss.v021.i12

23. Wilke CO. cowplot: Streamlined Plot Theme and Plot Annotations for “ggplot2.” Published online 2020.

24. Lai AG, Pasea L, Banerjee A, et al. Estimated impact of the COVID-19 pandemic on cancer services and excess 1-year mortality in people with cancer and multimorbidity: Near real-time data on cancer care, cancer deaths and a population-based cohort study. *BMJ Open*. 2020;10(11):1-9. doi:10.1136/bmjopen-2020-043828

25. Ball S, Banerjee A, Berry C, et al. Monitoring indirect impact of COVID-19 pandemic on services for cardiovascular diseases in the UK. *Heart*. 2020;106(24):1890-1897. doi:10.1136/heartjnl-2020-317870

For peer review only

26. Hussain AZ, Paudyal V, Hadi MA. Impact of the COVID-19 pandemic on the prescribing patterns of first-line antibiotics in english primary care: A longitudinal analysis of national prescribing dataset. *Antibiotics*. 2021;10(5):4-12. doi:10.3390/antibiotics10050591

27. Sephton BM, Mahapatra P, Shenouda M, Ferran N, Deierl K, Sinnett T. The effect of COVID-19 on a Major Trauma Network. An analysis of mechanism of injury pattern, referral load and operative case-mix. *Injury*. 2021;52:395-401.

28. Öngür D, Perlis R, Goff D. Psychiatry and COVID-19. *Journal of the American Medical Association*. 2020;324(12):1149-1150. doi:10.1001/jama.2020.9740

29. Pinato DJ, Scotti L, Gennari A, et al. Determinants of enhanced vulnerability to coronavirus disease 2019 in UK patients with cancer: a European study. *European Journal of Cancer*. 2021;150:190-202.

30. Sud A, Jones ME, Broggio J, et al. Collateral damage: the impact on outcomes from cancer surgery of the COVID-19 pandemic. *Annals of Oncology*. 2020;31(8):1065-1074. doi:10.1016/B978-0-323-60984-5.00062-7

31. Campbell C, Sommerfield T, Clark GRC, et al. COVID-19 and cancer screening in Scotland: A national and coordinated approach to minimising harm. *Preventive Medicine*. 2021;151(October):106606.

32. Kontopantelis E, Mamas MA, Webb RT, et al. Excess deaths from COVID-19 and other causes by region, neighbourhood deprivation level and place of death during the first 30 weeks of the pandemic in England and Wales: A retrospective registry study. *The Lancet Regional Health - Europe*. 2021;7:100144. doi:10.1016/j.lanepe.2021.100144

33. Da Silva RB, Contandriopoulos AP, Pineault R, Tousignant P. A global approach to evaluation of health services utilization: Concepts and measures. *Healthcare Policy*. 2011;6(4):106-117. doi:10.12927/hcpol.2011.22351

FIGURE LEGENDS

Figure 1: Weekly primary care utilisation per 1000 people of the Greater Manchester population between January 2020 and August 2021. The first week covered 30th December 2019 to 5th January 2020, hence utilisation was expected to be considerably lower between this and the following week due to the UK bank holiday and seasonal effects expected for this calendar week.

Figure 2: Rates of primary care measures recorded per 1000 people per week, identified according to number of long-term conditions and deprivation group, between January 2020 and August 2021.

Figure 3: (A:) Weekly rates of planned and unplanned admissions per 1000 people that were identified as having zero, one or multiple LTCs, within the Manchester CCG subpopulation between January 2020 and August 2021. (B:) Government reported COVID-19 admissions in Manchester University NHS Foundation Trust, Pennine Acute Hospitals NHS Foundation Trust and Pennine Care

For peer review only

NHS Foundation Trust (extracted 08-Sept-21), and (C:) Government reported cases in Manchester (extracted 08-Sept- 21). MFT = Manchester University Hospital Foundation Trust, Pennine = Pennine Acute Hospitals NHS Trust & Pennine Care NHS Foundation Trust

Figure 4: Weekly rates of planned and unplanned admissions identified in patients with each of the long-term conditions within the Manchester CCG subpopulation, between January 2020 and August 2021.

For peer review only

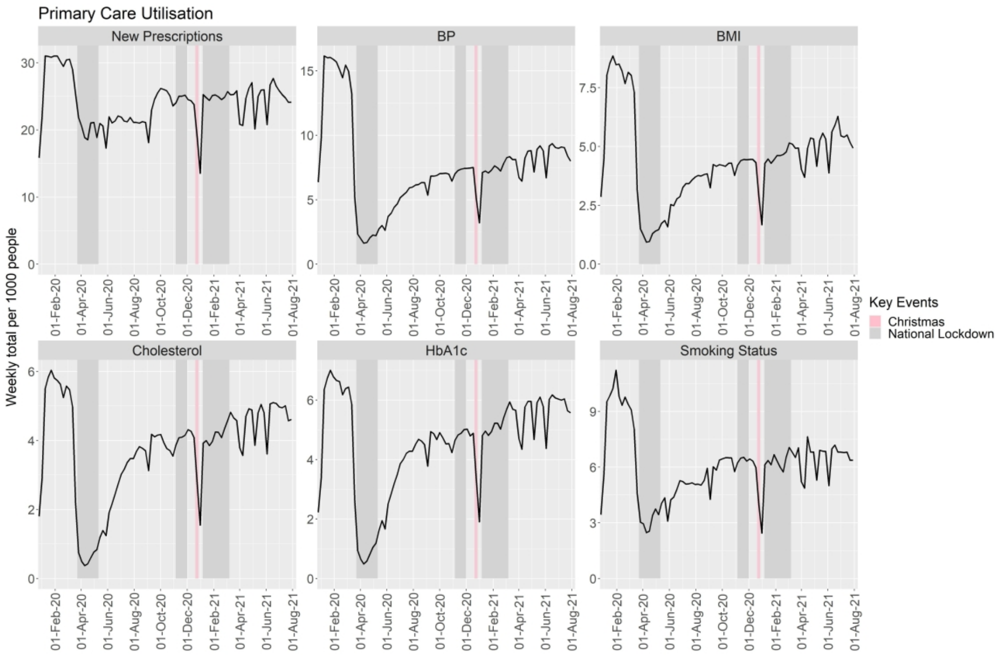

Figure 1: Weekly primary care utilisation per 1000 people of the Greater Manchester population between January 2020 and August 2021. The first week covered 30th December 2019 to 5th January 2020, hence utilisation was expected to be considerably lower between this and the following week due to the UK bank holiday and seasonal effects expected for this calendar week.

508x338mm (300 x 300 DPI)

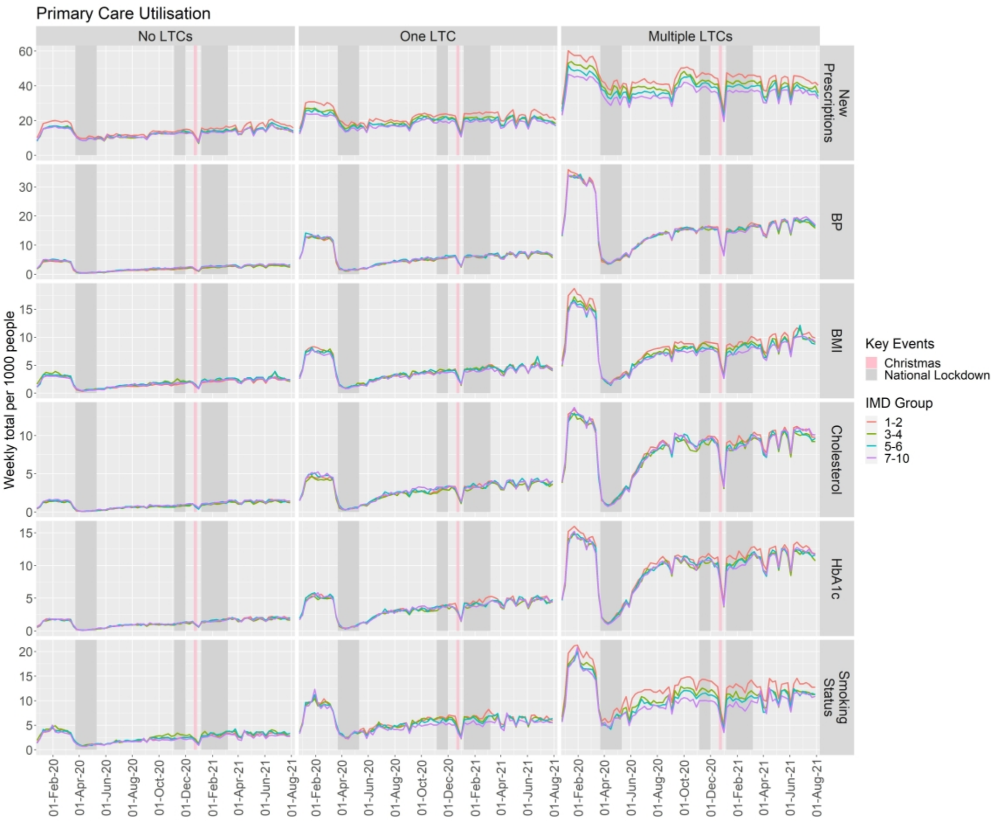

Figure 2: Rates of primary care measures recorded per 1000 people per week, identified according to number of long-term conditions and deprivation group, between January 2020 and August 2021.

508x423mm (300 x 300 DPI)

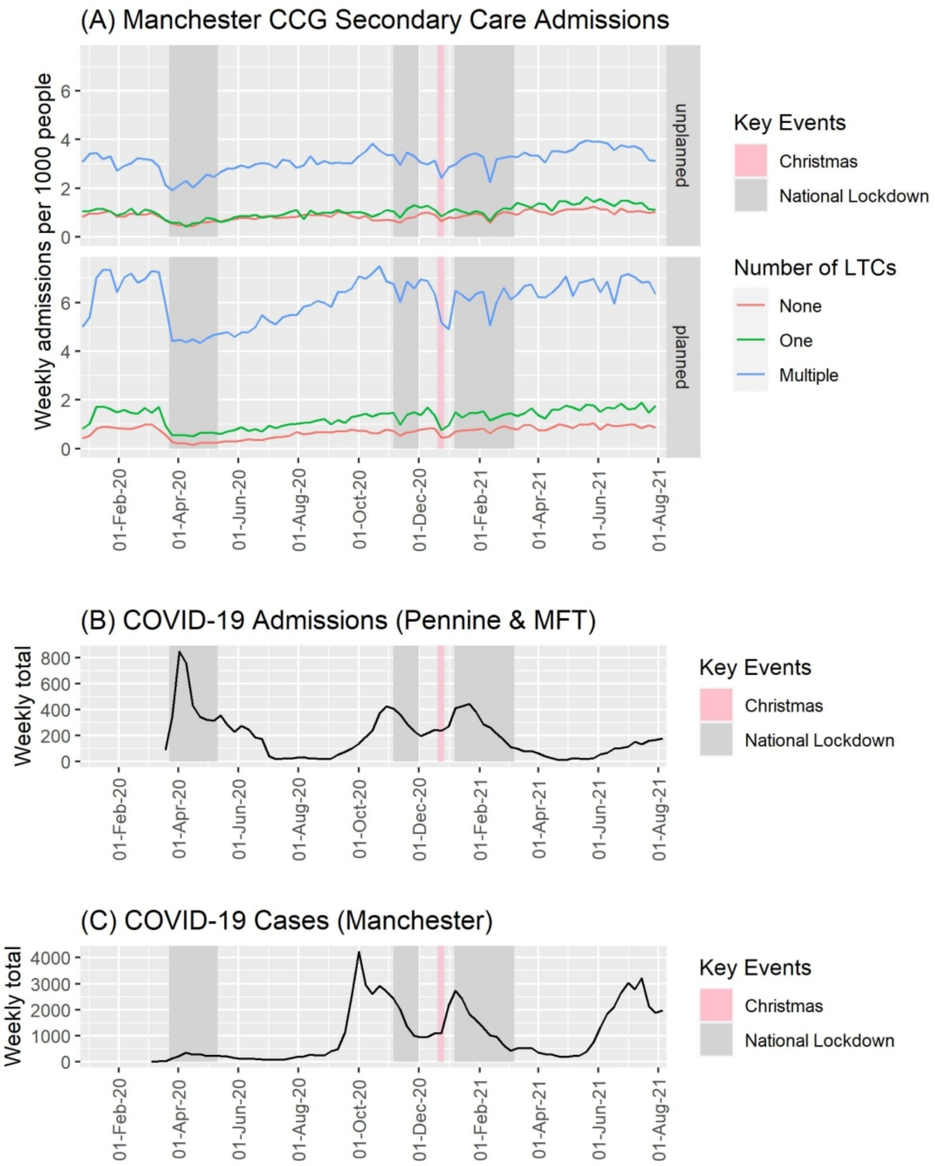

Figure 3: (A:) Weekly rates of planned and unplanned admissions per 1000 people that were identified as having zero, one or multiple LTCs, within the Manchester CCG subpopulation between January 2020 and August 2021. (B:) Government reported COVID-19 admissions in Manchester University NHS Foundation Trust, Pennine Acute Hospitals NHS Foundation Trust and Pennine Care NHS Foundation Trust (extracted 08-Sept-21), and (C:) Government reported cases in Manchester (extracted 08-Sept- 21). MFT = Manchester University Hospital Foundation Trust, Pennine = Pennine Acute Hospitals NHS Trust & Pennine Care NHS Foundation Trust.

169x211mm (300 x 300 DPI)

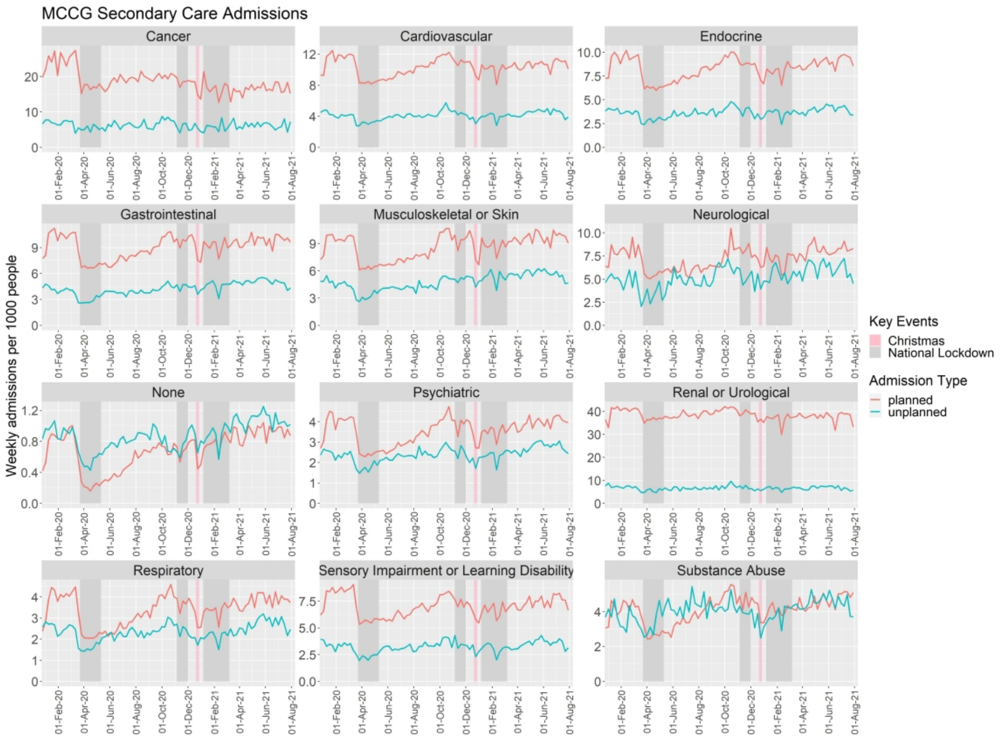

Figure 4: Weekly rates of planned and unplanned admissions identified in patients with each of the long-term conditions within the Manchester CCG subpopulation, between January 2020 and August 2021.

508x381mm (300 x 300 DPI)

SUPPLEMENTARY MATERIALS

COVID-19 Government data extraction

The Government data were extracted on 08-09-2021 from https://api.coronavirus.data.gov.uk/ . For the new cases, we extracted data for Manchester only. The API for Manchester cases by specimen date that was used is:

https://api.coronavirus.data.gov.uk/v2/data?areaType=utla&areaCode=E08000003&metric=newCasesBySpecimenDate&format=csv

For the secondary admissions, we exported the data for the trusts which were the main providers in the Manchester CCG subpopulation. The hospital groupings were not consistent across data sources; Table S2 provides the mapping between the Government listed NHS trusts and the secondary providers covering nearly all admissions experienced by Manchester CCG population in the GMCR.

Supplementary Table S1: Mapping between NHS acute providers according to data source

GMCR acute provider	Government listed NHS Trust	Government data API
University Hospital of South Manchester<br>Central Manchester University Hospitals	Manchester University NHS Foundation Trust	https://api.coronavirus.data.gov.uk/v2/data?areaType=nhsTrust&areaCode=R0A&metric=newAdmissions&format=csv
Pennine Acute Hospitals	Pennine Acute Hospitals NHS Trust	https://api.coronavirus.data.gov.uk/v2/data?areaType=nhsTrust&areaCode=RW6&metric=newAdmissions&format=csv
Pennine Acute Hospitals	Pennine Care NHS Foundation Trust	https://api.coronavirus.data.gov.uk/v2/data?areaType=nhsTrust&areaCode=RT2&metric=newAdmissions&format=csv

For peer review only

Supplementary Table S2: Long-term medical conditions and their groupings

Grouping	Long-term medical condition
**Cardiovascular**	Hypertension<br>Atrial fibrillation<br>Heart failure<br>Peripheral vascular disease<br>Stroke & transient ischaemic attack<br>Coronary heart disease
**Respiratory**	Bronchiectasis<br>Asthma<br>Chronic obstructive pulmonary disease<br>Chronic sinusitis
**Gastrointestinal**	Viral Hepatitis<br>Chronic liver disease<br>Inflammatory bowel disease<br>Diverticular disease of intestine<br>Treated constipation<br>Irritable bowel syndrome<br>Treated dyspepsia
**Neurological**	Multiple sclerosis<br>Parkinson's disease<br>Migraine<br>Epilepsy
**Endocrine**	Thyroid disorders<br>Diabetes
**Psychiatric**	Anorexia or bulimia<br>Schizophrenia (and related non-organic psychosis) or bipolar disorder<br>Dementia<br>Anxiety & other neurotic, stress related & somatoform disorders<br>Depression
**Substance Abuse**	Alcohol problems<br>Other psychoactive substance misuse
**Musculoskeletal/Skin**	Psoriasis or eczema<br>Rheumatoid arthritis, other inflammatory polyarthropathies & systematic connective tissue disorders<br>Painful condition
**Sensory impairment or learning disability**	Learning disability<br>Blindness & low vision<br>Glaucoma<br>Hearing loss
**Renal/Urological**	Chronic kidney disease<br>Prostate disorders

For peer review only

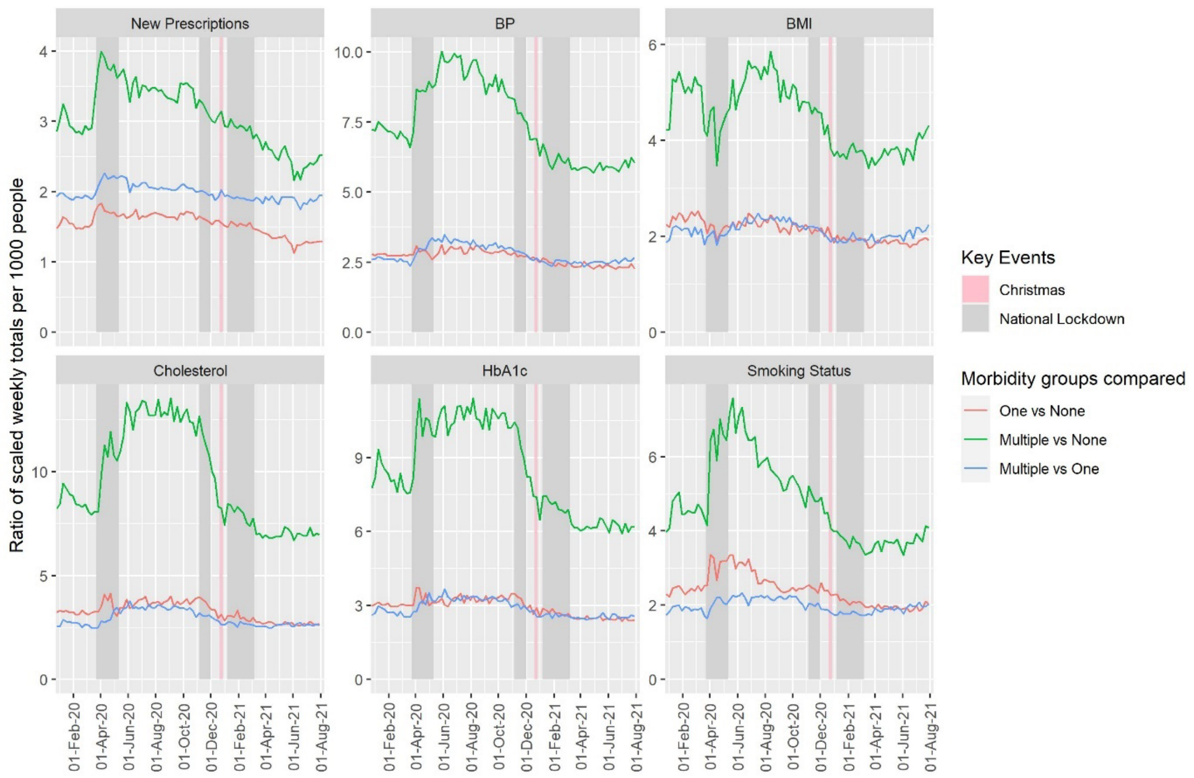

Supplementary Figure S1: Ratio of weekly primary care HCU measures per 1000 people between morbidity groups, between January 2020 and August 2021.

review only

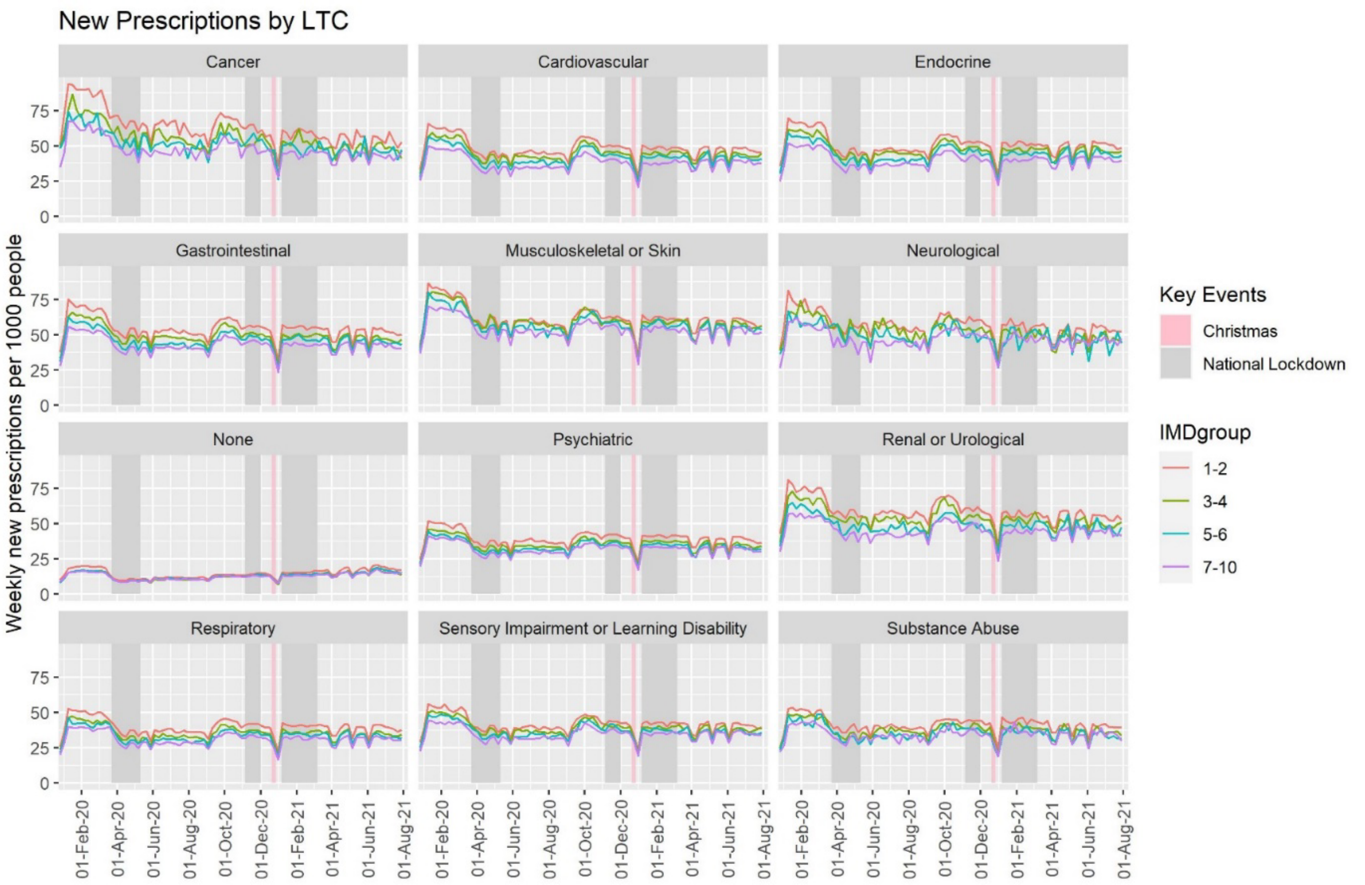

Supplementary Figure S2: Weekly new primary care prescriptions per 1000 people within each long-term condition group and deprivation group, between January 2020 and August 2021.

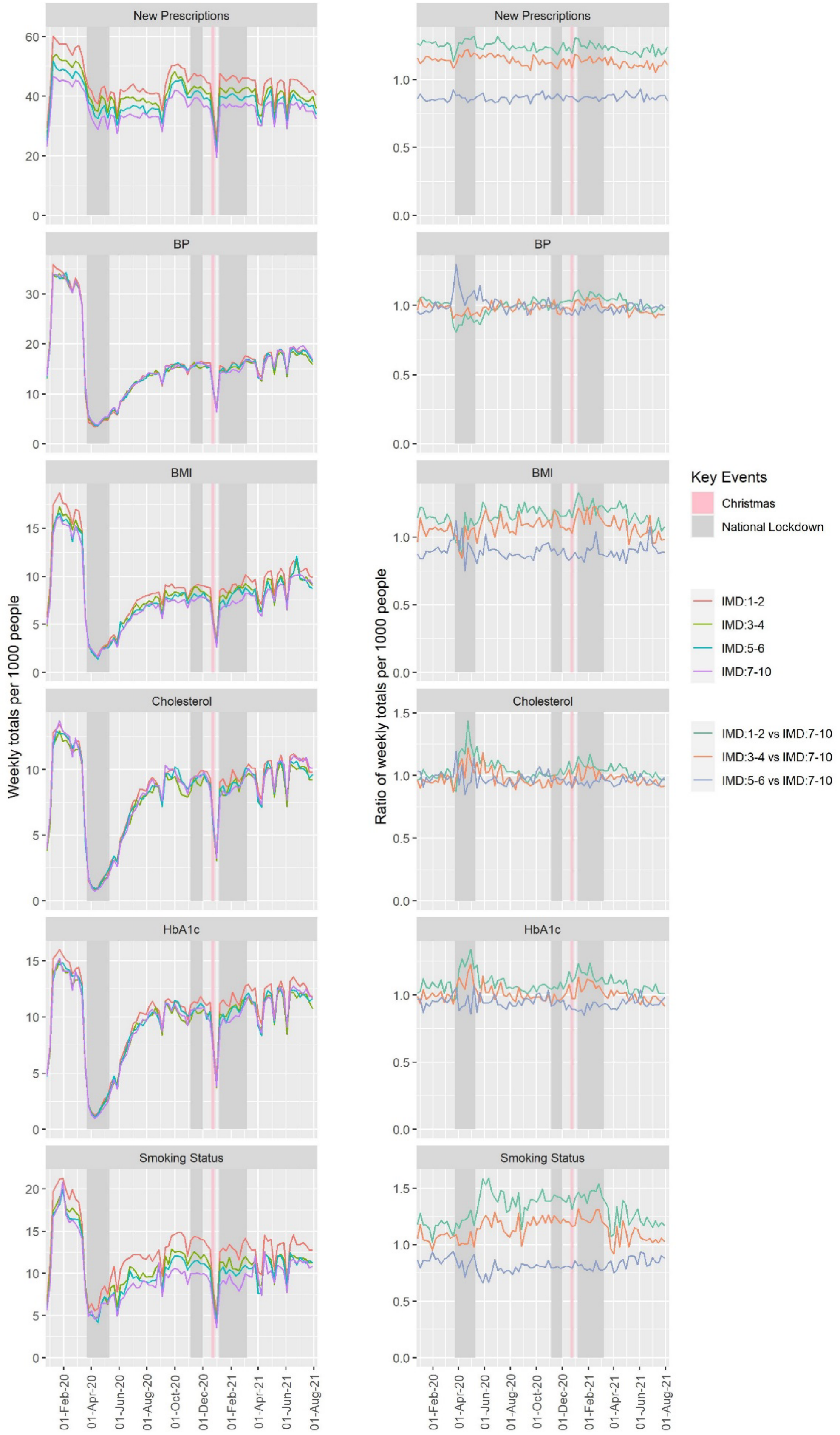

Supplementary Figure S3: Weekly new primary care HCU measures per 1000 people within multi-morbid patients by deprivation group and the ratio of these compared to the least-deprived group (IMD: 7-10), between January 2020 and August 2021.

Supplementary Table S3: Associated effects of the national lockdowns on primary HCU compare to pre-pandemic HCU. NL1 = First national lockdown, NL2 = Second national lockdown, NL3 = Third national lockdown.

	Rate Ratio	95% CI	p-value
	**New Prescriptions**		
NL1 vs pre-pandemic	0.681	0.679 – 0.684	**<0.001**
NL2 vs pre-pandemic	0.835	0.832 – 0.838	**<0.001**
NL3 vs pre-pandemic	0.843	0.841 – 0.846	**<0.001**
	**BP**		
NL1 vs pre-pandemic	0.143	0.142 – 0.144	**<0.001**
NL2 vs pre-pandemic	0.494	0.491 – 0.497	**<0.001**
NL3 vs pre-pandemic	0.510	0.508 – 0.513	**<0.001**
	**BMI**		
NL1 vs pre-pandemic	0.168	0.167 – 0.170	**<0.001**
NL2 vs pre-pandemic	0.559	0.555 – 0.564	**<0.001**
NL3 vs pre-pandemic	0.592	0.588 – 0.596	**<0.001**
	**Cholesterol**		
NL1 vs pre-pandemic	0.128	0.126 – 0.130	**<0.001**
NL2 vs pre-pandemic	0.769	0.762 – 0.776	**<0.001**
NL3 vs pre-pandemic	0.788	0.782 – 0.793	**<0.001**
	**HbA1c**		
NL1 vs pre-pandemic	0.148	0.146 – 0.150	**<0.001**
NL2 vs pre-pandemic	0.785	0.778 – 0.791	**<0.001**
NL3 vs pre-pandemic	0.837	0.832 – 0.842	**<0.001**
	**Smoking Status**		
NL1 vs pre-pandemic	0.346	0.344 – 0.349	**<0.001**
NL2 vs pre-pandemic	0.692	0.687 – 0.696	**<0.001**
NL3 vs pre-pandemic	0.689	0.685 – 0.692	**<0.001**
	**Planned Admissions**		
NL1 vs pre-pandemic	0.544	0.530 – 0.559	**<0.001**
NL2 vs pre-pandemic	0.921	0.897 – 0.946	**<0.001**
NL3 vs pre-pandemic	0.902	0.883 – 0.922	**<0.001**
	**Unplanned Admissions**		
NL1 vs pre-pandemic	0.659	0.638 – 0.681	**<0.001**
NL2 vs pre-pandemic	0.953	0.922 – 0.986	**0.005**
NL3 vs pre-pandemic	0.984	0.958 – 1.010	0.227

For peer review only

Supplementary Table S4: Percentage decrease (95%CI) in HCU in 2021 compared with the same calendar week in 2020.

Calendar Week	2	3	4	5	6	7	8	9	10	11	Average	p-value
**Primary HCU**												
New prescriptions	20.0 (19.3 – 20.8)	21.2 (20.5 – 21.9)	18.4 (17.7 – 19.2)	18.7 (18.0 – 19.5)	19.6 (18.9 – 20.3)	18.7 (17.9 – 19.5)	15.5 (14.8 – 16.3)	15.5 (14.8 – 16.3)	17.2 (16.5 – 18.0)	12.8 (12.0 – 13.6)	17.8 (17.6 – 18.1)	<0.001
Blood Pressure	55.2 (54.5 – 55.9)	55.8 (55.1 – 56.5)	54.4 (53.7 – 55.1)	52.0 (51.3 – 52.7)	52.4 (51.6 – 53.1)	52.1 (51.4 – 52.9)	45.9 (45.0 – 46.7)	46.4 (45.7 – 47.2)	44.3 (43.4 – 45.1)	38.7 (37.8 – 39.7)	50.0 (49.8 – 50.2)	<0.001
BMI	44.4 (43.3 – 45.5)	49.7 (48.7 – 50.8)	49.7 (48.7 – 50.8)	45.3 (44.3 – 46.5)	45.7 (44.6 – 46.8)	43.2 (42.0 – 44.3)	37.8 (36.6 – 39.1)	36.7 (35.6 – 38.0)	36.3 (35.1 – 37.6)	32.6 (31.3 – 34.0)	42.5 (42.1 – 42.8)	<0.001
Cholesterol	27.5 (25.8 – 29.1)	34.0 (32.4 – 35.4)	34.2 (32.7 – 35.6)	26.9 (25.3 – 28.5)	26.2 (24.5 – 27.7)	27.4 (25.8 – 29.1)	17.1 (15.3 – 19.0)	18.0 (16.2 – 19.8)	11.9 (10.0 – 13.8)	6.3 (4.2 – 8.4)	18.4 (17.8 – 18.9)	<0.001
HbA1c	22.0 (20.4 – 23.6)	28.6 (27.2 – 30.1)	29.4 (27.9 – 30.8)	23.0 (21.5 – 24.6)	21.7 (20.1 – 23.2)	24.1 (22.6 – 25.7)	12.4 (10.6 – 14.2)	10.7 (9.0 – 12.5)	7.7 (5.9 – 9.5)	2.7 (0.8 – 4.7)	18.6 (18.1 – 19.1)	<0.001
Smoking Status	33.3 (32.1 – 34.5)	37.8 (36.7 – 38.9)	34.7 (33.6 – 35.9)	43.4 (42.6 – 44.5)	38.9 (37.8 – 40.0)	38.4 (37.3 – 39.6)	32.9 (31.8 – 34.2)	24.7 (23.5 – 26.1)	25.1 (23.9 – 26.6)	18.8 (17.4 – 20.4)	33.3 (33.0 – 33.7)	<0.001
**Secondary HCU**												
Planned admissions	11.6 (5.3 – 17.5)	16.8 (10.9 – 22.3)	12.5 (6.3 – 18.2)	-0.4 (-7.6 – 6.3)	27.1 (21.6 – 32.3)	12.5 (6.2 – 18.4)	1.7 (-5.2 – 8.2)	12.5 (6.3 – 18.3)	14.8 (8.9 – 20.4)	2.6 (-4.1 – 8.9)	11.3 (9.4 – 13.2)	<0.001
Unplanned admissions	8.4 (0.2 – 15.9)	3.3 (-5.2 – 11.2)	11.2 (-7.4 – 9.1)	-14.2 (-24.9 – -4.4)	26.9 (19.4 – 33.8)	2.0 (-6.8 – 10.1)	-7.7 (-17.2 – 1.1)	-5.7 (-15.1 – 2.9)	-3.1 (-12.2 – 5.2)	-25.7 (-37.0 – -15.5)	-1.2 (-4.0 – 1.5)	0.376

For peer review only

Supplementary Table S5: Associated effects of morbidity on the rates of weekly totals of primary and secondary HCU (per 1000 people), throughout the study period. LTC = Long term condition.

	Rate Ratio	95% CI	p-value
	**New Prescriptions**		
Multiple LTCs vs No LTCs	3.040	2.881 – 3.208	<0.001
One LTC vs No LTCs	1.534	1.454 – 1.619	<0.001
	**BP**		
Multiple LTCs vs No LTCs	7.347	6.197 – 8.711	<0.001
One LTC vs No LTCs	2.659	2.243 – 3.153	<0.001
	**BMI**		
Multiple LTCs vs No LTCs	4.429	3.801 – 5.162	<0.001
One LTC vs No LTCs	2.106	1.807 – 2.454	<0.001
	**Cholesterol**		
Multiple LTCs vs No LTCs	9.360	7.654 – 11.446	<0.001
One LTC vs No LTCs	3.226	2.638 – 3.945	<0.001
	**HbA1c**		
Multiple LTCs vs No LTCs	8.291	6.847 – 10.038	<0.001
One LTC vs No LTCs	2.918	2.410 – 3.533	<0.001
	**Smoking Status**		
Multiple LTCs vs No LTCs	4.674	4.192 – 5.211	<0.001
One LTC vs No LTCs	2.372	2.127 – 2.645	<0.001
	**Planned Admissions**		
Multiple LTCs vs No LTCs	9.584	8.644 – 10.627	<0.001
One LTC vs No LTCs	1.904	1.717 – 2.111	<0.001
	**Unplanned Admissions**		
Multiple LTCs vs No LTCs	3.636	3.401 – 3.887	<0.001
One LTC vs No LTCs	1.188	1.112 – 1.270	<0.001

For peer review only

Supplementary Table S6: Estimated rate ratios (RRs) from log-linear regression models for each HCU measure observed pre-pandemic and in the national lockdowns, adjusted for the number of long term conditions (LTCs). NL1 = First national lockdown, NL2 = Second national lockdown, NL3 = Third national lockdown.

	Primary Care											
	New Prescriptions			BP			BMI			Cholesterol		
	RR	95% CI	p-value	RR	95% CI	p-value	RR	95% CI	p-value	RR	95% CI	p-value
NL1 vs pre-pandemic	0.580	0.543 - 0.619	**<0.001**	0.126	0.112 - 0.141	**<0.001**	0.190	0.165 - 0.218	**<0.001**	0.103	0.084 - 0.127	**<0.001**
NL2 vs pre-pandemic	0.800	0.742 - 0.862	**<0.001**	0.469	0.409 - 0.537	**<0.001**	0.615	0.523 - 0.722	**<0.001**	0.624	0.490 - 0.796	**<0.001**
NL3 vs pre-pandemic	0.855	0.804 - 0.909	**<0.001**	0.581	0.520 - 0.648	**<0.001**	0.752	0.660 - 0.856	**<0.001**	0.859	0.705 - 1.046	**0.128**
Multiple LTCs vs No LTCs	2.950	2.779 - 3.131	**<0.001**	7.155	6.429 - 7.963	**<0.001**	5.091	4.483 - 5.781	**<0.001**	8.576	7.077 - 10.392	**<0.001**
One LTC vs No LTCs	1.531	1.442 - 1.625	**<0.001**	2.753	2.474 - 3.064	**<0.001**	2.400	2.114 - 2.726	**<0.001**	3.222	2.659 - 3.904	**<0.001**
NL1 vs pre-pandemic : Multiple LTCs vs No LTCs	1.281	1.169 - 1.404	**<0.001**	1.187	1.007 - 1.400	**0.042**	0.847	0.696 - 1.030	0.095	1.220	0.907 - 1.640	0.186
NL2 vs pre-pandemic : Multiple LTCs vs No LTCs	1.073	0.964 - 1.193	0.194	1.091	0.901 - 1.321	0.369	0.911	0.726 - 1.144	0.418	1.318	0.934 - 1.858	0.114
NL3 vs pre-pandemic : Multiple LTCs vs No LTCs	0.984	0.903 - 1.073	0.713	0.859	0.736 - 1.004	0.056	0.736	0.613 - 0.885	**0.001**	0.920	0.697 - 1.216	0.555
NL1 vs pre-pandemic : One LTC vs No LTCs	1.126	1.027 - 1.234	**0.012**	1.025	0.870 - 1.209	0.763	0.889	0.731 - 1.081	0.236	1.102	0.820 - 1.482	0.515
NL2 vs pre-pandemic : One LTC vs No LTCs	1.043	0.937 - 1.160	0.439	0.996	0.823 - 1.206	0.968	0.888	0.708 - 1.115	0.304	1.139	0.808 - 1.606	0.454
NL3 vs pre-pandemic : One LTC vs No LTCs	0.994	0.912 - 1.084	0.895	0.896	0.767 - 1.046	0.162	0.811	0.674 - 0.975	**0.026**	0.921	0.697 - 1.216	0.557
	Primary Care (ctd)						Secondary Care					
	HbA1c			Smoking Status			Planned Admissions			Unplanned Admissions		
	RR	95% CI	p-value	RR	95% CI	p-value	RR	95% CI	p-value	RR	95% CI	p-value

For peer review only

NL1 vs pre-pandemic	0.120	0.096 - 0.148	**<0.001**	0.272	0.238 - 0.310	**<0.001**	0.272	0.243 - 0.305	**<0.001**	0.582	0.511 - 0.663	**<0.001**
NL2 vs pre-pandemic	0.716	0.557 - 0.920	**0.010**	0.667	0.573 - 0.777	**<0.001**	0.820	0.719 - 0.935	**0.003**	0.803	0.690 - 0.934	**0.005**
NL3 vs pre-pandemic	0.967	0.789 - 1.185	0.745	0.816	0.721 - 0.923	**<0.001**	0.935	0.841 - 1.040	0.214	0.933	0.826 - 1.055	0.267
Multiple LTCs vs No LTCs	8.223	6.744 - 10.026	**<0.001**	4.595	4.074 - 5.183	**<0.001**	8.298	7.481 - 9.205	**<0.001**	3.359	2.981 - 3.786	**<0.001**
One LTC vs No LTCs	3.022	2.478 - 3.684	**<0.001**	2.404	2.131 - 2.712	**<0.001**	1.834	1.653 - 2.034	**<0.001**	1.111	0.986 - 1.252	0.083
NL1 vs pre-pandemic : Multiple LTCs vs No LTCs	1.221	0.900 - 1.658	0.197	1.356	1.126 - 1.632	**0.002**	2.402	2.047 - 2.818	**<0.001**	1.253	1.042 - 1.506	**0.017**
NL2 vs pre-pandemic : Multiple LTCs vs No LTCs	1.145	0.803 - 1.633	0.449	1.074	0.866 - 1.332	0.511	1.174	0.975 - 1.413	0.090	1.283	1.036 - 1.588	**0.023**
NL3 vs pre-pandemic : Multiple LTCs vs No LTCs	0.852	0.639 - 1.136	0.271	0.803	0.675 - 0.956	**0.014**	0.957	0.824 - 1.112	0.563	1.073	0.902 - 1.276	0.421
NL1 vs pre-pandemic : One LTC vs No LTCs	1.082	0.797 - 1.468	0.610	1.279	1.062 - 1.540	**0.010**	1.413	1.205 - 1.658	**<0.001**	0.987	0.821 - 1.186	0.885
NL2 vs pre-pandemic : One LTC vs No LTCs	1.058	0.742 - 1.508	0.754	1.028	0.829 - 1.275	0.799	1.063	0.883 - 1.280	0.513	1.295	1.046 - 1.604	**0.018**
NL3 vs pre-pandemic : One LTC vs No LTCs	0.894	0.671 - 1.192	0.442	0.87	0.730 - 1.036	0.116	0.971	0.835 - 1.128	0.695	1.075	0.904 - 1.279	0.407

For peer review only

Supplementary Table S7: Associated effects of deprivation groups on primary and secondary HCU, throughout the study.

	Primary Care											
Deprivation group compared to group 1-2	**New Prescriptions**			**BP**			**BMI**			**Cholesterol**		
	RR	95% CI	p-value	RR	95% CI	p-value	RR	95% CI	p-value	RR	95% CI	p-value
3-4	0.915	0.874 – 0.959	**<0.001**	1.018	0.869 – 1.193	0.821	1.007	0.865 – 1.172	0.928	0.964	0.797 – 1.166	0.702
5-6	0.920	0.878 – 0.964	**<0.001**	1.117	0.954 – 1.309	0.169	1.025	0.881 – 1.192	0.751	1.076	0.889 – 1.302	0.450
7-10	0.875	0.835 – 0.917	**<0.001**	1.134	0.968 – 1.328	0.120	0.988	0.849 – 1.150	0.879	1.091	0.902 – 1.320	0.369
	**Primary Care (ctd)**						**Secondary Care**					
	**HbA1c**			**Smoking Status**			**Planned Admissions**			**Unplanned Admissions**		
	RR	95% CI	p-value	RR	95% CI	p-value	RR	95% CI	p-value	RR	95% CI	p-value
3-4	0.950	0.794 – 1.137	0.574	0.947	0.858 – 1.047	0.287	0.753	0.694 – 0.818	<0.001	0.686	0.642 – 0.732	<0.001
5-6	1.038	0.868 – 1.242	0.682	0.945	0.856 – 1.045	0.269	0.787	0.724 – 0.854	<0.001	0.67	0.628 – 0.715	<0.001
7-10	1.031	0.862 – 1.234	0.737	0.885	0.801 – 0.977	0.016	0.812	0.748 – 0.882	<0.001	0.683	0.640 – 0.729	<0.001

For peer review only

Supplementary Table S8: Estimated rate ratios (RRs) from log-linear regression models for each HCU observed pre-pandemic and in the national lockdowns, adjusted for the deprivation group. NL1 = First national lockdown, NL2 = Second national lockdown, NL3 = Third national lockdown, IMD = index of multiple deprivation.

	**Primary Care**											
	**New Prescriptions**			**BP**			**BMI**			**Cholesterol**		
	RR	95% CI	p-value	RR	95% CI	p-value	RR	95% CI	p-value	RR	95% CI	p-value
NL1 vs pre-pandemic	0.668	0.629 – 0.711	**<0.001**	0.133	0.118 – 0.149	**<0.001**	0.161	0.139 – 0.186	**<0.001**	0.125	0.102 – 0.154	**<0.001**
NL2 vs pre-pandemic	0.819	0.763 – 0.879	**<0.001**	0.491	0.430 – 0.560	**<0.001**	0.555	0.469 – 0.656	**<0.001**	0.782	0.615 – 0.994	**0.045**
NL3 vs pre-pandemic	0.837	0.790 – 0.886	**<0.001**	0.520	0.467 – 0.579	**<0.001**	0.599	0.523 – 0.686	**<0.001**	0.824	0.678 – 1.001	0.051
IMD: 3-4 vs IMD: 1-2	0.895	0.846 – 0.947	**<0.001**	1.011	0.910 – 1.123	0.837	0.994	0.871 – 1.135	0.933	0.977	0.808 – 1.181	0.810
IMD: 5-6 vs IMD: 1-2	0.891	0.843 – 0.943	**<0.001**	1.086	0.978 – 1.206	0.122	0.998	0.874 – 1.139	0.971	1.087	0.899 – 1.314	0.389
IMD: 7-10 vs IMD: 1-2	0.846	0.800 – 0.895	**<0.001**	1.099	0.989 – 1.220	0.079	0.977	0.856 – 1.116	0.732	1.128	0.933 – 1.364	0.213
NL1 vs pre-pandemic : IMD: 3-4 vs IMD: 1-2	1.043	0.956 – 1.137	0.342	1.076	0.916 – 1.265	0.372	1.056	0.861 – 1.296	0.600	0.976	0.729 – 1.308	0.871
NL2 vs pre-pandemic : IMD: 3-4 vs IMD: 1-2	1.033	0.935 – 1.143	0.522	1.006	0.834 – 1.214	0.947	1.070	0.845 – 1.357	0.574	0.984	0.701 – 1.382	0.926
NL3 vs pre-pandemic : IMD: 3-4 vs IMD: 1-2	1.021	0.941 – 1.108	0.617	0.981	0.843 – 1.142	0.806	1.021	0.843 – 1.237	0.831	0.978	0.743 – 1.287	0.872
NL1 vs pre-pandemic : IMD: 5-6 vs IMD: 1-2	1.043	0.956 – 1.137	0.342	1.124	0.956 – 1.320	0.158	1.064	0.867 – 1.305	0.553	1.037	0.774 – 1.389	0.810
NL2 vs pre-pandemic : IMD: 5-6 vs IMD: 1-2	1.048	0.948 – 1.159	0.360	1.029	0.853 – 1.241	0.765	1.022	0.806 – 1.295	0.857	1.000	0.712 – 1.404	0.999
NL3 vs pre-pandemic : IMD: 5-6 vs IMD: 1-2	1.025	0.944 – 1.112	0.559	1.002	0.861 – 1.166	0.980	1.025	0.846 – 1.242	0.803	0.960	0.729 – 1.264	0.773
NL1 vs pre-pandemic : IMD: 7-10 vs IMD: 1-2	1.034	0.949 – 1.128	0.444	1.159	0.986 – 1.363	0.073	1.088	0.887 – 1.334	0.419	0.889	0.663 – 1.191	0.430
NL2 vs pre-pandemic : IMD: 7-10 vs IMD: 1-2	1.048	0.948 – 1.159	0.362	1.037	0.860 – 1.251	0.701	1.018	0.803 – 1.290	0.885	1.006	0.716 – 1.413	0.973
NL3 vs pre-pandemic : IMD: 7-10 vs IMD: 1-2	1.019	0.939 – 1.106	0.648	0.969	0.833 – 1.128	0.686	0.971	0.801 – 1.177	0.764	0.923	0.701 – 1.216	0.570

For peer review only

	Primary Care (ctd)						Secondary Care					
	HbA1c			Smoking Status			Planned Admissions			Unplanned Admissions		
	RR	95% CI	p-value	RR	95% CI	p-value	RR	95% CI	p-value	RR	95% CI	p-value
NL1 vs pre-pandemic	0.142	0.115 – 0.175	**<0.001**	0.352	0.309 – 0.401	**<0.001**	**0.566**	**0.516 - 0.621**	**<0.001**	**0.642**	**0.558 - 0.737**	**<0.001**
NL2 vs pre-pandemic	0.794	0.622 – 1.013	0.064	0.734	0.632 – 0.853	**<0.001**	0.916	0.823 - 1.020	0.110	0.944	0.803 - 1.109	0.479
NL3 vs pre-pandemic	0.871	0.714 – 1.061	0.171	0.735	0.651 – 0.831	**<0.001**	**0.894**	**0.820 - 0.976**	**0.012**	0.945	0.829 - 1.077	0.393
IMD: 3-4 vs IMD: 1-2	0.947	0.781 – 1.149	0.582	0.977	0.868 – 1.101	0.707	**0.761**	**0.699 - 0.828**	**<0.001**	0.654	0.576 - 0.743	**<0.001**
IMD: 5-6 vs IMD: 1-2	1.038	0.856 – 1.259	0.705	0.978	0.868 – 1.101	0.713	**0.799**	**0.734 - 0.870**	**<0.001**	**0.598**	**0.527 - 0.680**	**<0.001**
IMD: 7-10 vs IMD: 1-2	1.041	0.858 – 1.263	0.681	0.972	0.863 – 1.094	0.636	**0.848**	**0.779 - 0.923**	**<0.001**	**0.643**	**0.566 - 0.731**	**<0.001**
NL1 vs pre-pandemic : IMD: 3-4 vs IMD: 1-2	1.000	0.743 – 1.347	0.999	0.958	0.798 – 1.150	0.646	0.988	0.867 - 1.126	0.858	1.097	0.902 - 1.335	0.351
NL2 vs pre-pandemic : IMD: 3-4 vs IMD: 1-2	0.991	0.702 – 1.401	0.961	0.997	0.806 – 1.233	0.977	1.001	0.860 - 1.165	0.990	1.017	0.810 - 1.277	0.883
NL3 vs pre-pandemic : IMD: 3-4 vs IMD: 1-2	0.991	0.749 – 1.311	0.949	0.97	0.816 – 1.152	0.725	0.997	0.881 - 1.127	0.959	1.061	0.882 - 1.276	0.525
NL1 vs pre-pandemic : IMD: 5-6 vs IMD: 1-2	1.020	0.758 – 1.374	0.895	0.974	0.811 – 1.170	0.779	0.765	0.671 - 0.872	**<0.001**	1.098	0.902 - 1.336	0.348
NL2 vs pre-pandemic : IMD: 5-6 vs IMD: 1-2	1.009	0.714 – 1.426	0.958	0.933	0.754 – 1.153	0.519	1.043	0.896 - 1.214	0.588	0.981	0.781 - 1.233	0.871
NL3 vs pre-pandemic : IMD: 5-6 vs IMD: 1-2	0.960	0.726 – 1.270	0.776	0.948	0.798 – 1.126	0.541	1.077	0.952 - 1.218	0.235	1.247	1.037 - 1.500	**0.020**
NL1 vs pre-pandemic : IMD: 7-10 vs IMD: 1-2	0.929	0.690 – 1.251	0.629	0.984	0.819 – 1.181	0.861	0.695	0.609 - 0.792	**<0.001**	0.898	0.738 - 1.093	0.280
NL2 vs pre-pandemic : IMD: 7-10 vs IMD: 1-2	1.019	0.721 – 1.440	0.915	0.852	0.689 – 1.054	0.139	1.032	0.887 - 1.202	0.678	1.173	0.934 - 1.473	0.169
NL3 vs pre-pandemic : IMD: 7-10 vs IMD: 1-2	0.927	0.700 – 1.226	0.595	0.836	0.704 – 0.994	**0.042**	1.068	0.944 - 1.208	0.291	1.164	0.968 - 1.401	0.105

For peer review only

Supplementary Table S9: Comparison of HCU between deprivation groups and the highly deprived population throughout the pandemic for multi-morbid patients.

	Deprivation 3-4 vs 1-2		Deprivation 5-6 vs 1-2		Deprivation 7-10 vs 1-2	
	RR (95% CI)	p-value	RR (95% CI)	p-value	RR (95% CI)	p-value
**Primary HCU**						
New prescriptions	0.917 (0.878 – 0.957)	**<0.001**	0.867 (0.830 – 0.905)	**<0.001**	0.807 (0.773 – 0.843)	**<0.001**
BP	0.983 (0.844 – 1.145)	0.827	0.997 (0.856 – 1.161)	0.969	1.001 (0.860 – 1.166)	0.989
BMI	0.927 (0.796 – 1.081)	0.334	0.893 (0.766 – 1.041)	0.147	0.865 (0.742 – 1.008)	0.063
Cholesterol	0.939 (0.779 – 1.133)	0.510	0.969 (0.803 – 1.168)	0.738	0.958 (0.794 – 1.155)	0.652
HbA1c	0.927 (0.779 – 1.104)	0.396	0.942 (0.791 – 1.122)	0.504	0.919 (0.772 – 1.094)	0.342
Smoking status	0.863 (0.785 – 0.949)	**0.003**	0.820 (0.745 – 0.901)	**<0.001**	0.766 (0.697 – 0.843)	**<0.001**
**Secondary HCU**						
Planned admissions	0.873 (0.773 – 0.986)	**0.029**	0.741 (0.652 – 0.842)	**<0.001**	0.705 (0.619 – 0.802)	**<0.001**
Unplanned admissions	0.826 (0.696 – 0.980)	**0.029**	0.669 (0.557 – 0.802)	**<0.001**	0.645 (0.536 – 0.774)	**<0.001**

For peer review only

Supplementary Table S10: Associated interaction between the first national lockdown and deprivation group on primary HCU compared to pre-pandemic rate ratios between deprivation groups, for multi-morbid patients.

	Deprivation 3-4 vs 1-2		Deprivation 5-6 vs 1-2		Deprivation 7-10 vs 1-2	
	RR (95% CI)	p-value	RR (95% CI)	p-value	RR (95% CI)	p-value
**Primary HCU**						
New prescriptions	1.022 (0.913 – 1.145)	0.697	1.012 (0.904 – 1.133)	0.831	0.992 (0.886 – 1.111)	0.888
BP	1.097 (0.886 – 1.358)	0.390	1.127 (0.910 – 1.396)	0.267	1.166 (0.942 – 1.444)	0.156
BMI	1.018 (0.760 – 1.362)	0.905	1.025 (0.766 – 1.372)	0.865	1.102 (0.823 – 1.475)	0.508
Cholesterol	0.958 (0.644 – 1.424)	0.829	1.004 (0.675 – 1.492)	0.986	0.853 (0.574 – 1.269)	0.428
HbA1c	0.988 (0.665 – 1.467)	0.950	0.996 (0.670 – 1.479)	0.983	0.890 (0.599 – 1.323)	0.561
Smoking status	0.939 (0.726 – 1.215)	0.628	0.945 (0.731 – 1.223)	0.665	0.957 (0.739 – 1.238)	0.733
**Secondary HCU**						
Planned admissions	1.011 (0.576 – 1.770)	0.970	0.748 (0.392 – 1.404)	0.371	0.731 (0.387 – 1.359)	0.326
Unplanned admissions	1.153 (0.513 – 2.592)	0.729	1.202 (0.490 – 2.919)	0.685	0.889 (0.346 – 2.217)	0.803

For peer review only

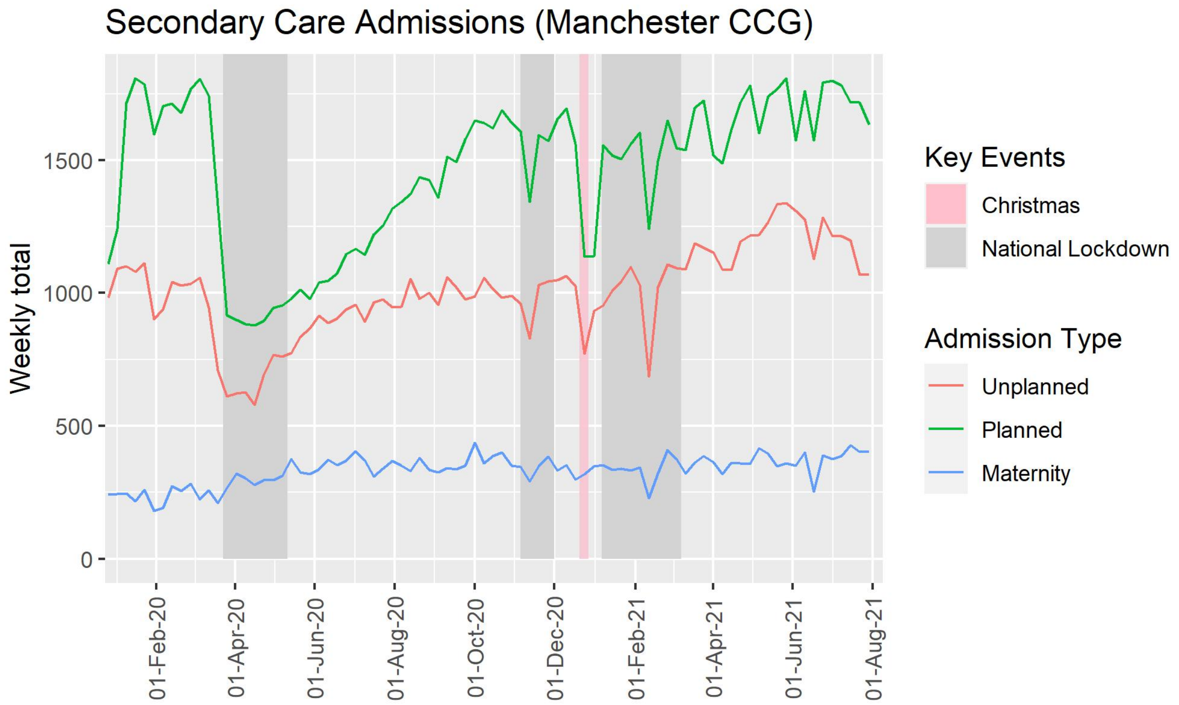

Supplementary Figure S4: Secondary healthcare utilisation across Manchester CCG, from January 2020 until August 2021.

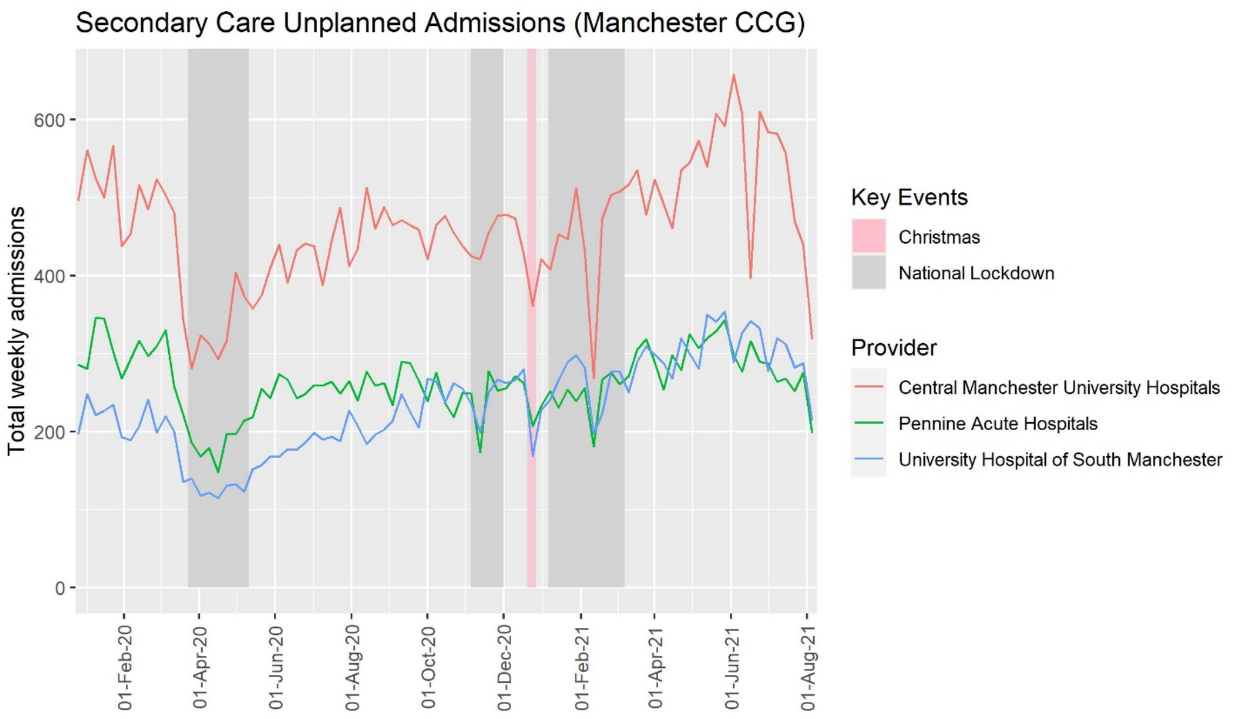

Supplementary Figure S5: Total weekly unplanned admissions of MCCG population at the three main providers (local to 96.6% of people) between January 2020 and August 2021.

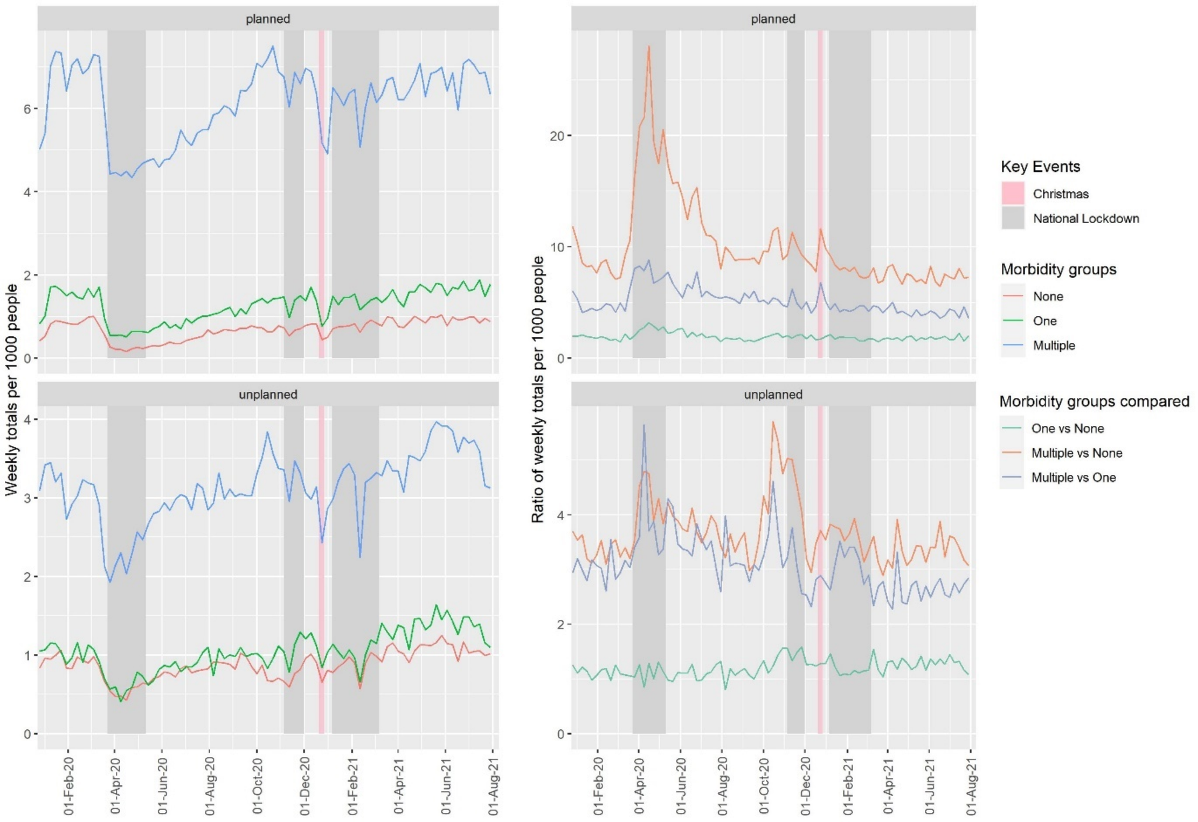

Supplementary Figure S6: Weekly planned and unplanned admissions per 1000 people within morbidity group in the MCCG population and the ratio between these scaled rates, between January 2020 and August 2021.

Supplementary Table S11: Estimates of the ratios of planned and unplanned admission rates for each LTC group in the final four weeks of the study compared to pre-pandemic rates.

	Planned			Unplanned		
LTC	Rate Ratio	95% CI	p-value	Rate Ratio	95% CI	p-value
Cancer	0.653	0.586 – 0.726	**<0.001**	0.926	0.773 – 1.105	0.401
Cardiovascular	0.938	0.901 – 0.977	**0.002**	0.978	0.915 – 1.044	0.502
Endocrine	0.997	0.951 – 1.044	0.893	1.000	0.930 – 1.076	0.990
Gastrointestinal	0.959	0.921 – 0.998	**0.039**	1.104	1.039 – 1.171	**0.001**
Musculoskeletal or Skin	1.025	0.974 – 1.078	0.340	1.093	1.017 – 1.173	**0.014**
Neurological	1.053	0.908 – 1.218	0.489	1.137	0.946 – 1.360	0.165
Psychiatric	1.006	0.959 – 1.054	0.814	1.113	1.049 – 1.181	**<0.001**
Renal or Urological	0.926	0.885 – 0.969	**0.001**	0.839	0.750 – 0.938	**0.002**
Respiratory	0.966	0.910 – 1.025	0.255	1.020	0.947 – 1.098	0.597
Sensory Impairment or Learning Disability	0.894	0.841 – 0.950	**<0.001**	1.016	0.926 – 1.113	0.736
Substance Abuse	1.196	1.074 – 1.329	**0.001**	1.139	1.013 – 1.278	**0.028**

For peer review only

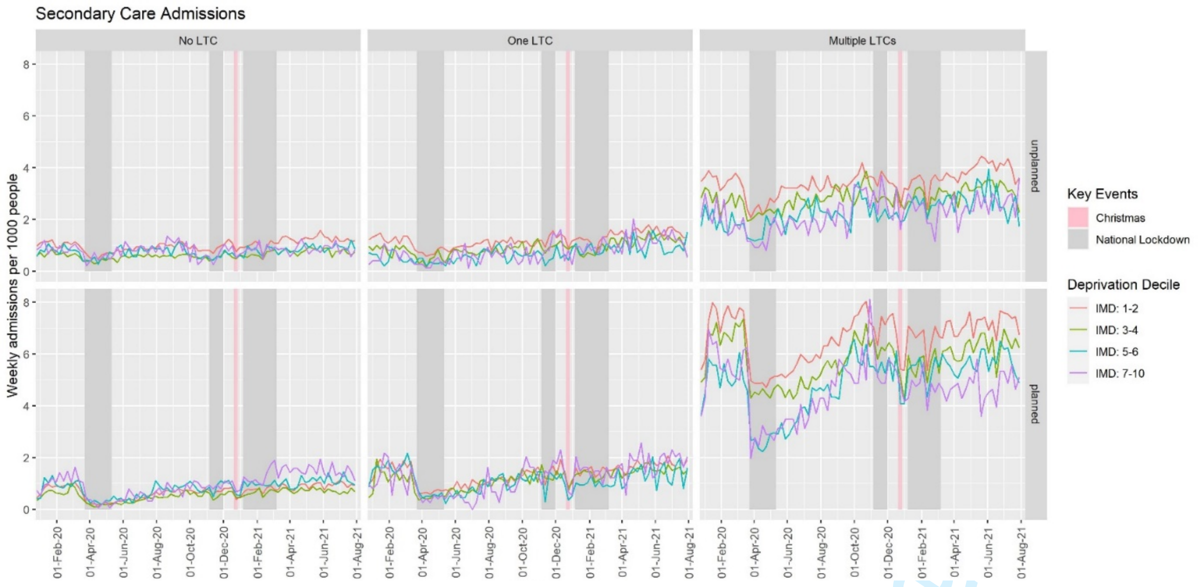

Supplementary Figure S7: Rates of weekly planned and unplanned admissions by deprivation and number of long-term conditions of the Manchester CCG population, between January 2020 and August 2021.

For peer review only

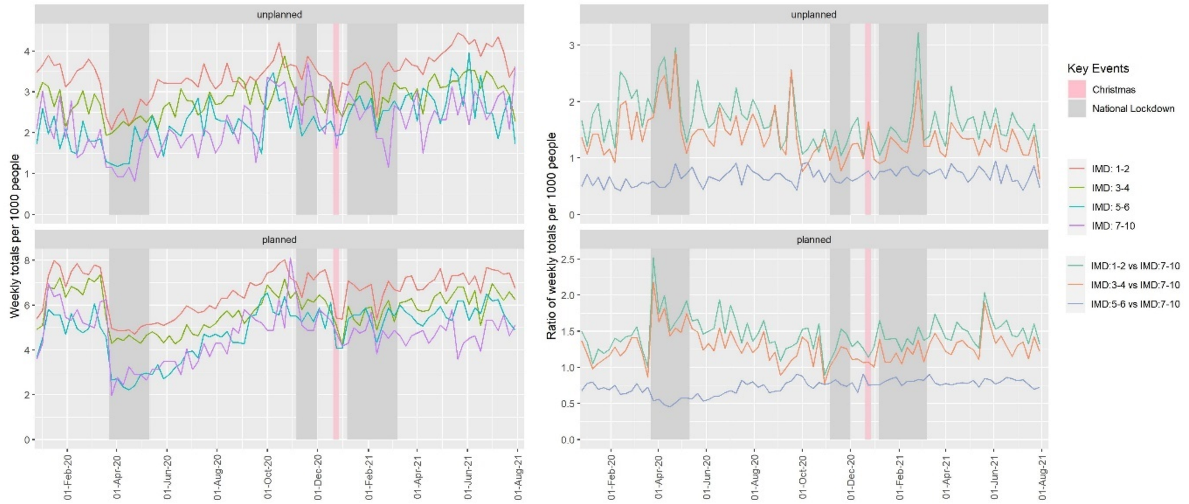

Supplementary Figure S8: Weekly planned and unplanned secondary care admissions per 1000 people within multi-morbid patients by deprivation group and the ratio of these compared to the least-deprived group (IMD: 7-10), between January 2020 and August 2021.

For peer review only

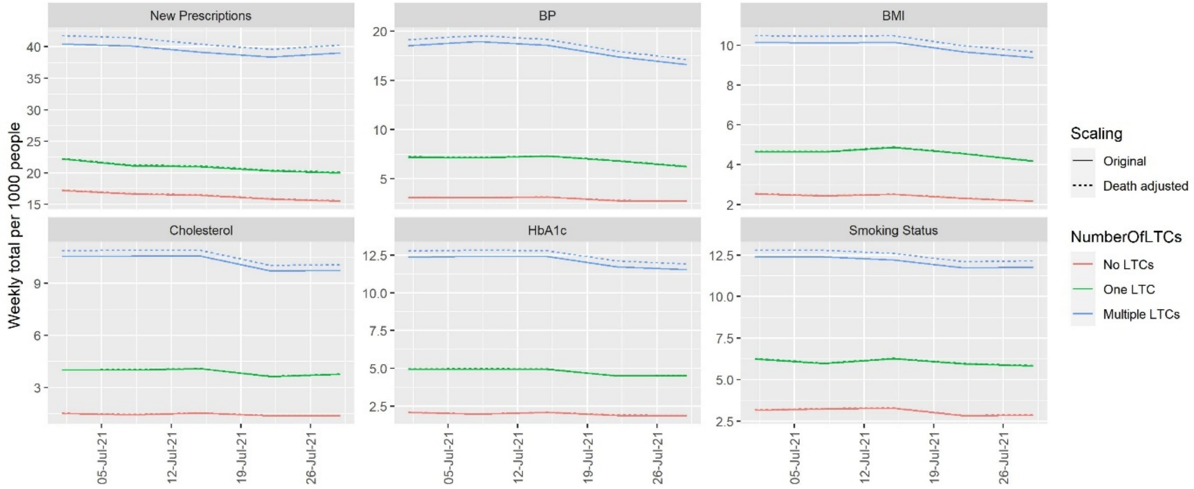

Supplementary Figure S9: Comparison between unadjusted (original) and death-adjusted weekly primary HCU measures per 1000 people, by morbidity in July 2021.

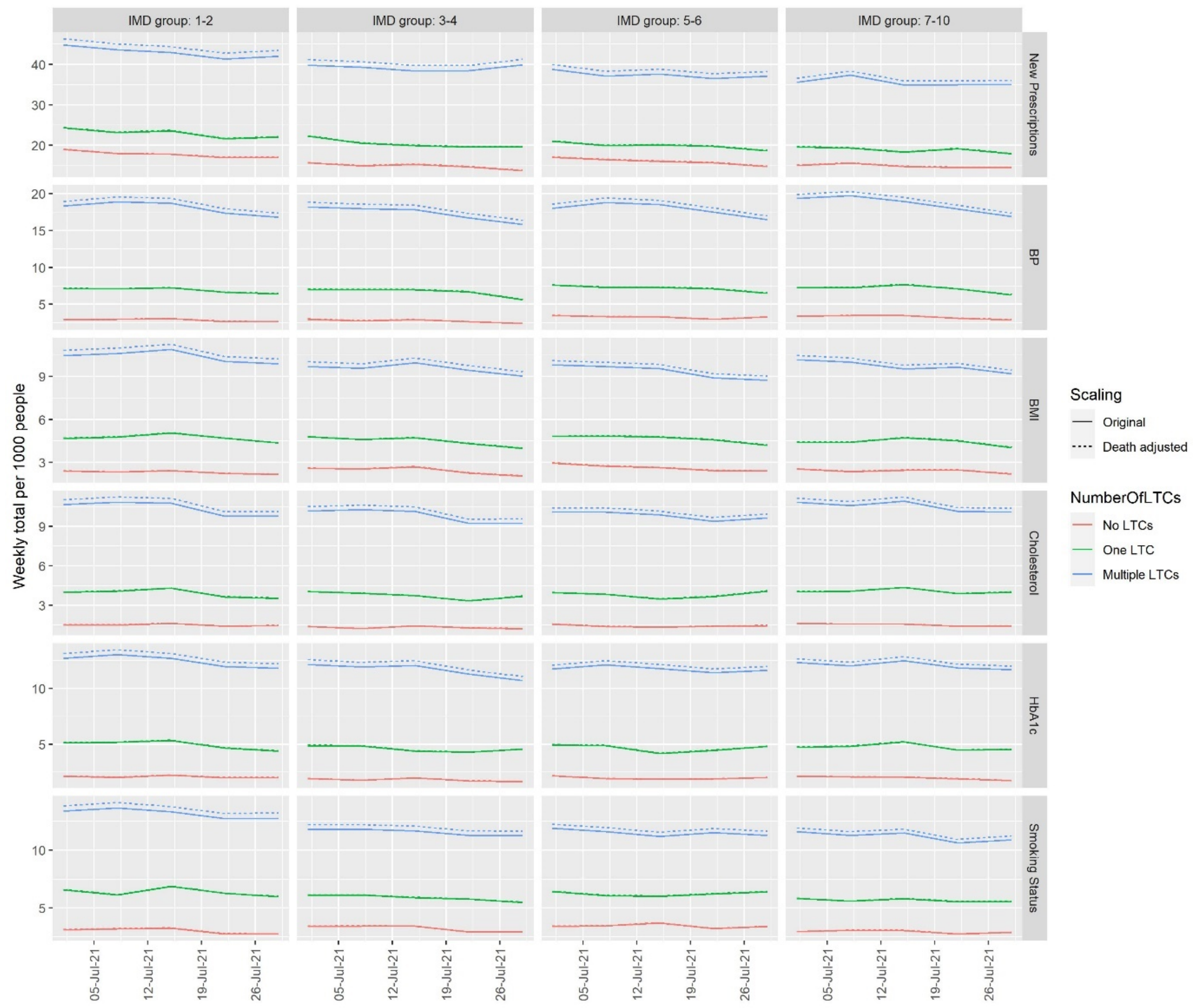

Supplementary Figure 10: Comparison between unadjusted (original) and death-adjusted weekly primary HCU measures per 1000 people, by deprivation group in July 2021.

Supplementary Table S12: Comparison between unadjusted (original) and death-adjusted weekly secondary care admissions per 1000 people, by morbidity group in July 2021.

Number of LTCs (morbidity group)	Admission Type	Week start	Unadjusted Weekly admissions per 1000 people	Death-Adjusted Weekly admissions per 1000 people	Difference
None	unplanned	01/07/2021	1.0235	1.0258	0.0023
None	unplanned	08/07/2021	1.0460	1.0483	0.0024
None	unplanned	15/07/2021	1.0560	1.0584	0.0024
None	unplanned	22/07/2021	0.9934	0.9957	0.0022
None	unplanned	29/07/2021	1.0185	1.0208	0.0023
One	unplanned	01/07/2021	1.4832	1.4898	0.0066
One	unplanned	08/07/2021	1.3576	1.3637	0.0060
One	unplanned	15/07/2021	1.3969	1.4031	0.0062
One	unplanned	22/07/2021	1.1536	1.1587	0.0051
One	unplanned	29/07/2021	1.0987	1.1035	0.0049
Multiple	unplanned	01/07/2021	3.6952	3.8118	0.1166
Multiple	unplanned	08/07/2021	3.7372	3.8551	0.1179
Multiple	unplanned	15/07/2021	3.5932	3.7066	0.1134
Multiple	unplanned	22/07/2021	3.1553	3.2548	0.0995
Multiple	unplanned	29/07/2021	3.1253	3.2239	0.0986
None	planned	01/07/2021	0.9859	0.9882	0.0022
None	planned	08/07/2021	0.9884	0.9907	0.0022
None	planned	15/07/2021	0.8458	0.8477	0.0019
None	planned	22/07/2021	0.9559	0.9581	0.0021
None	planned	29/07/2021	0.8733	0.8753	0.0020
One	planned	01/07/2021	1.6244	1.6317	0.0072

For peer review only

One	planned	08/07/2021	1.6558	1.6632	0.0073
One	planned	15/07/2021	1.8834	1.8918	0.0084
One	planned	22/07/2021	1.4832	1.4898	0.0066
One	planned	29/07/2021	1.7736	1.7814	0.0079
Multiple	planned	01/07/2021	7.1864	7.4131	0.2267
Multiple	planned	08/07/2021	7.0485	7.2708	0.2224
Multiple	planned	15/07/2021	6.8385	7.0542	0.2157
Multiple	planned	22/07/2021	6.8745	7.0914	0.2169
Multiple	planned	29/07/2021	6.3526	6.5530	0.2004

For peer review only

The RECORD statement – checklist of items, extended from the STROBE statement, that should be reported in observational studies using routinely collected health data.

	Item No.	STROBE items	Location in manuscript where items are reported	RECORD items	Location in manuscript where items are reported
**Title and abstract**					
	1	(a) Indicate the study's design with a commonly used term in the title or the abstract (b) Provide in the abstract an informative and balanced summary of what was done and what was found		RECORD 1.1: The type of data used should be specified in the title or abstract. When possible, the name of the databases used should be included.<br><br>RECORD 1.2: If applicable, the geographic region and timeframe within which the study took place should be reported in the title or abstract.<br><br>RECORD 1.3: If linkage between databases was conducted for the study, this should be clearly stated in the title or abstract.	1.1 Data and database specified in the abstract methods (Page 2)<br><br>1.2 Geographic region is specified in the title (Page 1), the timeframe is specified in the abstract methods. (Page 2)<br><br>1.3 No linkage between databases was conducted
**Introduction**					
Background rationale	2	Explain the scientific background and rationale for the investigation being reported			Introduction paragraphs 1-4 (Page 3)
Objectives	3	State specific objectives, including any prespecified hypotheses			Introduction paragraph 4 (Page 3)
**Methods**					
Study Design	4	Present key elements of study design early in the paper			Methods: Design and Data Source (Page 3)
Setting	5	Describe the setting, locations, and relevant dates, including			Methods:

		periods of recruitment, exposure, follow-up, and data collection			Design and Data Source<br>Study Populations and Key Time Points<br>(Pages 3 and 4)
Participants	6	*(a) Cohort study* - Give the eligibility criteria, and the sources and methods of selection of participants. Describe methods of follow-up<br>*Case-control study* - Give the eligibility criteria, and the sources and methods of case ascertainment and control selection. Give the rationale for the choice of cases and controls<br>*Cross-sectional study* - Give the eligibility criteria, and the sources and methods of selection of participants<br><br>*(b) Cohort study* - For matched studies, give matching criteria and number of exposed and unexposed<br>*Case-control study* - For matched studies, give matching criteria and the number of controls per case		RECORD 6.1: The methods of study population selection (such as codes or algorithms used to identify subjects) should be listed in detail. If this is not possible, an explanation should be provided.<br><br>RECORD 6.2: Any validation studies of the codes or algorithms used to select the population should be referenced. If validation was conducted for this study and not published elsewhere, detailed methods and results should be provided.<br><br>RECORD 6.3: If the study involved linkage of databases, consider use of a flow diagram or other graphical display to demonstrate the data linkage process, including the number of individuals with linked data at each stage.	Methods:<br>Long-term medical conditions<br>Index of Multiple Deprivation<br>(Pages 4 and 5)
Variables	7	Clearly define all outcomes, exposures, predictors, potential confounders, and effect modifiers. Give diagnostic criteria, if applicable.		RECORD 7.1: A complete list of codes and algorithms used to classify exposures, outcomes, confounders, and effect modifiers should be provided. If these cannot be reported, an explanation should be provided.	Methods:<br>Long-term medical conditions<br>Index of Multiple Deprivation<br>Measuring Healthcare

For peer review only

					Utilisation (Pages 4 and 5)
Data sources/ measurement	8	For each variable of interest, give sources of data and details of methods of assessment (measurement). Describe comparability of assessment methods if there is more than one group			Measuring Healthcare Utilisation (Page 5)
Bias	9	Describe any efforts to address potential sources of bias			NA
Study size	10	Explain how the study size was arrived at			Study populations and key time points (Page 4)
Quantitative variables	11	Explain how quantitative variables were handled in the analyses. If applicable, describe which groupings were chosen, and why			Methods: Statistical Analysis (Pages 5 and 6)
Statistical methods	12	(a) Describe all statistical methods, including those used to control for confounding<br>(b) Describe any methods used to examine subgroups and interactions<br>(c) Explain how missing data were addressed<br>(d) *Cohort study* - If applicable, explain how loss to follow-up was addressed<br>*Case-control study* - If applicable, explain how matching of cases and controls was addressed<br>*Cross-sectional study* - If applicable, describe analytical methods taking account of sampling strategy			Methods: Statistical Analysis (Pages 5 and 6)

For peer review only

		(e) Describe any sensitivity analyses			
Data access and cleaning methods		..		RECORD 12.1: Authors should describe the extent to which the investigators had access to the database population used to create the study population.<br><br>RECORD 12.2: Authors should provide information on the data cleaning methods used in the study.	12.1 Methods: Design and Data Source (Page 3)<br><br>12.2 NA
Linkage		..		RECORD 12.3: State whether the study included person-level, institutional-level, or other data linkage across two or more databases. The methods of linkage and methods of linkage quality evaluation should be provided.	NA
**Results**					
Participants	13	(a) Report the numbers of individuals at each stage of the study (*e.g.*, numbers potentially eligible, examined for eligibility, confirmed eligible, included in the study, completing follow-up, and analysed)<br>(b) Give reasons for non-participation at each stage.<br>(c) Consider use of a flow diagram		RECORD 13.1: Describe in detail the selection of the persons included in the study (*i.e.,* study population selection) including filtering based on data quality, data availability and linkage. The selection of included persons can be described in the text and/or by means of the study flow diagram.	NA
Descriptive data	14	(a) Give characteristics of study participants (*e.g.*, demographic, clinical, social) and information on exposures and potential confounders<br>(b) Indicate the number of participants with missing data for each variable of interest			Results paragraph 1 (Page 6)

For peer review only

		(c) *Cohort study* - summarise follow-up time (*e.g.*, average and total amount)			
Outcome data	15	*Cohort study* - Report numbers of outcome events or summary measures over time<br>*Case-control study* - Report numbers in each exposure category, or summary measures of exposure<br>*Cross-sectional study* - Report numbers of outcome events or summary measures			Results (Pages 6 – 10)
Main results	16	(a) Give unadjusted estimates and, if applicable, confounder-adjusted estimates and their precision (e.g., 95% confidence interval). Make clear which confounders were adjusted for and why they were included<br>(b) Report category boundaries when continuous variables were categorized<br>(c) If relevant, consider translating estimates of relative risk into absolute risk for a meaningful time period			Results (Pages 6 – 10)
Other analyses	17	Report other analyses done—e.g., analyses of subgroups and interactions, and sensitivity analyses			Supplementary materials
**Discussion**					
Key results	18	Summarise key results with reference to study objectives			Discussion paragraph 1 (Page 10)
Limitations	19	Discuss limitations of the study, taking into account sources of potential bias or imprecision.		RECORD 19.1: Discuss the implications of using data that were not created or collected to answer the	Discussion Strengths &

For peer review only

		Discuss both direction and magnitude of any potential bias		specific research question(s). Include discussion of misclassification bias, unmeasured confounding, missing data, and changing eligibility over time, as they pertain to the study being reported.	Limitations (Page 12)
Interpretation	20	Give a cautious overall interpretation of results considering objectives, limitations, multiplicity of analyses, results from similar studies, and other relevant evidence			Discussion (Pages 10-12)
Generalisability	21	Discuss the generalisability (external validity) of the study results			Discussion Strengths & Limitations (Page 12)
**Other Information**					
Funding	22	Give the source of funding and the role of the funders for the present study and, if applicable, for the original study on which the present article is based			Page 13
Accessibility of protocol, raw data, and programming code		..		RECORD 22.1: Authors should provide information on how to access any supplemental information such as the study protocol, raw data, or programming code.	Page 13

*Reference: Benchimol EI, Smeeth L, Guttmann A, Harron K, Moher D, Petersen I, Sørensen HT, von Elm E, Langan SM, the RECORD Working Committee. The REporting of studies Conducted using Observational Routinely-collected health Data (RECORD) Statement. *PLoS Medicine* 2015; in press.

*Checklist is protected under Creative Commons Attribution (CC BY) license.

For peer review only
